# Treating village newcomers and travelers for trachoma: Results from ASANTE cluster randomized trial

**DOI:** 10.1371/journal.pone.0178595

**Published:** 2017-06-29

**Authors:** Sheila K. West, Beatriz Munoz, Harran Mkocha, Laura Dize, Charlotte A. Gaydos, Bonnie Swenor, Ann-Margret Ervin, Thomas C. Quinn

**Affiliations:** 1 Dana Center for Preventive Ophthalmology, Johns Hopkins University School of Medicine, Baltimore MD, United States of America; 2 Kongwa Trachoma Project, Kongwa, Tanzania; 3 International Chlamydia Laboratory, Johns Hopkins School of Medicine, Baltimore MD, United States of America; 4 Johns Hopkins Bloomberg School of Public Health, Baltimore MD, United States of America; 5 Division of Intramural Research, National Institute of Allergy and Infectious Diseases, NIH, Bethesda MD, United States of America; Mohammed Bin Rashid University of Medicine and Health Sciences, UNITED ARAB EMIRATES

## Abstract

**Trial design:**

Trachoma is targeted for global elimination. Infection rates with *Chlamydia trachomatis* are higher in new arrivals to a community and in travelers who leave for extended periods, suggesting they are sources of re-infection. This community-randomized, clinical trial was designed to determine if a surveillance program that targeted newcomers and travelers, identified weekly, would result in more communities achieving levels of infection of ≤1%.

**Methods:**

52 communities were randomly allocated 1:1 to the control (annual MDA alone if warranted) or intervention arm (annual MDA if warranted, plus a surveillance program to identify and treat newcomers and travelers). In each community, surveys were completed every six months on a random sample of 100 children ages 1–9 years for trachoma and infection. The primary outcome was the proportion of communities in the intervention arm, compared to the control arm, which had a prevalence of infection at ≤1% by 24 months. Registered: clinicaltrials.gov(NCT01767506).

**Results:**

Intervention communities experienced an average of 110 surveillance events per month. At 24 months, 7 (27%) of 26 intervention communities achieved a prevalence of infection ≤1% compared to 4 (15%) of the 26 control communities (odds ratio = 2·6, 95%CI = 0·56–11·9). At 24 months, the average infection prevalence in the intervention communities was 4·8, compared to 6·9 in the control communities (p = ·06).

**Conclusion:**

Despite surveillance programs for community newcomers and travelers, the proportion of intervention communities with a level of infection ≤1% was lower than expected and not significantly different from control communities.

## Introduction

Trachoma is caused by repeated episodes of an ocular infection with *Chlamydia trachomatis*, and remains the leading infectious cause of blindness world-wide. [1] The follicular stage of trachoma, and infection, resides in children ages 1–9 years, and this population group is the reservoir of infection for their communities. [2] Trachoma affects an estimated 51 countries and an estimated 2·2 million are blind or severely visually impaired due to trachoma. [3] The World Health Organization (WHO) has recommended a multifaceted “SAFE” strategy for trachoma control programs. [4] This approach includes Surgery for trichiasis cases (S), Antibiotics to treat the community pool of infection (A), Face washing (F) and Environmental change (E) to sustain reduction in transmission.

Mass treatment with azithromycin decreases the community pool of infection,[5–8] however, the hope that 1–2 rounds of annual mass drug administration (MDA) would be sufficient to eliminate trachoma or infection in most communities has not been realized, and the postulated reasons why infection remains after MDA are varied. One source of infection may be the reintroduction of *C*. *trachomatis* with in-migration of individuals who can bring in infection, and from returning community members who acquire infection outside of the community. This was the primary explanation for re-emergence of infection in two communities in The Gambia.[9] Nomadic populations in sub-Saharan Africa have been felt to have more trachoma than their settled counterparts.[10] In Tanzania, children of families who are new to a previously mass treated community were five times more likely to have infection compared to children whose families were present during MDA.[11] In Ethiopia, travelers outside the village, where interactions occur that might re-infect the children, were more likely to have infection after MDA than non-travelers.[12] A Brazilian study found that housing migrants from endemic areas increased the risk of trachoma in a low endemic region.[13] Once infection rates in the villages become low, these sources could become a significant component of the residual infection and a possible reason why the trajectory of decline is less steep than expected.

In 2012, in Kongwa district of Tanzania, at the end of a five-year trial of MDA, the prevalence of infection had declined to 3·3% and follicular trachoma to 9% in study communities.[14,15] This scenario presented an ideal opportunity to conduct a randomized, community-based, clinical trial to determine if a surveillance and treatment program for newcomers and travelers in a community could continue to accelerate the decline in infection, compared to communities that did not have such a program. To our knowledge, no other study has been done to address the issue of in migration of infection.

The primary aim of the trial was to determine the additional reduction of infection with *C*. *trachomatis* in 26 communities randomized to a newcomer and traveler treatment program plus annual MDA versus 26 communities randomized to annual mass drug administration alone. The primary outcome was the percentage of communities with a prevalence of infection at 1% or less two years after starting the intervention program. The secondary aim was to determine the added benefit in reduction of follicular trachoma (TF) in the 26 intervention communities compared to the 26 communities randomized to control. The secondary endpoint was the percentage of communities with a prevalence of TF at <5% two years after baseline. The endpoint of <5% for TF was chosen because that is the prevalence level at which MDA is no longer recommended.[16] This trial is registered with clinicaltrials.gov (NCT01767506).

## Methods

### Population

The trial was conducted in the Kongwa district, Tanzania, where every community in the previous three years had undergone annual Mass Drug Administration (MDA). Details on the methods were described in detail previously[17] The final survey for a previous cluster randomized trial provided a complete census that only needed updating as well as a survey that provided an estimate of the trachoma and infection prevalence for each study community.[15] The previous trial on coverage rates found no impact on infection with very high coverage versus high coverage, and thus the communities could be combined and re-randomized for this trial.[15] The overall rates for the study communities at the end of that trial were 9% and 3% respectively. In 2012, we re-randomized the communities for the current trial as described below, and enrolled them from February to July 2013 All residents in a study community were eligible for MDA unless the community had infection ≤1% or TF< 5%. For the study surveys every 6 months, all children ages 1–9 years in study communities were eligible for participation and, based on the most recent census in each community, a simple random sample of 100 children was chosen, plus an additional random sample of 20 children for replacement to allow for loss to follow up. The trial is registered with ClincalTrials.Gov: (NCT01767506).

### Randomization of communities and masking

The ASANTE Trial was a cluster-randomized trial, with the community as the cluster. All communities had agreed to be in the trial before any fieldwork was started. The communities were stratified by whether or not infection was 1% or less at the survey prior to the start of the trial. A constrained randomization approach as suggested by Moulton[18] was used to reduce the likelihood of an imbalance of infection across treatment arms. Fifty-two communities were allocated 1:1, 26 to each arm, using the allocations that meet the balance criteria, and employing a SAS Macro (SAS, Carey NC). The senior biostatistician (BM) conducted the randomization and was the only person to have access to the assignment until all communities were assigned. Once all were assigned, the biostatistician provided a master list prior to any fieldwork to the Project Director, who implemented the randomization as assigned.

Because of the nature of the intervention, masking of the communities was not feasible after randomization. The laboratory, however, was masked and received specimens with no indication of assignment. The survey team members were independent of the other teams, and masked to the assignment; however, during field data collection, the survey team could have been unmasked.

### Sample size

This trial had a fixed sample size of 52 communities. Power calculations were based on the primary outcome, proportion of communities with prevalence of *C*. *trachomatis* of ≤1% at 24 months post baseline survey. A two-sided Fisher’s exact test and a significance level of α = 0·05 were used to estimate the power. We made the following assumptions, based on previous data on the decline in infection and our study on the increased risk of in migration[11]: We observed an average starting prevalence of infection of 3% and expected to observe a decrease in the infection rate by half after one year. We expected that 8 of the 26 communities (p_0_ = 0·308) in the control arm would have a prevalence of infection of ≤1% by 24 months, accounting for re-emergence observed in other studies. We had 80% power to detect differences if the number of communities at or below 1% in the intervention arm was at least 19 of 26, or 2·4 times or more (i.e. p_1_≥0·731 or p_1_/p_0_≥2·38) than the communities in the control arm after two years. This difference, if we found it, was felt to be of sufficient significance to warrant consideration of a surveillance program.

### Census

A complete census was performed prior to the baseline survey with updates annually. The census was used to collect information on each household and to provide information for the selection of households with eligible children for the survey within the community. Additionally, the census was used as the database for mass treatment. A household was defined as a unique doorway belonging to a resident; a resident in the household was defined as a person who had slept in the household for at least three months (or if age less than 3 months, was born into the household) or who intended to reside with the family for the next six months. For each household, the census team member obtained a list of the names, ages, and gender of all persons resident in the household. The ages of the children were determined from vaccination or Maternal and Child health clinic cards if possible. Other demographic information collected included education, completed by the head of the household, distance to the closest source of water, observations on clean face status of children aged 5 and under, and presence of latrines. At census updates, the same census information was collected for each household. If the household or person was new to the community since the last census, these individuals were asked how long they lived in their present location.

### Survey

Surveys for trachoma, and infection based on a laboratory test of an ocular swab, were done at baseline and every 6 months to 24 months. The procedures were always the same. Based on the census (or updated census), a random sample of 120 children ages 1 to 9 years were selected from each community. A trained trachoma grader, using a flashlight and 2·5 loupes, assessed each eyelid for the presence or absence of trachoma inflammation-follicular (TF) and trachoma inflammation-intense (TI) using the World Health Organization simplified grading scheme.[19] An ocular photograph, taken of the right eye of every 5th child plus all children with trachoma, ensured at least 50 photographs for purposes of monitoring drift in grading over time. A handheld Nikon D-series camera (D-40) with a 105mm f/2·8D AF Macro Nikkor Autofocus Lens (fully extended, in manual setting) was used.

Following a strict protocol to avoid field contamination, a swab was taken of the left eye of every child, stored dry in a refrigerator for up to 30 days, shipped to Johns Hopkins University International Chlamydia laboratory, where it was stored at -80° until processed. In addition, a negative field control ("blue air swab") was taken on a randomly chosen 5% sample to monitor contamination. For each negative field control, the examiner will pass a sterile Dacron swab within 1 inch of the individual’s conjunctiva, and these were labeled and processed identically to true conjunctival swabs. The laboratory personnel were masked to intervention and control arms, and to field control and regular swabs.

### Laboratory processing

The swabs were processed using a published pooling strategy[20,21] (4–5 per pool) within 90 days of arrival, using the APTIMA Combo 2 (AC2) commercial test for *C*. *trachomatis* (Hologic/Gen-Probe Inc., San Diego CA). The results were recorded as positive or negative for *C*. *trachomatis*, equivocal, or invalid. Equivocal or invalid pools were repeated. For pools that yielded a negative result, all specimens in that pool were recorded as negative for *C*. *trachomatis*. For each pool that yielded a positive result, the original samples were retested in order to determine which sample (s) had a positive result. Equivocal pools were retested as well as the individual samples contained in that pool, to determine a final positive or negative result.

### Mass drug administration (MDA)

MDA in all communities was carried out by a network of Community Drug Distributors (CDDs) who were trained community residents. The treatment team had a list of all community residents, based on the latest census update, which was used to note who received drug and to calculate treatment coverage. The coverage target was 80% of children treated. A community found to have decreased infection to ≤1% or TF <5%, on the basis of a survey prior to baseline or at 6 or 18 months, was eligible to have cessation of MDA at the next scheduled round. According to our Data and Safety Monitoring Committee recommendations, if a community which had MDA stopped experienced a re-emergence of infection to 6% on either the 6 or 18-month survey, MDA was to be re-started at the next annual cycle. In the intervention arm, even though infection may have decreased to 1% or TF <5%, the surveillance and treatment program was still active and treatment within the surveillance program for eligible families continued per protocol.

All communities were provided two days of mass treatment in their neighborhoods. More treatment days were scheduled depending on the tally of coverage at the end of the two days. The dosage of azithromycin was 20mg/kg, up to 1 gm in a single dose of either liquid or tablets. In Tanzania, pregnant women can be treated with azithromycin, but children under 6 months are given topical tetracycline. After each community completed mass treatment, treatment verification occurred in a random sample of five households among the households assigned to each CDD. A staff treatment supervisor visited each of the five homes after mass treatment, and asked the head of the household to verify the treatment status of each member of the household. The staff member was unaware of the treatment status at the time of the visit, and this was reconciled in the office.

### Intervention

In the 26 communities randomized to the intervention arm, in addition to MDA (if warranted) a community-based Surveillance and Treatment Program (STP) was instituted. The STP program was designed to be simple and locally appropriate, but the monitoring of the program was intense to be certain it was implemented as planned for the trial. Local Community Monitors (CMs) were hired, trained, and given defined areas in their communities to survey each week, going house to house. They identified newcomer families, and resident families that have traveled outside the community for at least eight weeks, and offer azithromycin treatment to these families. Treatment was provided regardless of MDA status of the community. We defined “newcomer” as any guardian with at least one child under age 10 years that migrated to the community and planned to live either with an existing family or in a new home for at least one month. Newcomers received treatment if they came from a community that had not received MDA in the last year. We defined “traveler” as any family with children under age 10 years that returned to the community, having left for eight weeks or more. If travelers left for another community that had not received MDA in the last year, they received treatment upon their return. The CMs used the latest census to verify that each household within their defined areas was present at the last verification during the previous week, and whether any new family must be added. The CMs in a community were monitored for a week each month by supervisors who had to verify all surveillance events, as defined above, spot check a sample of households with no event, and visit all households where a newcomer or traveler “event” was recorded. Payment to the CM for case finding was contingent on correct identification and treatment.

Of note, the district did not have any formal trachoma control activities related to facial hygiene or environmental improvements ongoing during the trial, apart from radio spots from the National program.

### Adverse events

The Community Health Workers collected data on adverse events after each newcomer and traveler family was identified and treated. There were no serious adverse events, and all were reviewed by a physician monitor.

### Data management

All census data were double entered into a custom-built Access database, where range and consistency checks were instituted. Survey data and Community Monitor supervisor validated data were entered into Samsung tablets (Samsung Electronics, Seoul, Korea) that also included range and some consistency checks. Tablet data were uploaded to a custom built Access databases as well, to allow data checking before exporting. All data were encrypted and sent to the Data Coordinating Center at Johns Hopkins where further data cleaning, using SAS (Carey, NC) for checking across forms, was undertaken.

### Data analyses

All analyses were conducted on intent-to-treat basis. Population characteristics were measured at the child and household level. These were summarized at cluster (community)-level and then summarized by treatment assignment. The median of cluster means for each arm were presented, along with the median (IQR) of cluster medians for each characteristic. The Wilcoxon rank sum test was used to compare the two groups.

*C*. *trachomatis* infection/follicular trachoma prevalence are presented by randomization arm, and the proportion of communities at or below the threshold for infection/ follicular trachoma were estimated. Differences between arms in the proportion at or below the threshold at 24 months were tested using a two-sided Fisher’s exact test as specified in sample size determination.

Multivariable models were constructed at the community level. For the primary analysis, logistic regression was used to model the probability of a community being at or below threshold for infection/follicular trachoma at the 24-month survey adjusting for baseline infection/follicular trachoma. Linear mixed models with random intercept and slope were used to estimate the yearly change in infection/trachoma by randomization arm.

### Ethics statement

All procedures and protocols, including verbal consent as described below, were approved by the Johns Hopkins University Institutional Review Board and the Tanzania National Institute for Medical Research. The research was conducted according to the principles expressed in the Declaration of Helsinki. Community leaders were approached about participation in their communities in the trial after randomization, and all who were approached agreed to allow us to approach community members. Written informed consent was obtained from all guardians of children in the surveys, and verbal assent obtained from children of school age. Verbal informed consent was obtained from all newcomers and travelers to an intervention community who received a single dose of azithromycin outside of the community MDA, documented by the community health workers on the medication distribution sheet. Written consent was not obtained, as azithromycin is normally provided as part of community MDA and, community leadership did not want procedures different than those used by programs. Further details on consent procedures were presented previously[17].

## Results

**Fig 1:**
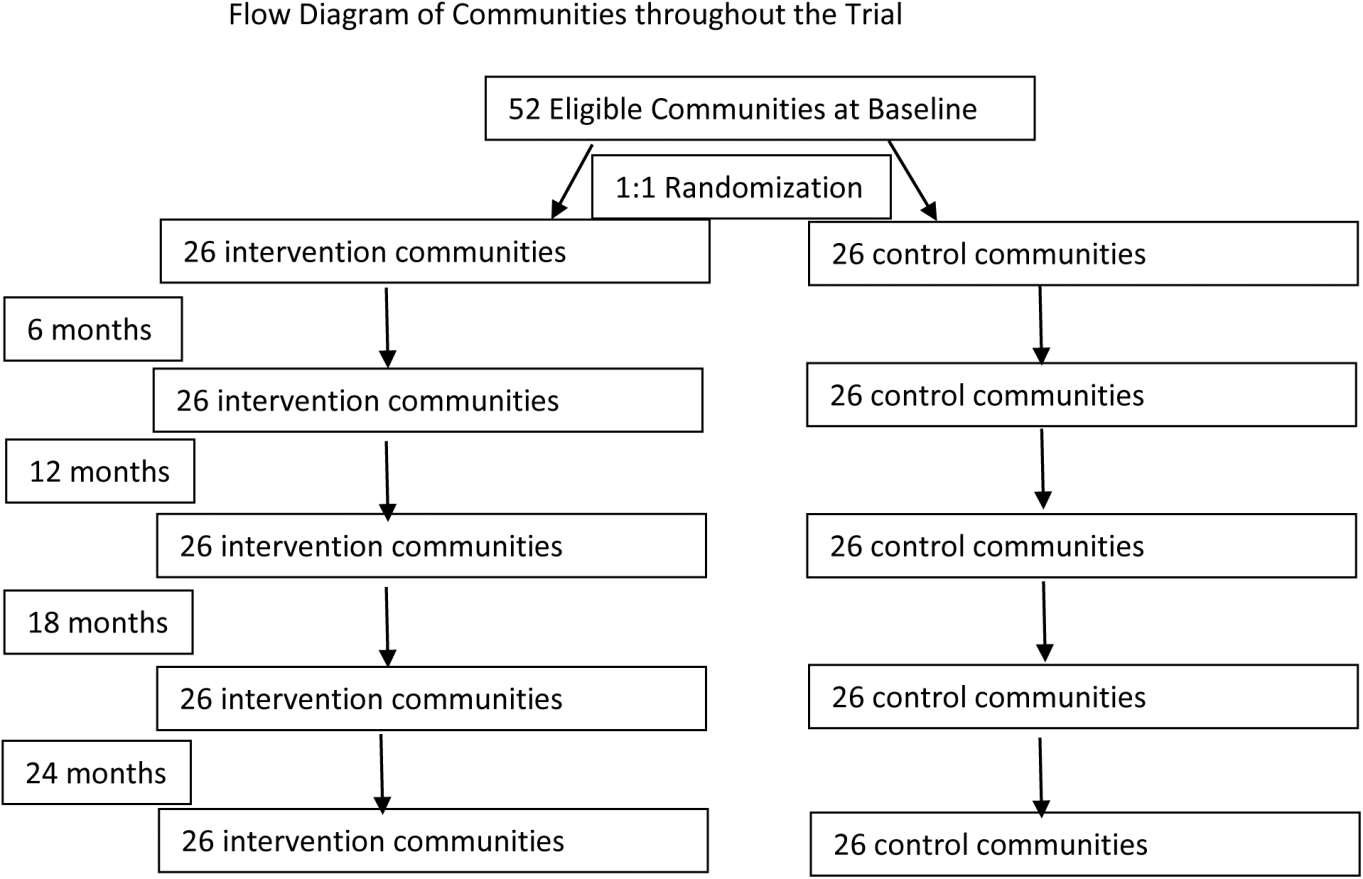
CONSORT flow diagram.

All 52 communities approached for the trial were randomized, and enrolled from January 2013 to August 2013, and followed throughout the trial with the last follow up in November (Fig 1). The communities in each arm were well-balanced in terms of characteristics (Table 1). The communities in the intervention arm were slightly larger, and the percentage of households with latrines was fewer. In both arms, around 45% of households had a bicycle, and more than half lived greater than 30 minutes from a water source. There was no difference at baseline in the trachoma rates or infection rates by study arm.

**Table 1:** Baseline characteristics of study communities, by randomization arm.

Characteristic	Intervention Arm (N = 26) Median (IQR)	Control Arm (N = 26) Median (IQR)	P
Total population size	1664 (1297–1997)	1437 (1246–1887)	0.21*
Years of education of Head of Household	3.5 (2.9–4.4)	4.0 (3.0–4.5)	0.30*
% of households >30 minutes from water	51 (33–78)	52 (37–71)	0.99*
% of households with latrines	80 (71–85)	82 (74–89)	0.30*
% of households with a bicycle	45 (34–52)	45 (37–52)	0.91*
% communities with infection ≤ 1%	6/26 (23.1)	4/26 (15.5)	0.73**
% communities with Follicular Trachoma	16/26 (61.5)	15/26 (57.7)	1.00**
(TF) <5%			
Prevalence of infection in 1–9 year olds	3.0 (1.8–4.8)	3.0 (1.3–5.3)	0.88*
Prevalence of Follicular TF in 1–9 year olds	4.4 (2.6–7.5)	3.8 (1.9–7.5)	0.62*

*Wilcoxon Two-Sample Test (two-sided)

**Fisher’s exact test (two sided)

Over the course of two years, 2647 surveillance events occurred, or an average of 110 per month in the intervention arm (Table 2). In the first year, the surveillance team verified close to 1300 events, of which most were either new households or new families joining existing households. In the second year, the number of events increased to 1370, and most were new families joining existing households. New households or new families in existing households represented roughly on average 10% to 17% of the population of a community, although this is a dynamic measurement In 91% of events, all eligible persons were treated (range 78%-100%).

**Table 2:** Surveillance events in the 26 communities in the intervention arm.

Type of Surveillance event	Year 1		Year 2		Total	
	N	Average/Com*	N	Average/Com	N	Average/Com
New households	544	21.3	514	19.8	1068	41.1
Traveler families	181	7.0	218	8.4	399	15.4
New family in existing household	542	20.8	638	24.5	1180	45.3
Total	1277	49.1	1370	52.7	2647	101.8

*Com = Community

**Fig 2:**
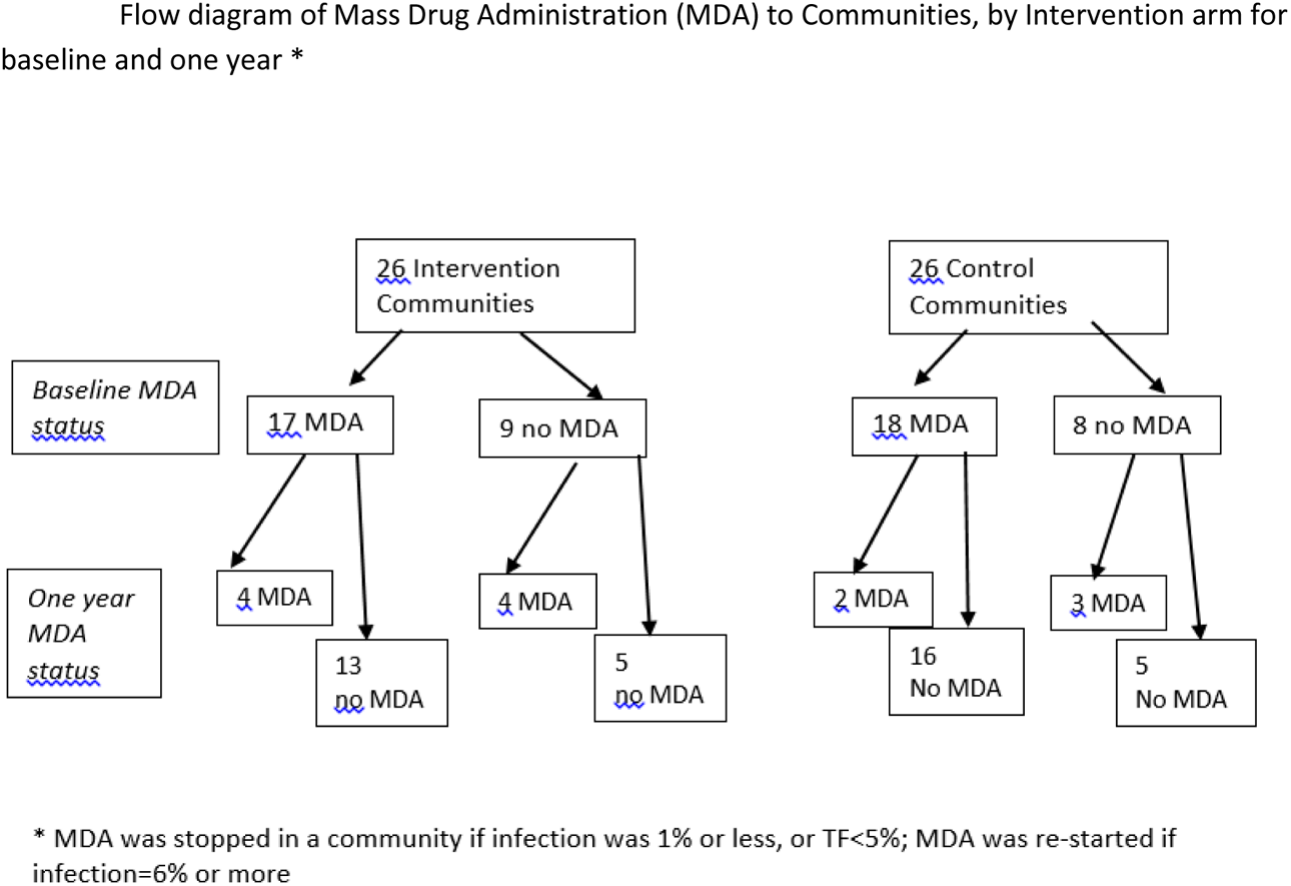
Intervention versus control from baseline to one year.

The average coverage with MDA in children, among the communities who received MDA, was similar for both groups over time (Table 3). At baseline, 9 of the 26 communities in the intervention arm and 8 of the 26 communities in the control arm stopped MDA because their infection rates were ≤1% or TF<5% in the previous survey. Based on the six-month data, 8 of the 26 communities in the intervention arm and 5 of the 26 communities in the control arm received MDA at one year. From baseline to one year, 4 of 9 communities in the intervention arm and 3 of 8 communities in the control arm who did not have MDA at baseline restarted MDA, because of an increase of infection to 6%, our pre-specified level for re-starting MDA (Fig 2).

**Table 3:** Treatment coverage, by randomization arm.

	# of (communities/households) and coverage, by year	Intervention	Control	P
	Number of communities	26	26	
Annual MDA*	Baseline (number of communities treated)	17	18	
	Coverage of MDA in 1–9 year olds	86.9%	86.8%	0.95
	One year (number of communities treated)	8	5	
	Coverage of MDA in 1–9 tear olds	85.6%	82.8%	0.17
Treatment during surveillance	First year (number of treatment eligible households)	756	NA	
	Proportion receiving treatment	87.0%		
	Second year (number of treatment eligible households)	749		
	Proportion receiving treatment	94.7%		

*communities with infection of 1% or less, or TF<5% were not eligible for MDA

**Fig 3:**
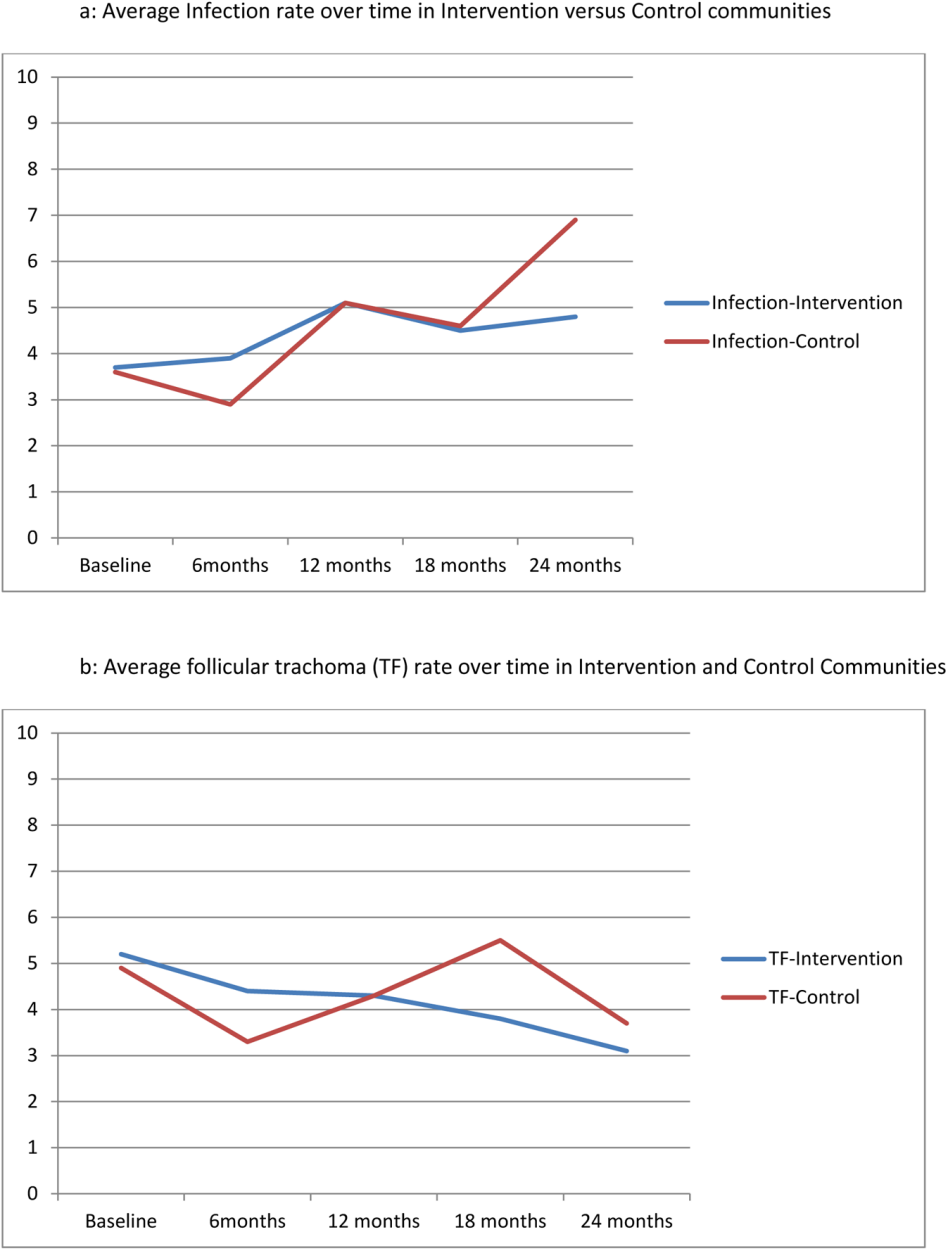
Average infection rate over time in intervention versus control communities and average follicular trachoma (TF) rate over time in intervention and control communities.

At 24 months, 7 (27%) of 26 intervention communities achieved a prevalence of infection ≤1% compared to 4 (15%) of the 26 control communities (odds ratio = 2·6, 95%CI = 0·56–11·9). The difference of 11·5% (exact 95% CL (-17·4%, 39·1%)) was not statistically significant (Fisher’s exact test (p = 0.49). Table 4 shows no difference when adjusted for the baseline prevalence of infection (p = 0.22). Fig 3A and 3B present the average prevalence of infection and trachoma over time by randomization arm. The two arms began with very similar prevalence of infection, 3·7% and 3·6%. Both groups had worse infection over time, although the increase from 12 to 24 months appeared to be more steep in the control communities; the estimated yearly change of infection in the control communities was 1·7%, compared with 0·5% in the intervention communities (p = 0·06). The trachoma prevalence over time decreased slightly, from 5·2% to 3·1% in the intervention communities, and from 4·9% to 3·7% in the control communities. The estimated yearly decrease in trachoma in the control communities was -·04%, compared with -0·9% in the intervention communities, and this difference in the rate of change between control and intervention communities was borderline significant (p = 0·05). At 24 months, the proportion of communities with trachoma prevalence <5% was essentially equivalent, 80·8% versus 76·9%, a difference of 3·9% (p = 1.00, Fisher’s exact test) and no different when adjusted for the baseline prevalence of trachoma (p = 0.75) (Table 4).

**Table 4:** Model predicting communities with infection ≤1% at 24 months and follicular trachoma less than 5% at 24 months.

Model Characteristic: Infection	Odds Ratio	95% CI	P value
Baseline infection prevalence (per 1% increase)	0·60	0·37–0·95	0·03
Odds of Intervention vs control arm being at ≤1%	2·61	0·57–11·9	0·22
Model Characteristic: **Follicular Trachoma**			
Baseline TF prevalence (per 1% increase)	0·74	0·6–0·91	·004
Odds of Intervention vs control arm being at <5%	0·79	0·17–3·58	0·75

## Discussion

We instituted a community-based surveillance and treatment program in randomly assigned communities in Kongwa district Tanzania, where infection had decreased to <4% and prevalence of TF had decreased to <10%. The hypothesis was that we could further decrease infection to 1% or less in more of the communities in the surveillance intervention arm, compared to control communities. However, despite the intervention program, we did not find a statistically significant difference in the proportion of communities in the intervention arm that had infection at 1% or less compared to the proportion in the control communities. While we found a lower rate of infection in the intervention communities at 24 months (4·8%) compared to the control communities (6·9%), the difference was not statistically significant (p = ·06).

Our original sample size calculation was based on literature suggesting that when infection is low, more communities in the intervention and control arms would reach a prevalence of ≤1% infection than actually occurred. The difference of 12% was smaller than anticipated. While the intervention communities had no significant increase in infection from baseline to 24 months, the control communities had an increase, but the difference in slopes from baseline to 24 months was of borderline significance between the two arms (p = ·06). We appreciated that even a simple surveillance system when implemented in each village at district level would be a sizeable effort, as we described in the methods. Our project had one Community Monitor per 20–30 households, who visited their households once per week. This required a time commitment of less than the equivalent of a single day so as to not seriously compromise other work, and they were paid the equivalent of $1 per week to do this. At scale, without consideration of supervision or training, this would require on the order of 3200 community monitors for Kongwa district alone. Outside of a research setting, a more practical approach would likely be targeting villages where in-migration from known endemic areas was greatest, for example those villages on the border with the northern region. Nevertheless, to recommend such an approach, the difference in infection rates would need to be sizable, at least the 30% as we anticipated from our previous data, to justify such an effort[11]. The difference between 27% of communities with infection ≤1% compared to 15% of communities in the control arm may not be sufficient justification.

There was good evidence from prior research to support our original hypothesis. Introduction (or re-introduction) of *C*. *trachomatis* from in-migration of individuals who have infection, and from returning community members who acquire infection outside, has been shown by us and others.[9,11,12] Are other sources of infection more important than controlling infection coming from outside the community via newcomers and travelers? Some have argued that young children are an important source of re-emergent infection because they are treated (and likely unevenly) with topical tetracycline. Infants less than 6 months of age are not eligible for azithromycin, and may have very high chlamydial burdens.[7] However, our study in Tanzania, which addressed this issue, found that infections were few in this age group and that households with infants were not at increased risk of infection following mass treatment.[22] In this setting, which at baseline had very low levels of infection, it is unlikely that uneven treatment of infants is a major source of infection. Another source may be persons with high burdens of infection. In some studies, persons who had infection prior to treatment, and who had the greatest infectious burden prior to treatment, were also at risk of infection post-treatment.[8]^,^ [23] In another study which found no evidence of resistance of chlamydia to azithromycin, the load of infection pre-treatment was not predictive of infection post treatment.[24] The communities in the current study have come from a previous study of 3 years of annual MDA, already had low infection rates to start, and thus are unlikely to have many persons with high burden of infections, although we did not study infectious burden.

While we believe that the significant effort put into supervision of the surveillance program succeeded, one possible limitation is the design of the program itself. We did not plan to detect newcomers who were solely children, as we reasoned it would be unlikely they would arrive without a guardian to an existing house. We know of instances when grandmothers picked up children from elsewhere to take care of them, but these were not strictly new child and guardians, so these children were not tracked. Movement of children alone within a village was not of interest, as they would not have brought in infection from outside. Also, we defined traveling families as those whose members all left, and were gone for a continuous eight weeks. Some members who travel, or those who left for less than eight weeks, would not be tracked. This definition was deliberate, to avoid having to track the considerable number travel for many for a short period of time to a farm, where it is unlikely the family would encounter anyone else. These short trips would have placed a significant burden on a surveillance program. We may have missed infections brought in by those who did not meet our criteria for surveillance, although we felt this was minimal. Moreover, the effort and cost of mounting a surveillance program that intensive without more evidence it would be effective may not be justified.

Despite our earlier finding of higher rates of infections in neighborhoods where newcomers settled[11], we cannot rule out the possibility that newcomers and travelers came from areas of low infection and disease, and thus did not contribute to the change over time.

We must also interpret the data in light of the significant numbers of study communities who did not have MDA at baseline and one year, because as communities they had reached a prevalence of trachoma <5%. All these communities came into this trial having had three rounds of MDA with high coverage from a previous trial a year prior. At baseline, 17 of them did not have MDA and of these, 7 (41%) had to re-start MDA based on infection re-emerging to 6% (our pre-specified rule). These data suggest that, in a district that had not yet reached <5% trachoma prevalence, stopping MDA in individual communities or even sub-districts who have reached that prevalence is not advisable as the risk of re-emergence of infection appears to be higher than anticipated.

In summary, despite a surveillance and treatment program for community newcomers and travelers to prevent reemergence of infection, the proportion of intervention communities who achieved a level of infection ≤1% was lower than expected and not significantly different from control communities. Targeting potential incoming sources of infection alone may not be justified.

## Supporting information

S1 File CONSORT checklist. (DOC)

S2 File Manual of Procedures. (DOCX)
