# Supplementary material for: Treating village newcomers and travelers for trachoma: Results from ASANTE cluster randomized trial
Source: PLoS One. 2017 Jun 29;12(6):e0178595. doi: 10.1371/journal.pone.0178595 (PMC5490954; doi:10.1371/journal.pone.0178595)
Supplement: S2 File — (DOCX) [file pone.0178595.s002.DOCX]

Version

4

A Surveillance and Azithromycin Treatment for Newcomers and Travelers Evaluation:

The ASANTE Trial

Manual of Procedures

© Johns Hopkins Medical Institutions

600 N Wolfe St Baltimore MD, 21205

Tel: (410) 955-2606

FAX: (410) 955-0096

Table of Contents

[Chapter 1: Overview 8](#_Toc337159785)

[1.1 Overview 8](#_Toc337159786)

[1.2 Research Aims 9](#_Toc337159787)

[1.3 Overall Study Design 11](#_Toc337159788)

[1.4 References 11](#_Toc337159789)

[Chapter 2: Background and Significance 13](#_Toc337159790)

[2.1 Background 13](#_Toc337159791)

[2.2 Preliminary Studies 16](#_Toc337159792)

[2.3 Impact 17](#_Toc337159793)

[2.4 References 18](#_Toc337159794)

[Chapter 3: Randomization and Sample Size 22](#_Toc337159795)

[3.1 Randomization of Communities 22](#_Toc337159796)

[3.2 Sample Size 22](#_Toc337159797)

[3.2.1 Communities 22](#_Toc337159798)

[3.2.2 Children 25](#_Toc337159799)

[3.2.3 Women 25](#_Toc337159800)

[3.3 References 25](#_Toc337159801)

[Chapter 4: Intervention 26](#_Toc337159802)

[4.1 Overview 26](#_Toc337159803)

[4.2 Newcomer and Traveler Treatment Intervention 26](#_Toc337159804)

[4.3 Monitoring Treatment Coverage 28](#_Toc337159805)

[4.3.1 Monitoring Mass Treatment Coverage 28](#_Toc337159806)

[4.3.2 Monitoring Newcomer and Traveler Treatment Coverage 29](#_Toc337159807)

[4.4 Masking Intervention 30](#_Toc337159808)

[Chapter 5: Eligibility and Exclusion Criteria 31](#_Toc337159809)

[5.1 Eligibility Criteria: Communities 31](#_Toc337159810)

[5.2 Eligibility Criteria: Children 31](#_Toc337159811)

[5.3 Eligibility Criteria: Women 31](#_Toc337159812)

[5.4 Exclusion Criteria 31](#_Toc337159813)

[Chapter 6: Consent Procedures 33](#_Toc337159814)

[6.1 Background 33](#_Toc337159815)

[6.2 Consent Procedures 33](#_Toc337159816)

[6.3 Risks and Efforts to Minimize Risk 36](#_Toc337159817)

[Chapter 7: Census Procedures 37](#_Toc337159818)

[7.1 Overview 37](#_Toc337159819)

[7.2 Baseline Census 37](#_Toc337159820)

[7.3 Newcomer and Traveler Census Procedures 38](#_Toc337159821)

[7.3.1 Newcomers Census 39](#_Toc337159822)

[7.3.2 Traveler Census 39](#_Toc337159823)

[7.4 Census Update Procedure 40](#_Toc337159824)

[7.4.1 Surveys and Mass Treatment Updates 40](#_Toc337159825)

[7.4.2 Yearly Census Updates 40](#_Toc337159826)

[Chapter 8: Study Visits 41](#_Toc337159827)

[8.1 Overview 41](#_Toc337159828)

[8.2 Community Level Visits 41](#_Toc337159829)

[8.3 Survey Visits 42](#_Toc337159830)

[8.3.1 Trachoma Assessment 43](#_Toc337159831)

[8.3.2 Ocular Photography 45](#_Toc337159832)

[8.3.2.1 CAMERA SETUP 45](#_Toc337159833)

[8.3.2.2 PHOTOGRAPH PROCEDURE 46](#_Toc337159834)

[8.3.2.3 PHOTO TROUBLESHOOTING GUIDE 46](#_Toc337159835)

[8.3.3 Laboratory Specimen Collection 47](#_Toc337159836)

[8.3.3.1 PROTOCOL FOR SWABBING OF THE CONJUNCTIVA 47](#_Toc337159837)

[8.3.3.2 PCR CONTROL SWABS 48](#_Toc337159838)

[8.3.3.3 PROTOCOL FOR TUBING AND HANDLING OF SAMPLES 49](#_Toc337159839)

[8.3.3.4 SHIPPING AND STORAGE OF SAMPLES TO JHU LABORATORY 49](#_Toc337159840)

[8.4 Follow-up of Children 50](#_Toc337159841)

[8.5 Forms and Swab Management for Visits 51](#_Toc337159842)

[8.6 Follow-Up Visits and MDA 51](#_Toc337159843)

[8.7 Missed Visits 52](#_Toc337159844)

[8.8 References 52](#_Toc337159845)

[Chapter 9: Laboratory Analyses 53](#_Toc337159846)

[9.1 Laboratory Processing Procedures 53](#_Toc337159847)

[9.1.1 Testing JHU Laboratory 53](#_Toc337159848)

[9.1.2 Pooling Algorithm 53](#_Toc337159849)

[9.1.3 Assay Procedure 54](#_Toc337159850)

[9.1.4 Interpretation of Assay Results 54](#_Toc337159851)

[9.1.5 Quality Control Results and Acceptability 55](#_Toc337159852)

[9.1.6. Reporting Results to Data Coordinating Center 55](#_Toc337159853)

[9.2 References 55](#_Toc337159854)

[Chapter 10: Mass Treatment Administration 57](#_Toc337159855)

[10.1 Mass Treatment with Azithromycin 57](#_Toc337159856)

[10.1.1 Mass Treatment Verification Procedure 59](#_Toc337159857)

[10.1.2 Stopping Rule Implementation 59](#_Toc337159858)

[10.2 References 60](#_Toc337159859)

[Chapter 11: Study Instruments 61](#_Toc337159860)

[11.1 Overview 61](#_Toc337159861)

[11.2 Census Forms 61](#_Toc337159862)

[11.2.1 Add New Household to Census Form (Form 1) 61](#_Toc337159863)

[11.2.2 Census Update Book (Form 2) 64](#_Toc337159864)

[11.3 65](#_Toc337159865)

[Cooking Fire Questionnaire 65](#_Toc337159866)

[11.4 Ocular Exam Forms 69](#_Toc337159867)

[11.4.1 Survey List (Sentinel List) 69](#_Toc337159868)

[11.4.2 Ocular Exam Form (Form 3) 69](#_Toc337159869)

[11.4.3 Photo Log Book (Form 4) 78](#_Toc337159870)

[11.4.4 Swab Log Book (Form 5) 78](#_Toc337159871)

[11.5 Treatment Forms 79](#_Toc337159872)

[11.5.1 Mass Treatment Book (Form 6) 79](#_Toc337159873)

[11.5.2 Mass Treatment Verification Form (Form 7) 81](#_Toc337159874)

[11.5.3 Community Monitor Household Checklist (Form 8) 81](#_Toc337159875)

[11.5.4 Newcomers & Travelers Treatment Form (Form 9) 82](#_Toc337159876)

[11.5.5 KTP Supervisor Form (Form 10) 83](#_Toc337159877)

[Chapter 12: Training and Certification of Staff and Clinics 85](#_Toc337159878)

[12.1 Introduction 85](#_Toc337159879)

[12.2 IRB Compliance 85](#_Toc337159880)

[12.3 Field Training and Certification 85](#_Toc337159881)

[12.3.1 Trachoma Grading 85](#_Toc337159882)

[12.3.2 Photography 86](#_Toc337159883)

[12.3.3 Laboratory Specimen Handling 87](#_Toc337159884)

[12.3.4 Study Forms 87](#_Toc337159885)

[12.3.5 Data entry 87](#_Toc337159886)

[12.3.6 Intervention and Supervision of Intervention 88](#_Toc337159887)

[12.4 References 89](#_Toc337159888)

[Chapter 13: Quality Assurance Policies and Procedures 90](#_Toc337159889)

[13.1 Overview 90](#_Toc337159890)

[13.2 General Guidelines for Form Completion 91](#_Toc337159891)

[13.3 Missing Data on Study Forms 91](#_Toc337159892)

[13.4 Changing Responses on Study Forms 92](#_Toc337159893)

[13.5 Oversight 92](#_Toc337159894)

[13.6 Adherence to Procedures 92](#_Toc337159895)

[13.7 Quality Assurance and Training for Field Trachoma Grading 92](#_Toc337159896)

[13.8 Quality Assurance for Specimen Handling 93](#_Toc337159897)

[13.9 References 94](#_Toc337159898)

[Chapter 14: Data Management and Routine Reports 95](#_Toc337159899)

[14.1 Overview 95](#_Toc337159900)

[14.2 Daily Data Management Activities in Field 96](#_Toc337159901)

[14.3 Data Entry 96](#_Toc337159902)

[14.4 Preparation of Data for Transfer to JHU Data Coordinating Center 98](#_Toc337159903)

[14.5 Data Editing 98](#_Toc337159904)

[14.5.1 Specimen Transfer Data Procedures 99](#_Toc337159905)

[14.6 Generation of Reports 99](#_Toc337159906)

[14.7 Data Storage 101](#_Toc337159907)

[Chapter 15: Adverse Events 102](#_Toc337159908)

[Chapter 16: Data Analyses Plan 103](#_Toc337159909)

[16.1. General Approach 103](#_Toc337159910)

[16.2 Primary/Secondary Outcomes 103](#_Toc337159911)

[16.2.1 Primary Aim/Secondary Aim 1 104](#_Toc337159912)

[16.2.2 Secondary Aim 2 104](#_Toc337159913)

[16.2.3 Secondary Aim 3 105](#_Toc337159914)

[16.2.4 Secondary Aim 4 106](#_Toc337159915)

[16.2.5 Secondary Aim 5 106](#_Toc337159916)

[Chapter 17: Study Organization 107](#_Toc337159917)

[17.1 Study Leadership 107](#_Toc337159918)

[17.2 Executive Committee 107](#_Toc337159919)

[17.2.1 Executive Committee Members & Meetings 108](#_Toc337159920)

[17.3 In-Country Center 108](#_Toc337159921)

[17.4 Study Chairman’s Office 109](#_Toc337159922)

[17.5 Data Coordinating Center 109](#_Toc337159923)

[17.6 Trachoma Grading Center 110](#_Toc337159924)

[17.7 Laboratory Center 111](#_Toc337159925)

[17.8 References 111](#_Toc337159926)

[Chapter 18: Data and Safety Monitoring Committee 113](#_Toc337159927)

[18.1 Charter of Data and Safety Monitoring Committee 113](#_Toc337159928)

[18.2 Terms of Reference 114](#_Toc337159929)

[18.3 DSMC Key Responsibilities 115](#_Toc337159930)

[18.4 DSMC Membership 115](#_Toc337159931)

[18.4.1 DSMC Conflicts of Interest 115](#_Toc337159932)

[18.4.2 Relationships 116](#_Toc337159933)

[18.4.3 DSMC Meetings 116](#_Toc337159934)

[18.4.4 Protocol Changes and Ancillary Studies 117](#_Toc337159935)

[18.4.5 DSMC Recommendations 117](#_Toc337159936)

[Chapter 19: Study Policies 118](#_Toc337159937)

[19.1 Introduction 118](#_Toc337159938)

[19.2 Ancillary studies 118](#_Toc337159939)

[19.3 Editorial Policy 118](#_Toc337159940)

[19.4 Access to Study Information 118](#_Toc337159941)

[Chapter 20: Study Timeline 120](#_Toc337159942)

[MOP Appendix 1: General Study Forms 122](#_Toc337159943)

[MOP Appendix 2: Consent Forms 123](#_Toc337159944)

[MOP Appendix 3: Use of Tablet for data collection 124](#_Toc337159945)

**ASANTE Manual of Procedures: Version Updates**

|  | | |
| --- | --- | --- |
| Version  2  3 | Date  May 2013  February 2014 | Modifications  Rewrites  change procedure for shipping violation, add new child guardian surveillance and treatment procedures |

Chapter

1

# **Chapter 1: Overview**

## 1.1 Overview

This trial is designed to test the impact of two years of a newcomer and travelers treatment strategy on ocular *C. trachomatis* infection in communities in Kongwa District, Tanzania. Forty eight communities have been followed through multiple rounds of annual mass drug administration (MDA) with high coverage as part of a Bill and Melinda Gates Foundation-funded study. While we have observed decreasing infection among these 48 communities, the rate has not declined as rapidly as would be predicted based on the drug manufacturer’s stated efficacy of 95% against *C. trachomatis*. There is also evidence of a slight increase in infection during the time between the annual MDAs.

We hypothesize that the migration of new families from untreated areas into our treated communities, and prolonged travel outside of Kongwa by resident families, are significant sources of re-emergent infection and prevent a more rapid trajectory of decline. This source of infection appears to become more pronounced as the overall community burden of infection decreases and transmission among resident community members decreases.

The 52 communities are now at a low level of infection, 3.3%. We propose a community randomized trial where we re-randomize the 48 communities plus four additional communities as requested by the DSMC (December, 2012 meeting) to annual MDA alone, if warranted by infection status (26 usual practice communities) and to MDA, if warranted by infection status plus surveillance and azithromycin treatment of newcomers and travelers into the community (26 intervention communities). We will call this A Surveillance and Azithromycin Treatment for Newcomers and Travelers Evaluation (ASANTE) trial. If infection in any community has reached 1% or less in the survey preceding the trial, the community will no longer receive azithromycin MDA for the duration of this trial unless infection remerges to 6% or more.

In the 26 communities randomized to the intervention arm, in addition to MDA (if warranted) we will institute a community based surveillance and treatment program. Local Community monitors (CMs) will identify newcomer families, and resident families that have traveled outside the community for at least eight weeks, and offer azithromycin treatment to these families. We define “newcomer” as any family with children under age 10 years that migrates to the community. If they are from a community that has not received MDA in the last year, they will receive treatment. We define “traveler” as any family with children under age 10 years that has returned to the community, having left for eight weeks or more. If they left for another community that has not received MDA in the last year, they will receive treatment. The latter group consists of highly migratory families that have homes in different regions known to be in endemic districts.

To implement this intervention, we plan to use local CMs within the balozi structure of Tanzanian communities to identify and treat newly arrived families. Balozis are clusters of households, typically 10 to 20, that are assigned to elected balozi leaders. This leader is responsible for transmitting information from the community leadership to the residents. Newcomers and travelers who come to the community typically live within a balozi. We will hold neighborhood meetings to nominate a CM who knows the balozi leader and has time to monitor the households for newcomers and travelers according to protocol.

In each community, we plan to monitor infection with *C. trachomatis* and clinical trachoma in a random sample of 100 children ages 1 to 9 years, which we term the sentinel children. They will be selected at random from the latest annual census at baseline, 6, 12, 18, and 24 months. In communities at risk of trachoma re-emergence at 18 months, a random sample of 100 children will be selected from the latest annual census at 30 months. The primary outcome is the proportion of communities in the intervention arm, compared to the control arm, who have a prevalence of infection at 1% or less by 24 months.

Secondary aims are as follows:

1. To determine the proportion of communities in the intervention arm, as compared to the control arm, who have clinical trachoma at less than 5% by 24 months;

2. To determine the trajectory of decline in infection and clinical trachoma in the control communities; and

3. To determine factors that are associated with re-emergence of infection and clinical disease in the communities that have had MDA stopped.

We will take advantage of a supplement to the main trial to investigate the relationship of risk factors, especially indoor cooking fires, to risk of trachoma. Additional secondary aims are:

4. To determine risk factors for clinical trachoma in children in these communities, including exposure to indoor cooking fires; and

5. To determine risk factors, including exposure to indoor cooking fires that are related to scarring trachoma in women in these communities at baseline.

Data will be analyzed for the primary aim at the community level. For the 4^th^ and 5^th^ secondary aims, data will be analyzed at person-level to determine risk factors for active and scarring trachoma.

## 1.2 Research Aims

These specific aims were designed to continue our goal of evaluating strategies for the rapid elimination of trachoma by evaluating the impact of a newcomer and traveler treatment program, which is likely important for communities at low levels of infection in the last stages for trachoma control.

***Primary Aim: To determine the added benefit in reduction of infection with* C. trachomatis *in 26 communities randomized to a newcomer and traveler treatment program in addition to annual mass drug administration versus 26 communities randomized to annual mass drug administration alone.***

We hypothesize that after two years of such a program, twice the number of communities in the intervention arm will have infection at 1% or less compared to the control communities. We will monitor the effect of the added intervention on rates of infection at 6 monthly intervals, with the endpoint being the percentage of communities with a prevalence of infection at 1% or less two years after starting the intervention program. We have 14 communities prior to baseline that have infection at 1% or less, and these will be randomly assigned to intervention and control arms.

***Secondary Aim 1: To determine the added benefit in reduction of clinical trachoma in 26 communities randomized to the newcomer and traveler treatment program in addition to annual MDA versus 26 communities randomized to annual MDA alone.***

We hypothesize that after two years of such a program, twice the number of communities in the intervention arm will have clinical trachoma less than 5% compared to the control communities. We will monitor the effect of the added intervention on rates of trachoma at 6-month intervals, with the endpoint being the percentage of communities with a prevalence of clinical trachoma at less than 5% two years after starting the intervention program.

***Secondary Aim 2: To determine the trajectory of decline in infection with* C. trachomatis *and clinical trachoma in communities randomized to usual care with two additional rounds of mass drug administration with azithromycin.***

Infection has declined to 3.3% overall and we have a unique opportunity to continue to follow the decline in the usual care arm (26 of our 52 communities) associated with just MDA. We will calculate the trajectory of decline with two additional rounds of mass treatment and the effect of stopping MDA when the target is reached. Communities will have MDA stopped if infection is 1% or less, or TF is less than 5%, as based on our sentinel samples.

***Secondary Aim 3: To determine the risk of re-emergent infection in communities that have reached endpoint, where the pressure of mass antibiotic treatment has been removed.***

The re-emergence of infection and clinical disease has not been well characterized in a setting such as ours, where the starting prevalence was high and the entire surrounding district of non-study communities was also being treated. In The Gambia, infection and trachoma rates are very low; after MDA, infection re-emerged in communities where the whole district was treated, but was not sustained and disappeared [1]. In Ethiopia, after infection in communities was reduced to 1%, MDA was stopped and re-emergence was a significant problem, but these communities were in a hyperendemic area where the surrounding communities were not being treated [2]. In Kongwa district, MDA is now confined to our study villages and a single sub district that has trachoma more than 10%. As we follow our 52 study communities, we plan to stop MDA in those communities who have achieved our pre-specified goal, amended in February 2014 at the DSMC meeting as follows: infection estimated at 1% or less (with an upper confidence interval of less than 6%) or TF less than 5%. We have the opportunity to monitor the communities in the intervention and control groups for re-emergence of infection and chart the likely course, or absence, of re-emergence up to a two and a half-year period. We hypothesize that re-emergence will be greater in the control communities where MDA was stopped, compared to the intervention communities where MDA was stopped but the newcomer and traveler program was instituted.

***Secondary Aim 4: To determine risk factors for clinical disease in children in these communities, including exposure to indoor cooking fires.***

Previous studies have linked sleeping by a cooking fire to trachoma, but other risk factors were not accounted for and the assessment of exposure was simple. We are taking advantage of a supplement to our trial by adding a risk factor questionnaire to our baseline surveys, which assesses exposure to an indoor cooking fire. We will examine the cross sectional relationship of this exposure to severity of trachoma in children we are already examining as part of the sentinel sample in each community.

***Secondary Aim 5: To determine risk factors, including exposure to indoor cooking fires, that are related to scarring trachoma in women in these communities.***

Scarring from trachoma is 4 times as common in females as in males. In part, this is thought to be due to higher rates of exposure to infection, but other environmental factors are also likely. We hypothesize that more severe scarring in women in these communities is attributable to exposure to indoor cooking fires. We will be assessing exposure to indoor cooking fires as part of the household survey, and plan to add a random sample of 100 women in each community, determine the presence and severity of scarring, and model the cross sectional associations with exposure to cooking fire.

## 1.3 Overall Study Design

The ASANTE trial uses a community-randomized design, where the 52 communities will be randomized to one of two treatment arms: 1) control, which is MDA unless the community is eligible to be “graduated” because infection prevalence is ≤1% or TF is <5% in the sentinel sample of 1-9 year olds, or 2) intervention, which in addition to MDA (or “graduation”) includes a program of surveillance and treatment of newcomers and travelers in the community.

At the start of this trial we will have infection prevalence data for 48 of the 52 communities. These data will enable us to stratify randomization of communities by level of infection prior to baseline, as well as by number of previous rounds of MDA. Using constrained randomization, we will randomize the 24 communities to each arm, using the allocations that meet the balance criteria, and randomize the additional 4 communities by simple random sample.

Each of the 52 communities will have a census carried out prior to starting the trial, and an updated census again prior to the 12- and 24-month surveys. One hundred sentinel children ages 1 to 9 years will be randomly selected from each of the communities based on the latest census for surveys of trachoma and infection with *C. trachomatis* at baseline, 6, 12, 18, and 24 months. Based on the recommendation of the DSMC in March 2015, communities at risk of trachoma re-emergence at the time of the 18 month survey will also participate in a 30 month survey. The surveillance and treatment of newcomers and travelers program will continue 6 months beyond the time of the 24 month survey in intervention communities only.

One hundred females aged 15 and older will also be randomly selected from each of the original 48 communities to assess scarring at baseline, to fulfill secondary aim 5.

In both arms, communities with estimated infection rates ≤ 1% or TF rates<5% in sentinel children will be “graduated” from further MDA and followed for up to 30 months to look for evidence of re-emergent infection and disease.

The primary outcome is the proportion of communities in each arm at the end of two years with infection prevalence at 1% or below.

## 1.4 References

1. Burton MJ, Holland MJ, Makalo P, et al. Re-emergence of Chlamydia trachomatis infection after mass antibiotic treatment of a trachoma-endemic Gambian community: a longitudinal study. Lancet 2005;365:1321-8.
2. Lakew T, House J, Hong KC, et al. Reduction and return of infectious trachoma in severely affected communities in ethiopia. PLoSNeglTropDis 2009;3:e376.

Chapter

2

# **Chapter 2: Background and Significance**

## 2.1 Background

The leading infectious cause of blindness worldwide, trachoma is caused by repeated episodes of an ocular infection with *Chlamydia trachomatis* [1]. Once endemic in most countries, trachoma has largely disappeared from Europe and the Americas. Trachoma continues to be endemic in many of the poorest and most remote areas of Africa, Asia, and the Middle East. Communities with trachoma are often those with the fewest resources to take on health issues, and trachoma strikes the most vulnerable members of those communities, women and children. Trachoma affects an estimated 40 million people and an estimated 7.6 million are blind or severely visually impaired due to trachoma [1]. The total productivity loss from visual loss due to trachoma is estimated at $5.3 billion (2003 US dollars), and health economists suggest an even greater burden when including disability due to the chronic sequelae of trachoma even prior to visual impairment [2].

In endemic communities, trachoma is a chronic disease. The community pool of active inflammatory disease trachoma and infection resides in the children who may have persistent signs of active trachoma as a result of repeated infections. Multiple infections and/or prolonged, severe infection are followed by evidence of scarring of the conjunctiva. Even in late childhood and early adult hood, the scarring may be prominent and obscure evidence of active disease, although in some cases of scarring without evidence of active disease there is laboratory evidence of *C. trachomatis* infection [3]. The scarring can be significant enough to cause entropion and trichiasis, or inturned eyelashes which, if not surgically corrected, can lead to corneal damage and loss of sight. There is currently no treatment option for corneal opacification, or any intervention to halt the scarring process. The most effective method for prevention of these late manifestations of trachoma is to halt the active disease in children.

The World Health Organization (WHO) has recommended a multifaceted “SAFE” strategy for trachoma control programs [4]. This approach includes Surgery for trichiasis cases, and A, F, and E to control active trachoma: Antibiotics to treat the community pool of infection, Face washing and Environmental change to sustain reduction in transmission. There is epidemiological evidence to support each component of the SAFE strategy, which must be implemented on a community-wide basis. Azithromycin is currently the drug of choice for MDA. It is effective as a single, oral dose of 20mg/kg up to 1 gm. The concentration in tissues is greater than the concentration in the plasma, and this plus the long half-life make it an ideal antibiotic for an intracellular organism with slow growth, like *C. trachomatis* [5]. It is safe for use in children, and has been used to treat infections in children as young as one month. The cost of azithromycin treatment originally put it out of reach for trachoma control programs, but the manufacturer instituted a generous donation program that makes the drug free of charge to countries who apply for it for use in trachoma control.

The WHO recommends mass treatment with antibiotics, preferably azithromycin, when the prevalence of follicular trachoma (TF) is more than 10% in children ages 1 to 9 years at the district level [6]. Mass treatment changes to targeted treatment when prevalence is between 5% and 10%. The Ultimate Intervention Goal set by WHO for trachoma is a prevalence of TF less than 5% in children ages 1 to 9 years. At this level of disease, trachoma is not considered to be a blinding condition and antibiotic treatment can be stopped. Clinical disease is used as the criteria for initiating and stopping treatment, although this is not ideal because the clinical signs are not a good indicator of infection [7], which is the real target of antibiotic treatment. However, detection of infection is possible only in research settings at present and our study enables us to evaluate both infection and clinical disease outcomes. There are no criteria based on community **infection** status for initiating or stopping MDA. We chose the criteria of 1% or less based on two factors: first, data from The Gambia suggested that when infection was low, around 1%, even if infection was re- introduced it was not sustainable [8]. Second, we wished to ensure with 95% confidence that infection was no higher than 6%, the upper bound of precision for declaring MDA to be stopped based on **clinical** trachoma criteria. In order to ensure this in each community, we would allow no more than one infection case per 100 sentinel children sampled. At the January 2014 DSMC meeting the stopping rule was amended to also add no more than 4 cases per 100 children sampled with TF.

Mass treatment with azithromycin has a strong effect on decreasing Chlamydial load following mass treatment [9-10]; however, the hope that 1-2 rounds of annual MDA would be sufficient to eliminate trachoma or infection in most communities has not been realized, and the reasons why infection remains after MDA are varied. WHO guidelines suggest that mass treatment be provided for all communities in a district for at least three years, and a recent WHO Global Scientific Meeting concluded that when the starting prevalence of TF in a district is high, for example 30%, at least five years of annual mass antibiotic administration will be needed before any expectation of significant change [11].

Our previous NEI-funded study modeled data after seven years of program-administered mass treatment with moderate coverage (<75%), and projected that ten years of MDA would be needed under conditions of 75% coverage to achieve rates of follicular trachoma less than 5% [12]. However, we have observed in our current project that with high coverage, consistently above 80% in each round, infection has declined to 3.3% and trachoma to 9%, suggesting that we may achieve the goal in most communities with less than ten rounds. These additional rounds of MDA and follow-up in the control arm are needed to allow us to determine this trajectory.

But the source of infection following mass treatment is unknown. Non-compliance with treatment was originally a predictor of infection and disease at follow up in studies in three different settings [13]. However, even with very high compliance, over 95% of children, infection with *C. trachomatis* at 6 months post-mass treatment was 42% of the pre-treatment levels. There were too few untreated children to suggest non-compliance was the issue [14].

Some have argued these young children are one source of re-emergent infection because they are treated (likely unevenly) with topical tetracycline. Infants less than 6 months of age are not eligible for azithromycin, and may have very high Chlamydial burdens [15]. However, our study in Tanzania which addressed this issue found that infections were few in this age group and that households with infants were not at increased risk of infection following mass treatment [16].

Persons who had infection prior to treatment, and who had the greatest infectious burden prior to treatment, are also at risk of infection post-treatment [10,17]. Resistance of *Chlamydia* to azithromycin has not been reported, and it was hypothesized that infection may be due to an insufficient dose of azithromycin for those with heavy infection. However, increasing the dose from 20 to 30 mg/kg in children with infection in a clinical trial did not result in significantly less infection 6 weeks post treatment [18]. In that study, where children (rather than communities) were followed over time, infection decreased from 69% at baseline to 12% at 6 weeks post treatment, an 82% reduction. In a community-based trial where children at baseline were followed to six months and 95% of children were observed receiving treatments, infection declined from 24% at baseline to 10% at 6 months, resulting in only a 42% reduction [14]. Children with infection were not preferentially treated, so it is likely that the period immediately after MDA to 6 months allowed residual and newly introduced infections to spread. Even within hyperendemic areas, trachoma clusters both at the neighborhood and at the household level [19-22], as might be expected from an infectious disease where transmission can occur by sharing clothes, towels, or sleeping quarters. There is evidence that after MDA, children remaining with infection can spread it within the household by six months, and across to neighboring households within one year [23].

Another source of infection is the reintroduction of *C. trachomatis* with in-migration of individuals who can bring in infection and from returning community members who acquire infection outside. It was the primary explanation for re-emergence of infection in two communities in The Gambia [24] and in Tanzania; children of families who are new to a previously mass treated community, having come from outside, are five times more likely to have infection compared to children whose families were present during MDA (paper submitted). Travel by families outside the village where interactions occur that might re-infect the children is another source of re-emergent infection [25]. In Ethiopia, travelers were more likely to have infection post- MDA than non-travelers. The issue of in-migration, or travelers who bring back trachoma, has not been addressed in trachoma control programs. In part, the thought is that these persons will be treated during the annual MDA. However, once infection rates in the villages are lowered, these sources become a significant component of the residual infection and a possible reason why the trajectory of decline is less steep than expected. If these sources are in fact shown to impede progress towards elimination, then a local strategy that addresses travelers and a nationwide strategy that addresses intra- and international migration will be needed.

Another key issue is prevention of re-emergence once antibiotic pressure is removed. The history of trachoma control programs is littered with re-emergent disease in countries once control had been stopped, such as in Ethiopia, Sudan, Morocco, and Australia [26]. Re-emergence of infection with cessation of MDA does occur, and has been reported primarily from hyperendemic communities [17,24,27]. A pattern of resurgence of infection in between annual MDA emerged, though not to pre-treatment levels, with continued re-emergence after cessation in Tanzania [28]. Return of infection 18 months following cessation of treatment in Ethiopian hyperendemic communities has also been reported [27]. However, where trachoma rates are very low (The Gambia), infection following a single MDA was largely gone over the follow-up period of 17 months, except in two villages where residents had extensive contact with other, untreated villages in Senegal [24]. However, even these imported infections did not lead to re-emergence to the level of baseline, as infection declined without treatment in these two villages from 30% at the time of re-infection to 11% at 17 months and 0% at five years. Thus, at low levels of infection, the question remains if travelers and newcomers to the community, if infected, can be a source of re-emergent infection in communities and thus pose a risk of failure of the program to sustain trachoma elimination.

Finally, we have an opportunity to add a risk factor survey at baseline for infection and disease, and scarring in women, as part of a supplement received for this trial. We have previously shown using a simple question on sleeping next to a cooking fire, an association with exposure and active trachoma in children [29] and trichiasis in women [30]. However, this finding needs to be confirmed with a more rigorous approach to the questions asked, and to confirm that it is related to scarring in women, prior to trichiasis. If we find there is a relationship, then avoiding indoor cooking fires can be another environmental improvement in the armamentarium of trachoma control programs, and aligns with national policies for improving indoor air pollution.

In summary, trachoma continues to be a significant public health problem worldwide. As more and more countries achieve a relatively low rate of infection and disease, the remaining sources of re-emergent infection assume a prominent role and impede steady progress towards elimination. The significance of our project lies in the fact that this is the first research study to determine the benefit of a newcomer and traveler surveillance and treatment program on the trajectory of decline in infection and trachoma, and determine the importance of such a program on interrupting re-emergent infection in communities where MDA has been stopped. We take advantage of the trial to incorporate a timely study of another environmental risk factor for active trachoma in children, and possibly a factor for scarring in women.

## 2.2 Preliminary Studies

In 2007, we undertook a trial that was in two parts. A total of 32 communities in Kongwa, Tanzania were randomized in a 2X2 factorial design to test two hypotheses, the first that with very high coverage, communities could cease mass treatment before the third round of MDA. [29]. The baseline and interim ancillary findings have been published and the final report is due out in 2012 [14,31-34]. The trial was also designed to determine the cost effectiveness of stopping mass treatment early in communities where infection fell below 1% (intervention) versus continuing with mass treatment for all 3 rounds regardless of infection status. The World Health Organization does not have a guideline for stopping MDA based on infection prevalence, only on trachoma prevalence, which must be less than 5%.

We randomized 32 communities with baseline prevalence of trachoma above 20% (Group 1 communities). After 1.5 years, we received additional funds to further randomize 16 communities with average baseline prevalence between 10% and 20% (Group 2 communities).

The two key findings from the trial were: first, in both coverage arms, infection and disease declined with no differences at final follow up in infection prevalence in children in communities with MDA coverage between 80% -89% compared to communities with coverage above 90%. This finding is important for programs because 80% is a realistic target and less expensive to implement. Second, we were unable to stop any communities in either arm of the trial before 3 rounds of MDA. This finding further supports the WHO guideline that at least three rounds of MDA will be needed in communities in districts where the prevalence is above 10%. While we did not stop MDA in any of the communities during the trial, after subsequent rounds of MDA we have now stopped MDA in 14 of the 48 communities.

In preparation for the ASANTE trial, we have been conducting interim surveys, for which we enrolled a random sample of children ages under ten years of age. The field work for the ASANTE trial will start in January 2013 and end in December 2015 with the completion of the final survey. Note that, as described further in the section on randomization (see Section 3.1 Randomization of Communities), we will have an equal distribution of Group 1 and Group 2 communities in the intervention and control arm of this proposed trial, which are now at similar levels of infection prevalence.

Our 24-month data suggest that each year an average of 4-5% of the families in the community are new arrivals. In addition, an estimated 15% of children reside in households in close proximity to traveling households (Table 2.1). Children in the newcomer families and children who resided in neighborhoods with travelers were significantly more likely to have infection at 24 months compared to children who were in families that were resident and not in neighborhoods with travelers. At 24 months, where infection overall was at 8%, at least 20% of infection was attributable to residing in neighborhoods with travelers or to newcomers to the community. This estimate is likely conservative for two reasons: first, we could only estimate the travelers in each community based on retrospective reports of the census team and not on actual travel (which would be detected prospectively in the proposed trial). Second, this estimate was made at 24 months and we have since completed two more rounds of MDA. The attributable fraction is likely greater than 20% as we decrease the infection rates in the community residents. When the communities are hyperendemic, newcomers and travelers are factors that are part of a larger picture of multiple routes of transmission and sources of infection.

**Table 2.1: Infection rate in Children following 24 month survey after 3 rounds of MDA in 32 villages***

| Place of residence | N | % infection | p-value*** |
| --- | --- | --- | --- |
| Resident household in neighborhood with low % of travelers | 4447 | 7.3 | 0.005 |
| Resident household in neighborhood with high** % of travelers | 769 | 10.0 |  |
| New household in neighborhood with low % of travelers | 198 | 14.1 |  |
| New household in neighborhood with high* % of travelers | 39 | 18.0 |  |

*In 4 randomly selected communities, all children were examined, and in the rest 100 sentinel children were examined. 24 month survey data includes a census update and thus newcomers were identifiable.

** Neighborhoods with more than 6% of residents traveling outside the community for most of the year

*** comparing resident households from neighborhoods with low proportion of travelers with the other 3 groups, from a random effects model accounting for clustering at village and household level.

The average infection rate after the 3^rd^ round of MDA is 3.5% in the 32 Group 1 communities and 3% in the 16 Group 2 communities. After three rounds of MDA , 10 communities have infection prevalence of 1% or less and currently, 14 communities have infection at 1% or less . These data show that we may expect roughly halving of infection with each round of MDA. Thus, we expect a further decline in infection to 2% after the 4^th^ round and in group 2, we may reasonably expect infection to decline to 1.5% after the 3rd round of MDA. As we cease mass treatment in these communities we expect to have between 1 to 2.5 years to observe them for re-emergent infection. As discussed, we expect that the newcomer treatment program will prevent re-emergence as well as hasten the decline of infection in these communities.

## 2.3 Impact

This trial is a unique opportunity to investigate the impact of a surveillance and treatment program for families who may be importing infection into communities where infection is close to eliminated. No other research program has the ability to undertake such an innovative program at such a critical juncture in the trajectory of elimination as we have within the 48 communities. The outcome of the trial will have an impact in several areas. First, if the intervention is successful in increasing the proportion of communities that can be graduated, then this finding will guide national programs which will need to develop such a surveillance program to achieve rapid elimination. Even if the program has no significant impact, such a finding is important to the understanding that when infection is low, re-introduction is unlikely to be sustained or increase re-emergent infection. Second, we are planning to chart the trajectory of decline in infection with further rounds of MDA with high coverage, and “graduating “ communities whose infection rates are less than or equal to 1% or whose TF rates are <5%. Such empirical data are critical for national programs in planning for the length of time MDA is needed when their starting prevalence resemble our communities. Finally, scant attention has been paid to the issue of re-emergence of infection, and risk factors for re-emergence. We will have the opportunity to describe re-emergence in these communities that have ceased MDA, and characterize under what conditions (like absence of the ASANTE program) that re-emergence is observed. Finally, we will have a better understanding of the role of exposure to indoor cooking fires on active trachoma in children. If it is also related to the severity of scarring in women, then this will be the first modifiable environmental risk factor that could be addressed to lower risk in persons with scarring.

## 2.4 References

1. Mariotti S, Pascolini D, Rose-nussbaumer J. Trachoma:Global Magnitude of a preventable cause of blindness. Br J Ophthalmol 2009;93:563-8.
2. Frick KD, Hanson CL, Jacobson GA. Global burden of trachoma and economics of the disease. AmJTropMedHyg 2003;69:1-10.
3. Munoz B, Bobo L, Mkocha H, Lynch M, Hsieh YH, West S. Incidence of trichiasis in a cohort of women with and without scarring. Int J Epidemiol 1999;28:1167-71.
4. Global elimination of blinding trachoma. 1998. (Accessed WHA 51.11 at http://www.who.int/ncd/vision2020_actionplan/documents/WHA51.11.pdf.
5. Girard AE GD, English AR, Gootz TD, Cimochowski CR, Faiella JA, Haskell SL, Retsema JA. Pharmacokinetic and in vivo studies with azithromycin (CP-62,993), a new macrolide with an extended half-life and excellent tissue distribution. Antimicrob Agents Chemother 1987;12:1948-54.
6. WHO_working_group. Report of the eighth meeting of the WHO Alliance for the Global Elimination of blinding trachoma. Geneva: World Health Organization WHO/PBD/GET/04.2; 2004.
7. Keenan JD LT, Alemayehu W, Melese M, House JI, Acharya NR, Porco TC, Gaynor BD, Lietman TM. Slow resolution of clinically active trachoma following successful mass antibiotic treatments. Arch Ophthalmol 2011;129:512-3.
8. Burton MJ, Holland MJ, Makalo P, et al. Profound and sustained reduction in *Chlamydia trachomatis* in the Gambia: a five-year longitudinal study of trachoma endemic communities. PLoS Negl Trop Dis. 2010;4:e835.
9. Solomon AW, Holland MJ, Alexander ND, et al. Mass treatment with single-dose azithromycin for trachoma. NEnglJMed 2004;351:1962-71.
10. West ES, Munoz B, Mkocha H, et al. Mass treatment and the effect on the load of Chlamydia trachomatis infection in a trachoma-hyperendemic community. Invest OphthalmolVisSci 2005;46:83-7.
11. World_Health Organization. Third Global Scientific Meeting on Trachoma Baltimore Johns Hopkins University 2010 July 2011.
12. West SK, Munoz B, Mkocha H, Gaydos C, Quinn T. Number of Years of Annual Mass Treatment with Azithromycin Needed to Control Trachoma in Hyper-endemic Communities in Tanzania. J Infect Dis 2009;204:268-73.
13. Schachter J, West SK, Mabey D, et al. Azithromycin in control of trachoma. Lancet 1999;354:630-5.
14. Cajas-Monson LC, Mkocha H, Munoz B, Quinn TC, Gaydos CA, West SK. Risk factors for ocular infection with Chlamydia trachomatis in children 6 months following mass treatment in Tanzania. PLoS Negl Trop Dis 2011;5:e978.
15. Solomon AW, Holland MJ, Burton MJ, et al. Strategies for control of trachoma: observational study with quantitative PCR. Lancet 2003;362:198-204.
16. West SK SD, Mkocha H, Munoz B, Gaydos C, Quinn TC. Do Infants Increase The Risk Of Re-emergent Infection In Households After Mass Drug Administration For Trachoma? Invest Ophthalmol Vis Sci 2011.
17. West SK, Munoz B, Mkocha H, et al. Infection with Chlamydia trachomatis after mass treatment of a trachoma hyperendemic community in Tanzania: a longitudinal study. Lancet 2005;366:1296-300.
18. Campbell JP, Mkocha H, Munoz B, West SK. Randomized trial of high dose azithromycin compared to standard dosing for children with severe trachoma in Tanzania. Ophthalmic Epidemiol 2009;16:175-80.
19. West SK, Munoz B, Turner VM, Mmbaga BBO, Taylor HR. The epidemiology of trachoma in Central Tanzania. Int J Epidemiol 1991;20:1088-92.
20. Katz J, Zeger S, Tielsch L. Village and household clustering of xerophthalmia and trachoma. Int J Epidemiol 1988;17:865-9.
21. Blake IM, Burton MJ, Bailey RL, et al. Estimating Household and Community Transmission of Ocular Chlamydia trachomatis. PLoSNeglTropDis 2009;3:e401.
22. Bailey R, Osmond C, Mabey DC, Whittle HC, Ward ME. Analysis of the household distribution of trachoma in a Gambian village using a Monte Carlo simulation procedure. Int J Epidemiol 1989;18:944-51.
23. Broman AT, Shum K, Munoz B, Duncan DD, West SK. Spatial clustering of ocular chlamydial infection over time following treatment, among households in a village in Tanzania. Invest OphthalmolVisSci 2006;47:99-104.
24. Burton MJ, Holland MJ, Makalo P, et al. Re-emergence of Chlamydia trachomatis infection after mass antibiotic treatment of a trachoma-endemic Gambian community: a longitudinal study. Lancet 2005;365:1321-8.
25. Shah NA, House J, Lakew T, et al. Travel and implications for the elimination of trachoma in ethiopia. Ophthalmic Epidemiol 2010;17:113-7.
26. Taylor HR. Trachoma; a blinding scourge from the bronze age to the twenty first century. 1 ed. Melbourne: Center for Eye Research Australia; 2008.
27. Lakew T, House J, Hong KC, et al. Reduction and return of infectious trachoma in severely affected communities in ethiopia. PLoSNeglTropDis 2009;3:e376.
28. West SK, Munoz B, Mkocha H, Gaydos C, Quinn T. Trachoma and ocular Chlamydia trachomatis were not eliminated three years after two rounds of mass treatment in a trachoma hyperendemic village. Invest OphthalmolVisSci 2007;48:1492-7.
29. Turner VM, West SK, Muñoz B, Katala SJ, Taylor HR, Halsey N, Mmbaga BB. Risk factors for trichiasis in women in Kongwa, Tanzania: a case-control study. Int J Epidemiol. 1993;22(2):341-7.
30. Taylor HR, West SK, Mmbaga BB, et al. Hygiene factors and increased risk of trachoma in central Tanzania. ArchOphthalmol 1989;107:1821-5.
31. Stare D, HardingEsch, E., Munoz, B., Bailey, R., Mabey, D., Holland, M., Gaydos, C., West, S.K. Design and Baseline Data of a Randomized Trial to Evaluate Coverage and Frequency of Mass Treatment with Azithromycin: Partnership for Rapid Elimination of Trachoma. . Ophthalmic Epidemiol 2011;14:5-11.
32. Harding-Esch EM, Edwards T, Sillah A, et al. Active trachoma and ocular Chlamydia trachomatis infection in two Gambian regions: on course for elimination by 2020? PLoS Negl Trop Dis 2009;3:e573.
33. Ssemanda EN, Munoz B, Harding-Esch EM, et al. Mass treatment with azithromycin for trachoma control: participation clusters in households. PLoS Negl Trop Dis 2010;4.
34. Rog M, Swenor B, Cajas-Monson LC, et al. A cross-sectional survey of water and clean faces in trachoma endemic communities in Tanzania. BMC Public Health 2011;11:495.

Chapter

3

# **Chapter 3: Randomization and Sample Size**

## 3.1 Randomization of Communities

The ASANTE Trial is a superiority trial. The unit of randomization is the community. We will have data on 48-month/30-month surveys by the start of this trial. These data will enable us to stratify communities in PRET by level of infection at the outset, whether they have stopped MDA, and by being a Group 1 or Group 2 community (see Section 2.2 Preliminary Studies). We plan to use a constrained randomization approach as suggested by Moulton [1] for this community randomized design. This approach reduces the likelihood of a bad randomization outcome by balancing on Group (16 communities in each arm from Group 1 and 8 communities in each arm from Group 2) and on infection rates as of the last survey (those communities at or below 1% with MDA stopped and those communities above 1%, where current data suggest a total of 14 communities will be at 1% or below) as covariates. We will allocate 24 communities from PRET to each arm, using the allocations that meet the balance criteria, and employing a SAS Macro developed by Ms. Munoz for this purpose. For the four additional communities added after the DSMC meeting, we will randomize them to treatment arms using a simple table of random numbers.

We expect 14 of these communities at the start of the trial to be no longer eligible for mass drug administration because of infection rates less than 1% following the previous round of MDA, and these will be followed for re-emergent infection. Ms. Munoz will do the random assignment of communities, and provide the information to Mr. Mkocha, director at KTP, who will implement the strategy in the field. The laboratory personnel, who assess infection status, will be masked to community assignment. The survey team will also be masked to community assignment.

## 3.2 Sample Size

### 3.2.1 Communities

This superiority trial has a sample size of 52 communities. Power calculations for this trial are based on our primary outcome variable, proportion of communities with prevalence of *C.trachomatis* of 1% or below 24 months post randomization. Because of the additional treatment for newcomers in the communities assigned to the intervention, we expect to have better outcomes in this group with no reason to suppose that a newcomer and traveler treatment program would result in worse infection. However to be conservative, we used a two-sided Fisher’s exact test and a significance level of α=0.05 to estimate the power.

We make the following assumptions: We expect an average starting prevalence of infection of 3% and expect to observe a decrease in the infection rate by half after one year. Prior to the beginning of the trial we expect to have 8 to 9 communities in each treatment arm with infection prevalence at 1% or below or prevalence of TF below 5%, for which we will stop MDA at baseline (Figure 3.1).

In the control arm, of the 8 communities with infection prevalence at or below 1% or TF prevalence below 5%, we expect infection prevalence to increase to 6% or more in 3 communities after one year and in the remaining 5 communities after two years. Of the 18 communities in the control arm that are continuing MDA, we would expect half of the communities (9) to achieve prevalence of infection of 1% or below or prevalence of TF below 5% after one year. These 9 communities would then stop MDA, and we expect to observe prevalence of infection greater than 1% 5 of these communities after two years. The other 9 communities plus the 3 where initiation of MDA was indicated would have MDA at one year and we would expect to observe prevalence of infection above 1% in 75% (8/12) of these communities after two years. Overall we expect 18 communities in the control arm with infection prevalence above 1% and in at least 5 of them the infection prevalence will be ≥6%.

In the intervention arm, of the 9 communities with infection prevalence at or below 1% or prevalence of TF below 5%, we expect no re-emergence after one year. Of the remaining 17 communities in the intervention arm that are continuing MDA, we expect 11 communities to achieve prevalence of infection of 1% or below or TF prevalence below 5% after one year, for a total of 20 communities not receiving MDA at 1 year, of these 20 we expect 75% (15) to be below 1% infection after two years due to the intervention. The other 8 communities would continue MDA and we expect that at least 4 will achieve prevalence of infection of 1% or below after two years. Overall we expect at 7 communities in the control arm with infection prevalence above 1% and in none the infection prevalence will be ≥6%.

Thus, after two years, a total of 19 communities will be at prevalence of infection 1% or below in the intervention arm, as compared to 8 communities in the control arm. We would expect re-emergence in the initial 8 communities that have stopped treatment in the control arm, and if the infection rate we observe is around 11% in newcomers and travelers, as observed in Table 1 in Section 2.2 Preliminary Studies, then that would be sufficient to push the prevalence estimate of all 8 of the communities back above 1% by two years. In addition, we would expect re-emergence within the 10 newly stopped communities after the first MDA of approximately 4 communities by two years. Thus, the net gain in communities with infection prevalence at 1% or below would be 11 in the intervention group (Figure 3.1).

Figure 3.1

Expectation of infection prevalence at 1% or below in Intervention and Control Arms, with Stopping MDA at 1% infection or less that 5% TF, current infection rates at 3%, and re-emergence due to infection in newcomers and travelers.

In the intervention arm, we would expect no need to restart treatment 9 communities stopped at baseline and in the 11 communities stopped at one year. By two years, we expect that in 15/20 communities would be at 1% or below. Given these assumptions, we examined the power for an ample range of differences in the outcome, starting with a higher proportion of communities stopping MDA (17/26 communities being at or below 1% at 24 months) to a lower proportion of communities stopping MDA (7/26 communities at 1% or below at 24 months) in the control group. Table 3.1 shows the power to detect differences in the proportion of communities at 1% or below between the control and intervention groups.

The shaded rows on Table 3.1 indicate our likely outcome, with 8 out the 26 communities or p_0_=0. 308 in the control group having prevalence of infection of 1% or less at 24 months. We will have sufficient power to detect differences if the number of communities at or below 1% in the intervention group is 2.4 times or more (i.e. p_1_≥0.731 or p_1_/p_0_≥2.38). A similar reasoning will apply for our secondary outcome, proportion of communities with prevalence of follicular trachoma at or below 5%. At present, we expect 3 communities in each arm to have follicular trachoma at 5% or below at the start of the trial, and at most an increase to 9 communities in the control arm. We have ample power if we find 20 or more communities in the intervention arm with TF below 5%. Given our trajectory for decline in infection and trachoma to date, and our expectation that the intervention will amplify that trajectory because of the higher rate of infection in newcomers and travelers, we feel the sample of 52 communities is sufficient to provide a meaningful difference.

**Table 3.1. Power to detect significant differences in the proportion of communities below the threshold for infection/follicular trachoma between the two groups of 26 communities each, using two-sided Fisher’s exact test and a significance level of 0.05**

| Proportion with prevalence below or at threshold at 24 months post randomization | | Ratio (p_1_/p_0_) | Power |
| --- | --- | --- | --- |
| Control Group (p_0_) | Intervention Group (p_1_) |  |  |
| 17/26 (65.4%) | 24/26 (92.3%) | 1.41 | 0.60 |
|  | 25/26 (96.2%) | 1.41 | **0.80** |
| 16/26 (61.5%) | 24/26 (92.3%) | 1.50 | 0.68 |
|  | 25/26 (96.2%) | 1.56 | **0.86** |
| 15/26 (57.7%) | 23/26 (88.5%) | 1.53 | 0.62 |
|  | 24/26 (92.3%) | 1.60 | 0.78 |
|  | 25/26 (96.2%) | 1.67 | **0.92** |
| 14/26 (53.4%) | 23/26 (88.5%) | 1.64 | 0.72 |
|  | 24/26 (92.3%) | 1.71 | **0.85** |
| 13/26 (50.0%) | 22/26 (84.6%) | 1.69 | 0.69 |
|  | 23/26 (88.5%) | 1.77 | **0.81** |
| 12/26 (46.2%) | 21/26 (80.8%) | 1.75 | 0.67 |
|  | 22/26 (84.6%) | 1.83 | 0.79 |
|  | 23/26 (88.5%) | 1.92 | **0.88** |
| 11/26 (42.3%) | 21/26 (80.8%) | 1.91 | 0.77 |
|  | 22/26 (84.6%) | 2.00 | **0.94** |
| 10/26 (38.5%) | 20/26 (76.9%) | 2.00 | 0.75 |
|  | 21/26 (80.8%) | 2.10 | **0.85** |
| 9/26 (34.6%) | 19/26 (73.1%) | 2.11 | 0.73 |
|  | 20/26 (76.0%) | 2.22 | **0.83** |
| 8/26 (30.8%) | 18/26 (73.1%) | 2.25 | 0.72 |
|  | 19/26 (73.1%) | 2.38 | **0.81** |
| 7/26 (26.9%) | 17/26 (65.4%) | 2.43 | 0.73 |
|  | 18/26 (69.2%) | 2.57 | **0.81** |

Based on this estimate, 8 out the 26 communities or p_0_=0. 308 in the control group having prevalence of infection of 1% or less at 24 months, we will have sufficient power (>80%) to detect differences if the number of communities at or below 1% in the intervention group is 19 or more (i.e. p_1_≥0.731 or p_1_/p_0_≥2.38).

### 3.2.2 Children

We chose a sample size of 100 children per community because we need a sufficient number of children to determine with precision if infection has fallen to 1% with an upper confidence bound of 6%. At the same time, we balanced concerns for costs and survey logistic difficulties if this methodology is to be used by country programs to monitor infection or disease. With 100 children in the sample, if one child has infection, the upper 95% confidence limit of the estimated prevalence is less than 6% (5.4%).

### 3.2.3 Women

We calculated the power to detect differences in scarring in simple proportions of exposed and unexposed women, assuming a two-sided significance level α=0.05 and an intra-class correlation coefficient ρ=0.08 for scarring among women residing in the same community. Based on a recent study carried out in a community located in the same district, the expected prevalence of trachomatous scars in women would be between 20 and 30%. Assuming a prevalence of 20% in the unexposed group, we chose a sample size of 100 women per community to have a sufficient number of women to have 80% power to detect odds ratios above 1.58 if the proportion of women exposed to a cooking fire inside their homes is in the 50% to 70% range.

## 3.3 References

1. Moulton LH. Covariate-based constrained randomization of group-randomized trials. Clinical Trials 2004;1:297-305.

Chapter

4

# **Chapter 4: Intervention**

## 4.1 Overview

The intervention for this trial is a community-based surveillance program that identifies families that are newcomers and travelers in the community, and offers them immediate treatment with azithromycin if they are eligible. We have hypothesized that our intervention will result in a greater proportion of the intervention communities with infection rate at 1% or below compared to communities with no newcomer and traveler intervention program.

We define “newcomers” as newly arrived families with children under ten who have moved into a new house in the community, or into an existing household and plan to reside in the community for at least one month. If they have moved from a community that has not had an MDA in the last year they are eligible for treatment. . We define “travelers” as resident families with children under ten, previously in the census, who left the community for more than two months. If they left for an area that has not received MDA in the past year and at least one child has returned then they are eligible for treatment. The latter group comprises highly migratory families that have homes in different regions known to be in endemic districts. We chose two months for travel, because the travel away should be of long enough duration to acquire infection by residing in another community.

## 4.2 Newcomer and Traveler Treatment Intervention

In the 26 communities randomized to the intervention arm, the study plan will build a surveillance system for families that meet “newcomer” or “traveler” status and provide interim treatment with azithromycin to these families. To implement this intervention, we plan to use community monitors (CMs). The CMs are residents, and all residents live in governmental structures of the communities called balozi, or clusters of 10 to 20 households. There are, on average, 30 balozi per community. When we collect census data, the households are grouped at this balozi level. We will hold community meetings at each community, and work with the community leaders to identify a CM from each balozi. Each CM must have the following characteristics:

- 1. Has access to a cell phone
  2. Is willing to visit each set of households in his/her balozi weekly
  3. Is well regarded within his/her balozi

The CM will visit each household in his/her balozi weekly. Once a family is identified as a newcomer or traveler, the CM notifies the community health worker (CHW); there are typically 2-3 CHWs per community. The CHW will be provided with a small store of azithromycin doses sufficient to treat the estimated 5% of new families that will arrive over the course of the year, and the 5%-10% of families that travel. If the family is classified as newcomers, then the CHW registers the family on the drug treatment list and verifies that they did not receive azithromycin during the community MDA. If the family is classified as travelers and absent from the community for more than 2 consecutive months and visited village(s) outside of Kongwa, then the CHW will administer treatment even if the family was part of the community MDA. The CHW observes treatment provided, just as in the protocol for mass drug administration. These data are reviewed monthly by a KTP supervisor.

The CM will be provided with a list of all families in his/her balozi with a check box for each week of the year, and a Newcomer form to add households to the weekly visit list(see Chapter 11: Study Instruments). After the first round of MDA for this trial (or in communities that have stopped MDA, after the baseline survey), the CM will visit each house in his/her balozi each week. If the household is present, the form will be ticked as “present.” If the household is not home, and the door is padlocked, the CM will confirm with neighbors that the family has been gone for at least a day and tick the household as “absent” (an unpadlocked door or one with children around indicates the mother is just temporarily out). They will also visit any newcomers in their area, inquire at each house on the list if a new family has joined the household intending to stay for at least a month, and while walking around will note signs of a new household (mud brick structure being erected). If there is a newcomer, then the protocol for newcomers will be followed, as described below. This surveillance will go on weekly.

If a newcomer family is found, the following steps are taken:

- The CM verifies that the newcomers are indeed new to the community, and have not moved from within the community (i.e., from another balozi).
- The CM will call KTP and indicate that a newcomer family has been identified. If the newcomer family has a new house, the CM will tell KTP that a new household number is needed. The KTP administrator will provide a household ID number, confirm that the household has come from outside of Kongwa district and record the call on a call log for the KTP supervisor assigned to that CM.
- In some instances a complete new family comes to the community to live with relatives. In this case, the family is added to the existent household, and the KTP administrator would provide the IDs available within the already existing household.
- The CM will record the assigned House ID on the door in chalk and if necessary arrange treatment for all members of the household with the CHW. The supervisor must verify that the household is indeed new and the supervisor will complete the newcomer census form
- The CM will determine if the family is eligible for azithromycin treatment, specifically if they:
  - - have children under ten
    - have moved from a community that has not had an MDA in the last year
    - plan to reside for at least one month in the community
- If the family is eligible, then the CM will explain that this community is part of a campaign to eliminate trachoma, and having arrived after MDA (or into a community that has been declared free of active trachoma), the community is offering the opportunity to the family to receive treatment for trachoma at this time. If the family is not eligible for treatment, the CM will explain that as a community participating in a research project, all families participate in a yearly census and updates of this census will be maintained for the research project.
- For families eligible for treatment, the CM will alert the CHW that treatment is needed. The CHW is responsible for seeking consent for treatment.
- The CHW would verify that the family is not on our treatment lists as having received drug during the MDA, and not on the census list developed prior to the MDA. Once this is verified, the CHW will seek consent for treatment and dispense the azithromycin to the entire family and record the fact that treatment was observed on the interim treatment form.

If a family has been traveling for 8 consecutive weeks and then at least one child returns after 8 weeks, the family is eligible to be considered a traveler, and the following will take place:

- The CM will determine if the family is eligible for azithromycin treatment, specifically if they:
  - - have children under ten
    - have returned from traveling outside the community for at least 8 weeks
    - traveled at least part of the time (>1 week) outside Kongwa district
- If the family is eligible, then the CM will explain that this community is part of a campaign to eliminate trachoma, and having traveled for at least two months outside the community, the community is offering the opportunity to the family to receive treatment for trachoma at this time. If the family is not eligible for treatment, the CM will explain that travelers with children or travelers who travel outside Kongwa are eligible for azithromycin but in this case, the family would not have treatment.
- For families eligible for treatment, the CM will alert the CHW that treatment is needed.
- The CHW will check if the family is on our treatment lists and provide treatment regardless if they had been treated in the past. The CHW will seek consent for treatment and dispense the azithromycin to the entire family and record the fact that treatment was observed on the treatment form.
- If the family members return at different times, it is the responsibility of the CM and CHW to make sure that treatment is carried out whenever each family member returns, until all children under age 10 years return, or the family indicates the children are not ever returning.

In all instances, if the newcomer or traveler family has arrived within three weeks of a scheduled MDA, they will be treated and a note provided to them that states they have had azithromycin treatment and it is not needed at the subsequent MDA. The only exception will be if travelers resume travel and are absent from the community for at least 2 months. The CHW will note on the treatment record the date that treatment has been provided, and when we enter the form, it will generate a note in the MDA treatment log for that family that treatment is not needed and was provided as part of surveillance.

We will provide prepaid cards to the CMs for phone calls to KTP. At the end of the month, the KTP supervisor will visit the CM and CHW, review the forms and ascertain the accuracy of the newcomer/traveler status, fill in the census forms for new households, and fill in all supervisory forms on their tablets, determine if treatment was indicated and provided and provide feedback to both the CM and the CHW. At that time, a small payment for time spent if there were no treatments administered, and a “finder’s fee” for the extra work with newcomers or travelers will be provided. In addition, this is the opportunity to identify any changes in the CM and provide training and supervision to new monitors if need be. The surveillance and treatment program will start after the baseline survey/MDA, and continue for two years.

## 4.3 Monitoring Treatment Coverage

### 4.3.1 Monitoring Mass Treatment Coverage

The census data that are obtained and updated yearly will be the primary tool used for monitoring mass treatment coverage. The programs that are built for data entry of the census data will generate a Mass Treatment list for each community. These master lists are used to record observed treatment for each resident of the community. The data entry of these lists is essential to monitoring coverage as the treatment is underway.

As data are entered, a report can be generated calculating the coverage of children ages 1 to 9.5 years. If coverage has not hit the target, a further report can be generated listing, in random order, all households with at least one resident who has not been treated, as well as the households missing treatment. These lists can be used to further guide the mass treatment steps described above, and the data from these reports are used to update coverage. At the end of mass treatment, these lists should categorize each community resident as: (1) treated or (2) not treated and the reason why.

After each community has completed its mass treatment, the full data set on treatment is sent to the principal investigator within a week in order to monitor the success of the treatment team in reaching coverage target. S/he will review the percent coverage of the community. If a community is over or under the target of 80% of children treated, the reasons why will be reviewed. This review will occur within three weeks of the completion of mass treatment so that if necessary the treatment team can be re-deployed into the community to improve coverage. If all residents are accounted for, and the community did not meet treatment coverage targets, the principal investigator will discuss ways to improve coverage for the second or third rounds with the treatment team supervisor (perhaps different timing or working with other community health team members, for example).

### 4.3.2 Monitoring Newcomer and Traveler Treatment Coverage

We will monitor the intervention in three ways. First, a supervisor from KTP will meet with their assigned CM and CHWs and review the census updates and treatment logs. Each CM leader and CHW must complete training to be certified for the trial (Section 12.3.6 Intervention and Supervision of Intervention). Each village team will start using their training immediately, even before the community has their baseline survey and MDA (if indicated), as practices sessions. Ms. Lee and Mr. Mkocha will supervise this practice activity after the training, together with the KTP staff supervisor, by making weekly visits to all CMs to observe house visits, and can institute corrective action at that point. After this initial training period is over, the KTP staff supervisor will visit intervention communities once per month. Identification of newcomers in the intervention communities is to be carried out within two weeks of arrival. At each monthly visit, if there are undetected new houses or travelers that have returned and are untreated, as identified from the spot check of five houses in the balozis, the KTP supervisor will do the update and make sure they are treated. A note will be made in the file for these households if they fell outside the two-week window. If one or more of these occurs in a month, the CM will undergo retraining and be put on probation for the next month pending a return to good performance. If the CM fails the next month to identify new households or track travelers, then they will be replaced by another community member who will be recruited and trained to carry out the activity. Second, a spot check of five of all the houses in one of the balozi for that CM will be undertaken every month to determine if a new household has entered the community and not been updated in our census files. This is easy to determine as all households have census numbers painted on the doors by our census team, so any new houses would have to have new numbers assigned by KTP staff (Section 7.2 Baseline Census) or chalk numbers on the doors awaiting supervisor finalization.

The CMs that indicate a traveler family has returned will have the file reviewed on when a family left and returned, or if they are still gone, and a spot check made of the household in the file. Third, when the census team conducts its yearly update, there should be no new homes in the intervention communities that are unknown to the CM and supervisor (unless the new family arrived within the previous week), and no new families added to households (unless they arrived within the previous week). If so, the data system will flag the household as previously unreported and the percentage unreported will be known.

Finally, the data manager will be reconciling the data generated from the KTP call logs with the supervisor forms received to ensure that all newcomer and traveler events are accounted for. The data management team also checks that all newcomer and traveler events that should be treated have been treated, and monitors the census update data at 12 and 24 months to be certain no new households are missed. Mr. Mkocha will take over supervisory activities if he receives from the Data Management team an indication that for any community on any one supervisory visit, more than 3 newcomer or traveler families that needed treatment were left untreated. We recognize that we are not capturing all the in-migration that may be potential sources of infection, for example, a child from outside who has come to live with a grandmother in the community. However, in-migrations within families, or short-term visits, are not feasible to monitor and our preliminary data suggest they are only responsible for <5% of infections one year after mass drug administration. To the extent these are also sources of infection that cause re-emergence, they should affect, but only modestly, the intervention and control communities equally. We can identify them as they will be represented in the random sample of children taken for the survey after the census update, and we can model them as a risk factor for infection after mass drug administration.

## 4.4 Masking Intervention

The trial is single masked, as it is not possible to mask the communities to the assignments they are to receive. The primary outcome is infection, and the laboratory is masked to the community assignment. The secondary outcome is trachoma, which is assessed in the field. The teams that supervise the implementation of the newcomer/traveler treatment program are different than the teams that will be undertaking the survey. The survey team will be unaware of the assignment to intervention. The survey teams do work in the community and may be told about the treatment program by community leadership, but since the primary outcome is infection, which is assessed by the laboratory, this should not be a source of bias.

The trial has several mechanisms to avoid bias in trachoma outcome assessment due to unmasking of assignment. First, trachoma assessment will be checked for consistency of each grader by reference to Dr. West, who will grade the images taken at each round. Dr. West will be masked to the treatment assignment as she grades the images, which are identified by ID number only. If the grades are below kappa=0.6 or show drift over time to lower grades, the grader will be suspended from surveys until he can be re-standardized. Finally, in the analysis phase of the study, we can evaluate the consistency of the relationship between clinical trachoma and infection across communities in the same arm; if the relationship differs markedly, especially in the direction that favors drastic reduction in trachoma unsupported by the infection data, we will report that bias due to unmasking in grading of trachoma may have been a factor. To date, these mechanisms have worked in the previous trial, and there was no evidence of drift over time or changes by treatment assignment in the assessment of trachoma.

Chapter

5

# **Chapter 5: Eligibility and Exclusion Criteria**

## 5.1 Eligibility Criteria: Communities

The communities are either one of the 48 communities that were part of the previous trial, or the four that were added by the DSMC request in December 2012. The criteria are described below:

1. The community leadership gives consent to participate in the trial (this does not obviate the need for individual consent, but without overall leadership consent, the community as a whole cannot be part of the trial).

## 5.2 Eligibility Criteria: Children

In order to be eligible to be a sentinel child, a child must meet the following criteria:

1. The child is between 1 and 9 years of age.
2. The child must be a resident in an eligible, sample community (defined as either living in the community since birth, or moved in with parents or guardians as a newcomer).
3. The child must not have an ocular condition that would preclude grading trachoma or taking an ocular specimen.
4. The child must be willing to have a swab taken as part of being a sentinel child.
5. The child must have an identifiable guardian capable of providing consent to participate.

## 5.3 Eligibility Criteria: Women

The census will also be used to randomly select 100 women from each community for the household air pollution component of the study. In order to be eligible, a woman must meet the following criteria:

1. The woman is at least 15 years of age.
2. The woman must be a resident in an eligible, sample community and have lived there for at least 2 months.
3. The woman must not have an ocular condition that would preclude grading trachomatous scarring.

## 5.4 Exclusion Criteria

Communities that do not meet the eligibility criteria will be excluded. In addition, if a political situation develops that makes it unsafe for our surveys to take place, that community will be excluded. In 25 years this has never been a reason for exclusion of a community.

A child will be excluded from the surveys if any of the following occurs:

1. The child is not in the village during the two days of the survey (in this case a randomly selected replacement child would be selected).
2. The child is too ill or has an ocular condition that precludes assessment of trachoma.
3. The child and/or guardian refuse to have an ocular swab taken at the time of the surveys.

A woman will be excluded from the surveys if any of the following occurs:

1. The woman is too ill or has an ocular condition that precludes assessment of scarring.
2. The woman refuses to be examined for scarring.

Chapter

6

# **Chapter 6: Consent Procedures**

## 6.1 Background

All procedures, protocols, and consent forms must be approved by the Johns Hopkins University Institutional Review Board (JHU-IRB) and the Tanzania National Institute for Medical Research (NIMR). Mr. Mkocha will have the responsibility of submitting the final MOP and application to NIMR in Tanzania within two weeks of finalization. His team will also be responsible for translation of all consent forms. Dr. West will have overall responsibility submission of the final MOP, consent forms and application to JHU-IRB within two weeks of finalization. All approvals must be obtained before the trial can commence.

All KTP study personnel who will be recruiting individual participants for the trial must receive training and pass certification in patient oriented research that includes emphasis on maintaining confidentiality of information. We have a course, adapted from the Patient Oriented Research Course used at JHU that has been approved in the past for this purpose, and this will be used again.

Persons in Kongwa who must pass this course are the intake clerks for the survey and the community health workers (CHWs) who will be dispensing azithromycin to the newcomer families and traveling families outside of regular MDA in the communities. The Community Treatment Assistants who provide MDA as a matter of the National Trachoma Program in Tanzania in the communities are not required to obtain research consent for their service.

Certificates of passing the course must be kept on file at KTP and available for review by the principal investigator and any other official from NIMR or JHU-IRB as required.

## 6.2 Consent Procedures

There are two levels of recruitment when working in Tanzania. The first is the level of the community, where community leadership decides if the project is one for which they wish to explain and build community support. Mr. Mkocha and Mr. Mchiwe will hold government leadership meetings in the communities to explain the new trial to the leadership during the interim surveys in 2012. KTP leadership will explain the following in each meeting:

1. The overall concept of “graduating” from MDA: if the data show the community level of infection is down to 1% or less, then the community residents no longer need mass drug administration and further exposure to antibiotics because there is likely only residual disease in the absence of infection.
2. If infection is not yet down to 1% we would plan to continue MDA in their community, but monitor the effects to see when the community can be “graduated” from MDA.
3. If over the course of the study, infection re-emerges to the level of 6%, that MDA will be re-instituted.
4. Updating the census is essential to the work, and can be used for Treatment logs for the community.
5. The need for further surveys of sentinel children in the community to determine level of infection, and the difference between infection and signs of trachoma. If MDA has been stopped in these communities, and the surveys find children with clinical trachoma, it is unlikely that infection is present. If families wish, they can seek topical tetracycline from the CHW for treatment.

In the intervention communities, further explanations will include the following:

1. The nature of the surveillance and treatment program for newcomer and traveler families and that we wish to evaluate if such a program helps keep infection at a low level.
2. Balozi residents will be chosen through community meetings to be CMs in their neighborhoods, and the Community Health Worker (CHW) will have a store of azithromycin if azithromycin treatment is needed for these families. All training for these positions will be held at KTP.
3. In communities where MDA is stopped, a store of azithromycin will be kept by the CHWs for the purpose of treating these families only. Once MDA is stopped it is unlikely that any one else will have infection with *C. trachomatis* needing azithromycin, but if during the course of the survey we see clinical trachoma, we will inform the families of the sentinel children they can seek topical tetracycline in local shops or from the CHWs.

The KTP team elicits verbal agreement to be part of the census. Since the census provides the data base for MDA, the community leadership needs the census information to monitor treatment coverage which they report for the Tanzania National NTD Program. At the point of census we explain the following:

1. The children and women in the family may be eligible to be in the survey that will be done in a few days.
2. Participation of the child in the survey entails trachoma assessment, and a swab taken of the upper eye lid to detect *Chlamydia*, the bug that causes trachoma.
3. Participation of the women in the survey entails trachomatous scarring assessment, and swabs taken of the upper eye lid to detect *Chlamydia* and other bacteria that cause eye irritation.
4. Participation is voluntary, and will not affect access to any services.

Once the random sample is selected, invitations to the survey for the sentinel children and women are printed and delivered to the community the day before the date of the survey. When the selected participants come for the survey, the intake clerk seeks individual guardian-level consent for the children, assent from the children aged 7 years and older, and individual adult consent from the women. The family will have had time to think through participation, since the survey takes place at least three days after the census.

The draft consent forms are shown in MOP Appendix 2 in English, and may be modified by JHU-IRB and/or NIMR. They have also been translated to Swahili, and if necessary, the intake clerk will also translate into Kigogo or Kikaguru.

The intake clerk will demonstrate the use of the swab that is used in the examination, and explain that participation is completely voluntary and will not affect access to health care. For the sentinel children, the explanation of the project to the parents is in the presence of the child, and they have an explanation of what the examination entails. The intake clerk will make every attempt to gain assent of the children ages 7 years and older, using a simple explanation. He will obtain verbal assent from the children ages 7 and older (school age), with the guardian and intake clerk as witness. For the sentinel women, the intake clerk will explain the project to and obtain consent from each woman directly. Understanding of the exam will be assessed using the following questions:

1. Do you know what this survey will be looking for? (Trachoma)
2. Do you know what the exam will involve? (flipping eyelids to look for trachoma)
3. Can you refuse to participate? (yes)

The intake clerk will explain to the family in the communities that have had MDA stopped that infection is very low in this community. Therefore, MDA with azithromycin has been stopped. If the examiner finds clinical signs of trachoma, it is unlikely that there is still infection, so no treatment will be offered at this time. The family can seek treatment from the CHW if they wish. Finally, all children who have tested positive for *C. trachomatis* and are in the “graduated villages” will receive azithromycin at the end of the study to be certain treatment was provided. Further MDAs will be at the discretion of the National Trachoma Program.

In the intervention communities, the CHW will be seeking consent from newcomer families for census and family based treatment and from traveling families for family-based treatment. When the CM identifies the family, s/he will seek verbal consent from the newcomer family to participate in the census, as that is the only way to determine eligibility as newcomer. S/he will explain the following:

1. This community is participating in a community effort at trachoma control, and that the community is undertaking a newcomer and traveler treatment program to see if that helps with control.
2. Participation in the census is for the purposes of research, and if the family is found eligible for azithromycin, will be offered azithromycin at this time by the CHW.
3. Participation is voluntary, and the family can participate or not in the census, and participate or not in taking the drug if the family is eligible.
4. If the family is eligible and decides to take treatment, the CHW will obtain written consent for family-based treatment.

In the intervention communities, the CHW will be seeking consent from the families that have traveled more than 8 weeks for family-based treatment. The community leadership will already have explained the program to residents, so the family will have heard of it, but the CM will explain that the program to treat families who travel for long periods of time is to evaluate if such a program helps with trachoma control. If the family is found eligible for azithromycin, they will be offered azithromycin at this time by the CHW. Participation is voluntary, and the family can participate or not in taking the drug if the family is eligible. If the family is eligible and decides to take treatment, the CHW will obtain written consent for family-based treatment.

## 6.3 Risks and Efforts to Minimize Risk

Flipping the eyelid is only slightly uncomfortable and most individuals are not bothered by the procedure if properly explained. The swab is uncomfortable, and can be a painful procedure if the eye is very inflamed. However, the procedure does not harm the child, other than upset them for a few moments if it is uncomfortable. A single individual flips the lid while the senior trachoma grader carries out the examination, takes the swab, and the photograph of the upper eye lid. This method minimizes the risk of any psychological trauma to the child, or discomfort from having the eyelid flipped, and allows the grader to work quickly. In addition, by holding the eyelid, the swab procedure can be done safely without worry of accidental corneal scratch. In the unlikely event of a corneal scratch, topical tetracycline will be applied and a patch applied to treat the scratch immediately. We have had no corneal scratches in the 10+ years of carrying out trachoma research, with over 240,000 lids flipped.

There is the theoretical risk of transmitting trachoma from one individual to another during the examination, which is minimized by strict adherence to sterile technique and the changing of gloves between each individual by all who contact the individual. These procedures have shown no evidence of contamination and should reduce that to virtually zero. All portions of the swab handle, gloves, and any tissues used are discarded in a biohazard bag in the field and taken to Kongwa District Hospital for disposal.

To protect confidentiality, all study forms are stored in a folder that only KTP staff has access to when in the field, and after data entry, all forms are kept in a locked file room.

For communities that have infection at 1% or less, subsequent MDAs are stopped. The Data Coordinating Center will be monitoring these communities for re-emergence of infection. Ms. Munoz will alert Mr. Mkocha using a notification form if a community has shown re-emergence of infection to 6%, and MDA will be reinstituted after the subsequent survey. For children who tested positive for infection at any survey in the “graduated” communities, the Data Coordinating Center will release a list of their study ID numbers at the end of the trial to Mr. Mkocha at KTP, who will ensure that these children receive the opportunity to take azithromycin.

Chapter

7

# **Chapter 7: Census Procedures**

## 7.1 Overview

The census is a critical piece of data collection, as it is the document by which we measure coverage of mass treatment in the community, and it is the sampling frame for drawing a random sample of 100 children in each community at each survey, and the 100 women in each community at baseline. The census is updated at each year. Within the intervention communities, new households that come into the community are detected by CMs at the time of arrival into the community. Census data is collected at that time. For newcomers to the usual practice communities, newcomers are detected at the time of the regular census update each year.

For new additions within households (due to birth, for example), or loss of persons in a household (due to death, for example), addendums to the census list will be made indicating the age, gender, and relationship to head of household for the additions, or reasons for deletion of a person (death or permanently moved to another community). If the person has simply moved within the community, they will be deleted from their previous household and added to the new household using the Census Update Book Form (see Section 11.2.2 Census Update Book (Form 2)).

These updated census lists will be used to draw a new random sample of 100 children at each yearly survey, provide the denominator for the mass treatment interventions, track new families coming into the community, and measure the success of the community team in catching all new residents. The Census form is shown in the attached MOP Appendix 1: General Study Forms.

## 7.2 Baseline Census

Once the communities are randomized, a census is carried out prior to the survey and MDA, if indicated. This baseline census data is used to collect information on each household and create a central document that provides information for the selection of households with eligible children and women in the community. Additionally, the census is the basis for the creation of the master list for surveys and the database for mass treatment. Together these are used to track who has received treatment within a community. At the time of the census, a questionnaire will be used to also collect data regarding environmental factors at the household level that may be important for trachoma.

A household is defined as a unique doorway belonging to a family. The definition of resident in the household means a person who has slept in this household for at least three months in the past (or if age less than 3 months, was born in the household) or who intends to reside with the family for the next six months. The census team will meet with the community leaders, and collect a list of the head of household names. Each of the community leaders will assign a knowledgeable community resident to escort the census team member to the households under his/her jurisdiction. The household will be assigned a unique identification number by KTP, which will be affixed to the door. These numbers will correspond to a community number followed by a unique three digit ID for that household (See Census Form, in MOP Appendix 1: General Study Forms). At the end of the census, the census team leader will go through the community to be sure that there is no door that is missing a number. If a household prefers not to have a permanent number affixed to the door, they will be asked to at least have a plastic sheet with the number taped to the door for the duration of the census so that they are not visited and enumerated more than once.

For each household, the census team member will ask to speak with either the male or female head of household to obtain a list of the names, ages, and gender of all persons resident in the household. The definition of resident in the household means a person who has slept in this household for at least three months in the past or who intends to reside with the family for the next six months. The ages of the children need to be as precise as possible, as they comprise our sample. Vaccination or Maternal and Child health clinic cards will be used to obtain age, and we have them on over 80% of children. Otherwise, event calendars specific to each location are used to tie ages to such events as elections, weather events, significant deaths, etc.

Other demographic information collected includes education completed by the head of the household, distance to the closest source of water, observations on clean face status of children aged 5 and under, presence of latrines, cleanliness of the area around the doorway of the household, and location and use of the household’s cooking fire. These measures enable us to further characterize households in communities where sustained reduction in disease and infection has occurred. The census data are entered into the database using customized data entry forms in Access that are standard across all clinical sites. This component is described in Chapter 14: Data Management and Routine Reports.

At census updates, the same census information will be collected on the household and each person in the household as was collected at the baseline census, except for the data on cooking fire use and location, as this special survey will be done only at the baseline census. New data to be added at updates include cell phone numbers, and an indication, if they are new to the community since the last census, how long they have lived in their present location. Addenda to the census list will be made indicating the age, gender, and relationship to head of household for the additions, or reasons for deletion of a person (death or permanently moved to another community). If the person has simply moved within the village, they will be deleted from their previous household and added to the new household using the Census Update Book Form (see Section 11.2.2 Census Update Book (Form 2)).

## 7.3 Newcomer and Traveler Census Procedures

In the 26 villages randomized to treatment of newcomers and travelers, the CMs are the primary point of contact for updating the census at the time of arrival. The CMs will use the Community Monitor Household Checklist (see Section 11.5.3 Community Monitor Household Checklist (Form 8)) to verify that each household within their community was present at the last verification during the previous week. If no one within a household is present at the time of the verification, then the CM marks the household as ‘absent.’ If a household is absent for eight weeks or more in a row, and then subsequently returns, they are classified as travelers. If a new household appears, then the CM will update the census with the newcomer information, and determine if the new household is a newcomer to the community according to our definition (this definition defines who will be treated, but all newcomer families regardless of needing treatment will be added to the census).

If newcomers appear during the CM’s check, the CM will explain to them that the community is part of a campaign to eliminate trachoma, and having arrived after MDA (or into a community that has been declared free of active trachoma), the community is offering the opportunity to the family to receive treatment for trachoma at this time, as well as be a part of the community census. Then, the CM contacts the KTP headquarters via cellular telephone to obtain a household ID, and to verify if azithromycin treatment is required (see Section 4.2 Newcomer and Traveler Treatment Intervention for further details on treatment of newcomers and travelers). The Supervisor at KTP completes a KTP Supervisor Form (see Section 11.5.5 KTP Supervisor Form (Form 10)) to record the presence of a newcomer or traveler and verify that treatment was needed and provided.

The CM leader next notifies the CHW for his balozi; there are typically 2-3 community health workers per community. The CHW visits the newcomers/travelers and ascertains whether azithromycin treatment is required (see Section 4.2 Newcomer and Traveler Treatment Intervention for details), and completes a Newcomer/Traveler Treatment Form (see Section 11.5.4 Newcomers & Travelers Treatment Form (Form 9)).

### 7.3.1 Newcomers Census

Once a month, KTP intervention supervisory team staff members will visit each of the 26 communities randomized to the intervention. During this visit, they will record census data for all newcomers using the Newcomer Census Form (described in Section 11.2.1 Add New Household to Census Form (Form 1)). This form ascertains similar census information as the baseline census, but also collects information about the presence of children under 10 years of age and the location of previous residency. If the family has no children under age 10 years, or the newcomers came from a community within Kongwa, they will be recorded in the census update but not treated. As described in Section 7.2 Baseline Census above, newcomer households will be assigned a unique identification number which will be affixed to the door.

Newcomer Census Forms will be entered into the computer each month by the data entry team as verified by the KTP supervisor for that CM, but not merged with the primary census files for that community until the end of the year when the full community census updates are carried out. The reason for this is that the interim surveys (those at 6 months post full census update) use the census data from the previous updates in communities in both arms of the trial to draw the sample of children. If we included newcomers and travelers as eligible for the interim surveys only in the intervention communities, then there would be a bias in the estimate of infection as these newcomers are preferentially treated. However, all newcomers in both intervention and control arms would be eligible for the 12-month surveys, so at that point those files can be merged.

### 7.3.2 Traveler Census

For this trial, a traveler is defined as a community resident who leaves for eight or more weeks and then returns to their community. If a CM identifies travelers during their weekly verification process, then no census update is required, as these individuals are considered community members and have already been included in the previous census update.

## 7.4 Census Update Procedure

### 7.4.1 Surveys and Mass Treatment Updates

After the baseline census, surveys will be undertaken and mass treatment provided. Each of these contacts provides an opportunity to update the census information for the village. During the ocular exam survey visit, any child that is marked as ‘deceased’ has his/her master census information updated upon entry of the Ocular Exam Form (see Section 11.4.2 Ocular Exam Form (Form 3)) into the database. During the mass treatment visits, the status (absent/present) of each resident is also updated. Also, new residents can be added to the master census list using the Mass Treatment Book (see Section 11.5.1 Mass Treatment Book (Form 6)). In the intervention communities, the newcomer surveillance does not start until after the MDA, as by definition the newcomers missed the MDA in the community.

### 7.4.2 Yearly Census Updates

The census will be updated every year in preparation for drawing a new sample and undertaking mass treatment. A master census list of each house will be printed, using the most recent census update data (see Section 11.2.2 Census Update Book (Form 2)). In communities randomized to MDA plus treatment of newcomers, this list must be updated using the most recent survey and ‘newcomer’ surveys. For the communities randomized to MDA alone, this list will be obtained from the previous census. The census takers will use this book to return to each house and update the census.

Chapter

8

# **Chapter 8: Study Visits**

## 8.1 Overview

The purpose of the study visits is to collect the basic data on the sentinel children who will represent the community burden of trachoma and ocular *C. trachomatis* infection over time, and the women who will represent the community burden of trachoma scarring at baseline only. Once the 100 sentinel children are selected in each community, the study team will examine them for trachoma and take swabs for determination of laboratory evidence of infection. Swabs are sent to the JHU laboratory for processing. New, cross-sectional random samples of children will be drawn at each study visit, using the latest census survey as the sampling frame.

For logistical reasons, the teams cannot make more than two visits to a single community, so persons who are not available for the examination within two days will be replaced from the alternate list as described in Section 8.4 Follow-up of Children. The following describes our procedures.

The study team for the exam visits consists, at a minimum, of an intake clerk, a trachoma grader/photographer, lab technician, lid flipper, and a driver.

The first mass treatment is carried out immediately after the baseline survey. Subsequent follow-up surveys are at 6, 12, 18, and 24 months from the first mass treatment and in communities at risk of trachoma re-emergence at 18 months, an additional follow-up survey will be conducted at 30 months. The census lists are updated formally every year prior to the 12- or 24-month survey. The 6- and 18-month surveys will use the information from the last updated version of the census for the random selection of the sentinel children. The window for each survey is +/- two months. The exact same procedures as stated for the baseline examination visit are to be followed for all subsequent examination visits, except that women age 15 and older are only included in the baseline survey.

## 8.2 Community Level Visits

The director of the KTP and his census team meet with the community leaders to explain the project, and meet again with them at annual intervals to report progress, problems, and get feedback. All communities have been approached about this new trial, and the leadership has agreed to have the communities participate. The KTP and Johns Hopkins University have worked in these communities since 1986, and are trusted partners in the leadership quest to improve the lives of their residents. The leaders are given feedback on the results of the mass distribution, the current status of trachoma in their communities, and other metrics on clean faces and latrines, which are important for community leadership awareness. They in turn use their networks to explain the project to residents, answer questions and raise awareness of project goals and needs.

## 8.3 Survey Visits

Once the census data is entered and the random samples of children and women have been selected, “invitations” (chetis) are issued and delivered the day before to each family inviting the children (with guardians) and women (baseline survey only) to the survey, located in the neighborhood. See Figure 8.1 for example invitations.

**Figure 8.1: Invitation Cards**

The day of the survey, the invited individual is checked in by the intake clerk. The intake clerk has a master list of selected sentinel children and women for the community. The intake clerk checks off the individual as having arrived on the master list. The intake clerk completes the informed consent procedures, described in detail in Section 6.2 Consent Procedures. After obtaining consent (and assent if the child is school-age), the intake clerk signs the form(s) as the witness. The consent/assent forms are stored at Kongwa Trachoma Project offices. The intake clerk makes a note for the trachoma grader if the child has been selected for an ocular photograph, used as quality control procedures for the trial. The notation and all the bar code labels are then sent with the child (with guardian)/woman to the survey station. The women will also be asked a few questions to estimate their lifetime exposure to cooking fire.

The intake clerk keeps a running count of the children and women who are examined and notes those who have failed to show up for an appointment. He assigns the CHW to go to the households of children and women who have not appeared and determine the reason. We will make every effort to include such children and women, including offering transportation to and from the exam site, or a home visit if necessary. If the child/woman has moved permanently or has died, then the intake clerk will select a child/woman from the alternate list and complete a census update form (see MOP Appendix 1: General Study Forms) for the child/woman whose status has changed. If the child is not available during the examination times in the community due to refusal, travel, or illness, the reason is noted on the Master list, and an alternate child/woman is selected.

At the end of the day, the intake clerk must account for all children and women on the master list of selected sentinel children and women. Either they have presented for examination, or a reason is provided for nonattendance, and an alternative appointment scheduled or an alternate selected. If the list is incomplete, the survey team must wait until households are visited and the child/woman is examined, another appointment is made, or an alternate child/woman is examined.

The following describes the details of the procedures for the examination. All ocular data is collected on a Samsung tablet in real time, which includes an ocular form and a bar code scanner for the swab vial. Details on operation of the tablet are provided in Appendix 3: Use of Tablets for Data Capture. The ocular form component is described in Section 11.4.2 Ocular Exam Form (Form 3).

### 8.3.1 Trachoma Assessment

Before the examination, the grader should examine the ocular form, to be certain it contains labels for the swab and check if a photograph is necessary. The examiner should call out the name and ascertain that the child/woman (by name age and gender) is correct. If incorrect, the child (with guardian)/woman should be sent back to the intake clerk for resolution-either the form sent in was incorrect, or the wrong child/woman was brought in for the examination.

For the examination, the upper eye lids of the children and women will be flipped. To examine the children for trachoma, it may be necessary to have the guardian hold the child securely in their lap, with the child’s legs around the guardian’s waist and the guardian holding the child’s hands. The child’s head is then in the lap of the examiner/lid flipper. The child is told to look “down” at the guardian, as it is easier to flip lids. Older children and women are told to look down at the ground. Once the person who flips the lids has on a new pair of gloves, s/he should NOT touch the participant anywhere else but on the eyelid to prevent contamination. Trachoma is assessed by flipping the upper eye lid and examining the tarsal plate for evidence of trachoma. Lid flippers are instructed to ensure that pressure on the everted upper eye lid is not too strong or it will blanch and make grading very difficult. The lid flipper may need to un-flip the lid between grading and photography, if photography is required. Photography will be taken on a random sample of children and all children with trachoma and on the right eye only. Photography of the everted lid will be taken on both eyes of the women, as scarring as graded on photographs will be the outcome for the risk factor survey. The left eye is flipped first, and graded (and photographed in women), followed by the right eye; the right eye is then photographed (if selected, and in women) and swabbed.

Trachoma is assessed using the WHO simplified grading scheme for trachoma, which assesses the presence or absence of follicular trachoma (TF), intense trachoma (TI), conjunctival scarring (TS), trichiasis (TT), and corneal opacity (CO).[1] For this study, the relevant signs are TF and TI, signs of active trachoma in children, and TS in women. See Figure 7.2 for an example of TF and TI. TF is defined as five or more follicles in the region of interest of size 0.5 mm. If there are fewer follicles and/or if they are of insufficient size, then TF is graded as not present. TI is defined as inflammation severe enough to obscure 50% of the deep tarsal vessels. TS is the presence of any visible scarring, and is graded on the basis of the photographs of the right and left eye. The senior trachoma grader uses a 2.5 loupe and a torch for grading, and the lid flipper and grader change gloves between each individual. All data are recorded on a Samsung tablet which contains the ocular form, which captures information about the grading, the taking of photographs, and the taking of ocular swabs (see MOP Appendix 1: General Study Forms). For details describing quality assurance methods see Section 13.7 Quality Assurance and Training for Field Trachoma Grading.

The gradings should be called out to the data recorder who will enter the grades into the electronic ocular data form for each eye. The grader should call out the left eye grades for all signs before everting the right eye. The recorder must be certain to enter in grades for each sign for both eyes.

**Figure 8.2: Trachoma Grading Card**

### 8.3.2 Ocular Photography

Overview: An ocular photograph will be taken of the right eye of every 5th child plus all children with trachoma sufficient to ensure at least 50 photographs per trachoma grader per survey round. This addition of images form all children with trachoma should provide a pool of photographs with adequate cases of TF/TI for standardization purposes but if there are insufficient TF/TI cases, the images will be supplemented with images from previous surveys. . Ocular photographs will also be taken of both eyes of all women surveyed.

Within the data file of every community, a record is kept of the random sample of children chosen for the survey, and within that, the sample of children chosen for photography. The intake clerk will indicate to the trachoma grader if the child has been randomly selected for an ocular photograph, and/or if a “blue air” control swab should be taken after the examination. The extra bar code labels are placed in the photo log book (see Section 11.4.3 Photo Log Book (Form 4) and in the swab log book for the blue air swab. The Grader will indicate if the child is selected for photography because the child has trachoma.

Clinical photography will be performed before conjunctival swabbing. A handheld Nikon D-series camera (D-40) with a 105mm f/2.8D AF Macro Nikkor Autofocus Lens (fully extended, in manual setting) will be used. The first photo is taken of the study ID. This is followed by at least two photos of the upper conjunctiva. Photos are immediately reviewed for gradeability and retaken if necessary. (Note that we have achieved >95% acceptable photos in previous studies.) Images are stored in digital file folders according to study ID number. They are burned onto a CD and sent to JHU, where they are graded for quality and then for trachoma, as described under quality assurance procedures. All photographs of eyes of the women at baseline will be burned onto a CD and sent to JHU for grading of TS by two trained trachoma graders, with adjudication by the master trachoma grader. Details on photography procedures are provided below.

### 8.3.2.1 CAMERA SETUP

Necessary equipment: camera, labels, photo log book, and CD burner

Miscellaneous equipment: extra battery, card reader, lens filter, extra media card

A handheld Nikon D-series camera and lens will be used for all photographs. If possible, use natural light rather than a flash, as the flash introduces artifacts into the image.

1. Model
   1. Nikon D-series camera: D-40, 50, 70, 80, 90, 100 or 200
   2. There are minor differences between the different models, mostly related to setup
2. Lens
   1. Nikon Macro Autofocus 105 mm f/2.8
   2. Manual setting- turn off autofocus
   3. Disengage “limit” engage “full”
   4. Extend lens to 1:1; note with older versions of this lens it is 1:1 at full extension; newer versions can go to slightly higher magnification than 1:1 so caution must be exerted
3. Image size/resolution
   1. JPG normal- set thru “setup” menu
   2. Large- set thru “setup” menu (“small and medium” are probably adequate)
4. White balance- automatic or flash; set thru “setup” menu
5. Shutter- Aperture Priority
   1. f57- set thru menu; note if lens is not extended to 1:1, f57 will not be allowed
6. ISO 400

### 8.3.2.2 PHOTOGRAPH PROCEDURE

1. Place patient into position that will allow maximum stability; standing, sitting or “head-clamp” position. Employing village volunteer to help is very useful.
2. Take photo of patient ID.
3. Image is brought into focus by changing the working distance, not by turning the lens. This is because the lens is fixed in its manual setting. The working distance is approximately 20 cm from the eye with our current settings.
4. Take minimum of 2 photos. If there is any doubt of the quality of the photo while the patient is in position it is better to continue to take more photographs before the patient is allowed to leave. It is easy to delete photos if they are not needed.
5. Check photos before allowing child/woman to leave. If they are not acceptable, repeat procedure. Only stop if the patient or guardian requests that we stop, or if it is deemed impossible, even with further attempts. Note we have obtained >95% acceptable photos in previous studies.
6. If the child/woman cannot be photographed for some reason, it does not affect the eligibility of the child/woman. A notation is made on the ocular form of why the photograph cannot be taken, but no replacement is sought. Again, this is expected to be a rare event.
7. The ocular form contained in the tablet is used to maintain a record of each photograph’s location (Section 11.4.2 Ocular Exam Form (Form 3)).

Images are stored in digital file folders according to study ID number. They are burned onto a CD every week and sent to JHU where they are graded for quality assurance purposes by Dr. Sheila West, who is the standard grader for the ASANTE study. This is discussed further in Section 13.7 Quality Assurance and Training for Field Trachoma Grading.

### 8.3.2.3 PHOTO TROUBLESHOOTING GUIDE

Note all camera settings are permanently embedded in every photo that is taken and can be viewed with the camera or with any standard commercial photo-viewing software (e.g., Adobe Photoshop; Photomechanic, Nikon View, Canon, etc.)

1. Photos too DARK, too LIGHT or OUT OF FOCUS
   1. Check camera settings: ISO 400, Aperture preferred, +/- on zero, Manual setting on lens, flash elevated; battery fully charged
   2. If all photos are affected in the same way than it is most likely the settings on the camera or lens
2. Change camera settings from default recommendations
   1. Decrease F stop to lighten, increase to darken
   2. Could be taken down to 32 or lower but depth of field will be lessened
   3. +/- can be increased or decreased to change exposure (+ to lighten; - to darken)
   4. Check that lens is on Manual NOT Autofocus
   5. ISO- increase to lighten photo, decrease to darken
   6. Very high ISO will produce graininess
   7. Meter- try “spot” instead of “pattern
3. Have another person observe photography in real time
   1. Make sure flash is not depressed by photographer or someone else while photo is taken. (This commonly happens if photographer has loupes). Is the lens well supported?
4. Make sure battery is fully charged
   1. If first photos in series are acceptable and then they gradually become less exposed (darker) it might be because the battery is gradually losing power during the session. Note that the flash reaction time increases as the battery power decreases.
5. Out of focus
   1. F stop may be too low; movement artifact; flash doesn’t work; lens is dirty
6. Image not centered
   1. Movement of person or camera (for children, hold head between knees of “helper”; stabilize camera with second hand)
7. Reflection artifact
   1. Move camera slightly between first and second photos to achieve different angle; gently dab conjunctiva with swab- must be done at periphery to avoid creating inflammation

### 8.3.3 Laboratory Specimen Collection

Overview: All sentinel children and women will have ocular swabs taken for determination of infection. The children will have one swab taken from the left eye, and the women will have two swabs taken, one from each eye. If a child refuses the swab, or a swab cannot be taken for another reason, then the child is ineligible to participate in the study. The intake clerk should be notified that the child is ineligible, a note made in ink on the ocular examination form and in the master list of selected sentinel children, and an alternate household and child selected as replacement.

Each child/woman has a set of printed bar code labels with the child’s/woman’s study identification number. One label is placed on the study vial after a swab is inserted, and one on the shipping log for the box in which the vial is inserted. The bar code is scanned by the tablet at the time of the examination, as a record the specimen was taken. In this way, each vial box has an accounting of specimens for subsequent shipping. There are extra labels as well, if one is lost or torn. We will be taking a field control swab for each laboratory technician sufficient to have at least a 5% sample per survey. These will be taken using the “blue air” swabs that will be pre-marked in each box of swabs.

### 8.3.3.1 PROTOCOL FOR SWABBING OF THE CONJUNCTIVA

We will be taking a field control swab for each grader sufficient for a 5% sample of control swabs per survey. These will be taken using the “blue air” swabs that will be pre-marked in each box of 100 swabs.

1. Upon taking a swab from the box, the lab technician should announce what type of swab it is:

- An unmarked “plain swab”, indicating no control collection necessary for the patient.
- A "blue air swab" (negative field control), indicating examiner must collect a second swab for the patient. Swab sachet will be marked with blue color.

1. The swab sachet should be opened in a sterile manner, revealing only the tip of the swab shaft, with the swab head itself remaining sterile deep within the sachet. The grader pulls out the swab with the gloved hand, held no closer than 1 inch from the swab tip during the entire swabbing procedure to avoid contamination
2. As the lid flipper holds the left eyelid in the everted position, the grader swabs the upper tarsal conjunctiva with a gloved hand, using a steady and firm swab. Ideally the swab should be placed flat, with its entire length parallel to the conjunctiva. The swab should be drawn firmly in one direction over the conjunctiva with enough pressure to cause blanching of the conjunctival vessels. The swab should then be rotated 120 degrees along its axis and the newly revealed fresh region of the swab tip should now be drawn firmly across the conjunctiva. The swab is rotated another 120 degrees along its axis and the conjunctiva swabbed for a third time. This will ensure sufficient collection of conjunctival epithelial specimen for PCR analysis in the lab.
3. The technician opens the capped lid of the swab tube. The swab is inserted by the grader (using the still-gloved hand that swabbed the participant’s conjunctiva) into the tube held by the technician. The swab shaft is inserted until the Dacron swab head is fully in the tube. The technician lowers the cap onto the swab shaft and the examiner quickly breaks the swab shaft. The cap is replaced tightly and placed in the sample collection box, located in the cooler bag filled with frozen ice packs.
4. Steps 3 and 4 are repeated for the swabbing of the right eyelid of each woman, except the swab from the right eye will be placed immediately into transport broth and kept cold in the field using ice packs. The second set of swabs collected from the women will be stored in a separate collection box.

**Note:** An aggressive twisting motion toward the cornea can push the conjunctiva upwards (towards eyebrow) which can lead to the swab falling onto cornea. Thus a pure lateral motion or slight twisting toward the eyebrow is preferable, in order to ensure safety of the cornea at all times. Quick movements should be avoided (less control, increased contamination, risk of touching the cornea). Instead, a slow, steady motion should be carried out. Care should be taken to avoid swabbing the eyelashes. Traction on the lower lid by the non-swabbing hand (or by an assistant) can keep the lower lid lashes from inadvertently touching the swab.

### 8.3.3.2 PCR CONTROL SWABS

A negative field control ("blue air swab") will be taken on a randomly chosen at least 100 children/women per lab technician/grader to assess the frequency of contamination. For each negative field control, the examiner will pass a sterile Dacron swab within 1 inch of the individual’s conjunctiva.

Control swabs are taken after the original swab but before changing gloves for the next patient. A label from the control set, made to look like a regular ID but indicating a control swab and generated from the data coordinating center, will be used. One label is affixed to the ocular form in the “control swab” area and one is affixed to the vial, and a third to the ocular specimen shipping list.

The swabs are processed by the laboratory masked to intervention or control village status, and masked to their status as “blue air” controls.

### 8.3.3.3 PROTOCOL FOR TUBING AND HANDLING OF SAMPLES

The tubing and handling protocol must be carefully followed in order to prevent contamination and ensure the safe transport of the samples back to the microbiology laboratory. The person in charge of labeling, tubing, arranging, and handling the samples needs to perform this task in the most orderly and attentive manner.

- 1. Both hands of the tuber should be gloved at all times. The tuber's gloves only need to be changed when any potential contamination of the gloves occurs. The tuber opens the capped, hinged lid of a microcentrifuge tube, which has been labeled with the participant’s random identification number. If it is a control or “blue air” swab, then the label comes from the control set of labels.
  2. The swab is inserted by the grader (using the still-gloved hand that swabbed the participant’s conjunctiva) into the microcentrifuge tube held by the tuber. The swab shaft should only be inserted until the Dacron swab head is fully in the tube. The tuber should lower the cap onto the swab shaft held by the examiner, and the examiner should quickly break the swab shaft using a swift downward snapping wrist movement.
  3. The tuber should screw the cap of the microcentrifuge tube tightly and place it in the sample collection box, located in the cooler bag filled with frozen ice packs. The flap of the cooler bag should be closed between each patient. The cooler bag should be in as cool a place as possible in the field, in a shaded area out of the sun.
  4. Upon returning from the field each day, the samples will be immediately taken to the field office and stored in a refrigerator, reserved solely for storage of specimens. All samples will be in sample boxes, numbered for easy future identification.

### 8.3.3.4 SHIPPING AND STORAGE OF SAMPLES TO JHU LABORATORY

Swab samples taken in the field will be transported on ice in a closed, insulated container, stored at KTP at 4°C, and then shipped within 30 days and up to a maximum of 40 days of collection to the laboratories in Baltimore where they will be stored at -80°C for later analysis. Samples will be imported in the USA as per the CDC Permit to Import/Transfer Etiological Agents or Vectors of Human Disease and Materials Transfer Agreement with Tanzania.

Materials needed for shipping to the US:

- Insulated shipper box (ThermoSafe VIP insulated shipper, 615DCS or 499 DCS)
- Gel Packs (ThermoSafe U-tek 24 oz. -10°F)
- Shipper label
- Consignee label
- Responsible person label
- UN3373 label
- Biological substance, Category B label
- Documentation to include in shipper box:
  - Pro-forma invoice
  - Letter from Shipper
  - CDC permit
  - Letter of approval from Ethics committee in country
  - Airway bill from DHL or other air transport service

For dry samples without media:

- Boxes can be wrapped with plastic wrap and clear plastic tape only. There is no need to use pressure-resistant bags.
- Boxes may also be wrapped with a cotton absorbent pad (chuck) for improved insulation.
- As soon as samples are prepared for shipping, they should be driven to Dar es Salaam for air transport to JHU
- Place all stickers on outside of the shipper box (see detailed shipper instructions).
- Leave at least one shipping box, preferably a partially-filled one, unpacked for inspection by the air shipper employee and or customs if need be

Shipping instructions include the following:

- For a small shipper box: Use 6 frozen gel packs- one below sample boxes, 4 on each side, and one on top of sample boxes
- For a large shipper box: Use 12 frozen gel packs- 6 gel packs flat at the bottom and 6 on the top
- Immediately after the shipment is accepted by the shipping company, the shipper box should be closed and taped shut using clear plastic tape. Tape should be placed around all corners of the outside box.

When ready to ship, the prepared sample boxes should be placed into the partially filled shipper box. Next, all frozen icepacks and samples should be placed in the shipper box. Packing is done the morning that the car is ready to go from Kongwa to Dar es Salaam. The driver proceeds straight to DHL offices in Dar es Salaam, and they ship with instructions to maintain cold to the laboratory in Baltimore, with two-day turn-around. Specimens should be tested within 40 days of collection, but we can freeze specimens at -20 to -70°C for up to 90 days before assaying. The specimens are processed according to strict protocol, outlined in the manufacturer’s kit directions, followed by the Johns Hopkins International Chlamydia Laboratory.

## 8.4 Follow-up of Children

All children on the master list of selected sentinel children for each community will be followed up at their household if they do not present for examination. Families will be offered either free transportation to the examination site and back or a home visit, in order to assist in overcoming barriers to the examination. If the child/household is temporarily away, another appointment will be set up for a second day. If the child is too ill to be examined, refuses, or has died; the household has moved permanently, or travel exceeds the time the study team can be in the community, then the next child from the alternate list will be selected to participate. When the alternate child is selected, the master list will be updated to indicate which child is being replaced, and the Study Identification number of the replacement child. If additional data on the child or household is found that necessitates a change to the census list, then a Census Update Form will be completed (Section 11.2.2 Census Update Book (Form 2)).

## 8.5 Forms and Swab Management for Visits

At the end of the examination day, once all children/women on the master list of selected sentinel children/women and the alternates have been accounted for, three reconciliations will take place. First, the lab technician will be certain that each vial has a label. If a label is missing, then the form, specimen log, and vials should be matched to determine which label is missing. If more than one vial is missing a label, then the children/women may need to be re-swabbed to be certain the trachoma grade corresponds to the correct laboratory determination of infection. For the children, the number of vials must also equal the number of ocular examination forms in the tablet and specimen log, plus the five negative field control “blue air swabs.” For the women, the total number of vials must be twice the number of ocular examination forms in the tablet and specimen log, plus five negative field control “blue air swabs.” If there is a mismatch, then the vials and forms should be matched to determine what is missing. If an ocular examination form was missing or not filled in, the child/woman must be reexamined. Similarly, if a vial is missing, and there is no notation that a specimen was not taken for any reason, then the child/woman must be re-swabbed. Once the swabs, specimen log, and ocular forms are reconciled, the photographer reconciles his photo log with the number of ocular forms for women and for children to be certain that his photolog is accurate. The reconciliation form must be completed before returning from the village to indicate the survey team has matched the number of ocular forms on the tablet to the number of active swabs.

The photographer reviews his/her photo log to be certain that, for all women and those children who were selected to have digital images of the eyelid, that the photograph was taken (or a notation made as to why a photograph was not taken).

The intake clerk will compare the number of ocular exams in the tablet to the master list of selected sentinel children/women to be certain that each child/woman who presented was examined. If an ocular form is missing, but the child/woman is listed as having attended an exam, then the study team must return to the household and reconcile the difference, likely by reexamining the child/woman.

The intake clerk must account for each child/woman on the master list of selected sentinel children/women for the community, either with an ocular form, or a notation on the list (in which case there is an alternate child chosen). If the notation states the child/woman has died or permanently moved out of the community, or moved within households, then a census update form should also be present.

## 8.6 Follow-Up Visits and MDA

The baseline surveys for this trial are planned from February to October of 2013. Subsequent follow up visits are at 6, 12, 18, and 24 months from the baseline survey. In communities at risk of trachoma re-emergence at 18 months, an additional follow-up survey will be conducted at 30 months. The census lists are updated formally every year prior to the 12- and 24-month survey. The 6- and 18-month surveys will use the census lists for the previous survey when drawing new random samples of sentinel children. Women will be examined at baseline only, so follow-up visits will include children only.

The exact same procedures as stated for the baseline examination visit for children are to be followed for all subsequent examination visits. In all cases, mass treatment if indicated is to follow immediately after the surveys at baseline and 12 months. The data from the surveys prior to the start of the trial will be used to determine the number of communities “graduated” from mass treatment prior to the first MDA, and the 6-month survey will identify communities to be stopped prior to the second MDA. The third MDA will follow the final survey of the trial and will be carried out at the discretion of the National Trachoma Control program in Tanzania, if warranted.

## 8.7 Missed Visits

Missed visits, for the purpose of this trial, occur if a community cannot be seen within the window of +/- two months. The four-month window was chosen at it keeps the community visit largely during the same season as the original scheduled visit. Reasons why a community might not be able to be seen during its regularly scheduled visit include a funeral of a community leader, significant religious or country holiday, rainy weather, or a local reason advanced by community leaders. In any case, the events other than weather rarely last longer than a week, so we expect no difficulties in rescheduling visits within the window and have experienced none so far. The reason for the broad window is that other communities may have been notified of their visit schedule, and it is critical to honor those commitments, so a last minute reschedule may take up to three weeks to redo. In the previous trial, we have had no visits that are outside the window for any of the communities.

## 8.8 References

1. Thylefors B, Dawson C, Jones BR, West SK, Taylor HR. A simple system for the assessment of trachoma and its complications. Bull WHO 1987;65:477-83.

Chapter

9

# **Chapter 9: Laboratory Analyses**

## 9.1 Laboratory Processing Procedures

When the swabs arrive at Johns Hopkins International Chlamydia laboratory, they are to be immediately stored at -80°. The swabs are to be processed using a pooling strategy, within 90 days of arrival, and results reported to the Data Coordinating Center. The swabs are processed by the laboratory according to the identification code, which assures they are masked to intervention or control village status, and masked to their status as “blue air” controls. The specimens are processed according to strict protocol, outlined in the manufacturer’s kit directions, followed by the Johns Hopkins International Chlamydia Laboratory.

### 9.1.1 Testing JHU Laboratory

We will be using the APTIMA ACT commercial test for *C. trachomatis* (Gen-Probe Inc., San Diego CA). We had formerly used the Roche Amplicor qualitative PCR assay, but they have discontinued the test. The GenProbe assay has comparable sensitivity and specificity to the Amplicor assay (in our hands the comparison using ocular swabs was 99% concordant with Amplicor) [1-3] so we are not concerned for our longitudinal analyses, and we will be using this assay throughout the currently proposed trial.

For communities with active trachoma prevalence under 15%, we will institute a pooling strategy that will allow assay costs to be decreased by pooling four consecutive samples together into one test transport tube and running the assay. The pooling strategy has been shown to be both sensitive and specific compared to results that are not pooled.[4-7] Dr. Gaydos’ laboratory was the first to describe and validate pooling for Chlamydia [4] and several laboratories are now using a pooling strategy for testing Chlamydia samples when the prevalence is low.[5-7] The pooling procedures are as follows:

### 9.1.2 Pooling Algorithm

1. Upon receiving samples for testing in the laboratory, 500 µl from each of 4 specimens in GenProbe transport media tube, which contains 2.7 ml of transport media, will be placed into an empty GenProbe transport tube. This will constitute a “pool” of a volume of 2 ml. [The remaining original sample tube will contain 2.2 ml, which is enough volume remaining for two additional tests by GenProbe, as the sample pipette on the robotic processor uses 400 µl volume for the assay.
2. All pools will be subjected to testing by GenProbe *C. trachomatis* assay.
3. For pools that yield a negative result, all specimens in that pool will be considered to be negative for *C. trachomatis*.
4. For each pool that yields a positive test in the Gen-Probe assay, the original samples will be retested in order to determine which sample (s) has/have a positive result.
5. Results for the individual samples in the positive pool will each be recorded as positive or negative according to the individual re-test result.
6. In the event that the pool is positive but all individual samples are negative, the individual samples will be re-run. Any positive samples will be called positive, and any negative samples, after two runs, will be called negative.

### 9.1.3 Assay Procedure

The Gen-Probe TMA reaction replicates a specific region of the 16S rRNA from *C. trachomatis* via DNA intermediates. A unique set of primers is used for the target molecule. Detection of the rRNA amplification product sequences (amplicon) is achieved using nucleic acid hybridization. A single-stranded chemiluminescent DNA probe, which is complementary to a region of the target amplicon, is labeled with an acridinium ester molecule. The labeled DNA probe combines with amplicon to form stable RNA:DNA hybrids. The Selection Reagent differentiates hybridized from unhybridized probe, eliminating the generation of signal from unhybridized probe. During the detection step, light emitted from the labeled RNA:DNA hybrids is measured as photon signals in a luminometer, and are reported as Relative Light Units (RLU). Test results are automatically interpreted by the APTIMA Assay Software using the manufacturer’s protocol. A test result may be negative, equivocal, positive, or invalid as determined by total RLU in the detection step (Table 9.1). A test result that is outside the normal ranges is invalid and will be repeated. Equivocal and low positive test results will be repeated.

### 9.1.4 Interpretation of Assay Results

Test results are automatically interpreted by the APTIMA Assay Software using the CT protocol. A test result may be negative, equivocal, positive, or invalid as determined by total RLU in the detection step (see below). A test result may be invalid due to RLU values outside the normal expected ranges. Initial equivocal and invalid test results must be repeated. Equivocal samples are re-run and if it is equivocal a second time, the sample is labeled as negative as, in two runs, it was never positive; however, due to the nature of the assay, the possibility of an equivocal result is extremely rare and we do not expect to have equivocal retests.

**Table 9.1: Interpretation of Aptima Test Results based on Relative Light Units**

| **Test Interpretation** | **Total RLU (x1000)** |
| --- | --- |
| Negative | >0 to < 50 |
| Equivocal | 50 to < 100 |
| Positive | 100 to < 12,000 |
| Invalid | 0 or > 12,000 |

### 9.1.5 Quality Control Results and Acceptability

Positive and negative controls must be run with each assay. The negative control is labeled, “CONTROL - CT NCT” and the positive control is labeled, “CONTROL + CT PCT”. These act as controls for the target capture, amplification, and detection steps of the assay. The control labeled, “CONTROL - CT NCT” contains non-infectious GC rRNA. The APTIMA Assay Controls must produce the test results showing in Table 8.2:

Table 8.2: Results expected for the APTIMA Assay Controls

| **Control** | **Total RLU (x1000)** | **CT Result** |
| --- | --- | --- |
| CONTROL - CT NCT | >0 and < 50 | Negative |
| CONTROL + CT PCT | > 100 and < 12,000 | Positive |

The APTIMA Assay Software automatically evaluates the controls according to the above criteria and will report the Run Status as PASS if the run control criteria are met, and FAIL if the run control criteria are not met. If the Run Status is FAIL, all test results in the same run are invalid and must be repeated.

### 9.1.6. Reporting Results to Data Coordinating Center

The laboratory must report all results of sample processing to the Data Coordinating Center within 90 days of receipts of specimens. The results of the pooled runs must be reported where they are all negative, and the results of the unpooled runs, where the pooled result was positive. Each run has a positive and negative control which must also be reported. Specimens reported as equivocal must be re-run and the Data Coordinating Center will request the results of the re-runs within 1 week.

The laboratory generates a spreadsheet assigning a pool number to each pooled sample and specifying the individual specimens that comprise each pooled sample, and provides a report on the results of sample processing. After these data are received by the Data Coordinating Center, the pooled results are reviewed and individual results are assigned to each specimen. If the result of a pooled run is negative, then all four specimens in that pooled sample will be recorded as negative for *C. trachomatis*. If the result of a pooled run is positive, the individual specimens that comprise the pooled sample will be run individually, and each individual specimen will be recorded as either negative or positive for *C. trachomatis* depending on the individual test results.

## 9.2 References

Gaydos CA QT, Willis D et al. Performance of the APTIMA Combo 2 assay for the multiplex detection of Chlamydia trachomatis and Neisseria gonorrheae in female urine and endocervical swab specimens. . J Clin Microbiol 2003;41:304-9.

Masek BJ AN, Quinn N et al. Performance of three nucleic acid amplification tests for detection of Chlamydia trachomatis and Neisseria gonorrhoeae by us. J Clin Microbiol 2009;47:1663-7.

1. Schachter J CM, Willis DE et al. Vaginal swabs are the specimens of choice when screening for Chlamydia trachomatis and Neisseria gonorrhoeae: Results from a multicenter evaluation of the APTIMA assays for both infections. Sex Transmit Dis 2005;32:725-8.

Kacena KA QS, Howell MR, Madico GE, Quinn TC, Gaydos CA. Pooling urine samples for ligase chain reaction screening for genital Chlamydia trachomatis infections in asymptomatic women. J Clin Microbiol 1998;36:481-5.

Peeling RW TB, Jessamine P, Gemmill I. Pooling of urine specimens for PCR testing: a cost saving strategy for Chlamydia trachomatis control programmes. Sex TransInf 1998;74:66-70.

1. Gomes JP VS, Paulino A, Catry MA. Sensitivity evaluation of the Gen-Probe AMT-CT assay by pooling urine samples for screening of Chlamydia trachomatis urogenital infection. Int J STD AIDS 2002;13:540-2.
2. Wood BJ TM, Quinn TC, Gaydos CA. Pooling of urine specimens for the detection of Chlamydia trachomatis and Neisseria gonorrhoeae by the Becton Dickinson (BD) ProbeTec ET Assay. J STD AIDS 2001;12 Suppl2:115.

Chapter

10

# **Chapter 10: Mass Treatment Administration**

## 10.1 Mass Treatment with Azithromycin

The drug of choice for treating communities with trachoma is azithromycin, a single dose antibiotic highly effective against *C. trachomatis*. Antibiotic sufficient for this trial has been donated by Pfizer International to the National Trachoma Control program. The dosage is 20mg/kg, up to 1 gm in a single dose of either liquid (for children) or tablets (for children able to swallow tablets and adults). In Tanzania, pregnant women can be treated with azithromycin, but children under 6 months are not treated but are given topical tetracycline.

MDA in all communities will be carried out and supervised by the treatment team and a network of Community Drug Distributors (CDDs). This team is not part of the survey team, and will know the randomization assignment of the community to intervention, because they will be responsible for leaving the store of azithromycin in the communities randomized to intervention, and monitoring its use over the ensuing year. The supervisor of the treatment team has a list of all community residents, based on the latest census update, and this will be used to estimate the need for antibiotic with at least 80% coverage and for the estimated need for extra drug to be left in intervention villages. In addition, she receives the data on whether a community has decreased infection to 1% or below, or TF is less than 5% and is thus eligible to have cessation of MDA, and will inform the community leadership of the findings. It is important to note that in the intervention arm, even though infection may decrease to below 1% or TF <5%, the surveillance for newcomers and travelers is still active and treatment for that group continues as indicated by the protocol (Section 4.2 Newcomer and Traveler Treatment Intervention).

An application will be made to the Director of the Tanzania Neglected Tropical Diseases program for sufficient antibiotic to cover single doses for the expected number of communities in this trial at each mass treatment round, plus the extra doses to cover the intervention. The application covers liquid for children up to age four or so, then tablets (250 mg) for the remaining population. The supplies are brought to Kongwa by truck and stored in locked facilities at the KTP compound.

After the survey days in the village, the treatment supervisor meets with the village leaders to plan mass treatment for the community, or in the intervention communities, the newcomer and traveler treatment if the community has had MDA stopped. There are typically one to two CHWs in the community, and one to two CDDs for each balozi. In the intervention communities, the CDDs may also be CMs if they are selected for both positions. CDDs are enlisted to help with distribution, with a goal of coverage of at least 80% of children. The community will be informed about the days of mass treatment in their neighborhood. At a central location, the mass treatment team leader will set up a station with his/her mass treatment list of residents in the balozi assigned to each CDD, and as each family arrive, will provide a chit (ticket) for the medication, and check off that the persons within the family have arrived. If any family member is missing, the family is asked to make sure the member comes before the end of the day for treatment. The reasons for nonattendance at mass treatment are noted on the list in pencil. Adults who come for MDA are also screened for trichiasis by the CDD, some of whom will have had additional training on trichiasis referral during training for MDA.

Each resident has his own ticket with his ID number that is redeemed for a dose of the medication; the ticket is taken by the CDD who supervises the dosing, and the dose is written on the ticket. The ticket is matched at the end of the day to the mass treatment list to be certain that those who showed up also had an observed dose of azithromycin, and assists in the accounting for drug dispensed for the national program. All communities will be provided four days of mass treatment in their neighborhoods.

More treatment days will be scheduled depending on the tally of coverage at the end of the first set of 2 days in each balozi. For subsequent days, the CDDs will be asked to personally go and inform persons who missed treatment of the subsequent treatment day in their community, and to advise treatment. If treatment is below target, then the supervisor will schedule subsequent days, including going house to house for the community, until the coverage target has been met. Based on the experience of our team in the previous trial, coverage of 80% can be achieved with 1-3 days. CDDs and CHWs are paid a flat rate of $2/day for successful mass treatment, commensurate with other community health programs in these villages.

We monitor coverage as follows: Coverage is defined as the percentage of children ages under ten years who are observed to take azithromycin out of all children in that age group. The census data that are obtained and updated yearly prior to the survey will be the primary tool used for monitoring mass treatment coverage. The programs that are built for data entry of the census data will have a report that consists of mass treatment lists for each community, organized by balozi. These master lists are used to record observed treatment coverage for each resident of the community. The data entry of these lists is essential to monitoring coverage as the treatment is underway. As data are entered on coverage, a report can be generated calculating the coverage of children up to 10 years of age, and if coverage has not hit target, a further report can be generated listing the residents missing treatment. These lists can be used to further guide mass treatment steps as described above, and the data from these reports entered to update coverage. At the end of mass treatment, each community resident should be accounted for as having been treated, or not treated and the reason why.

After each community has completed its mass treatment, the full data set on treatment is sent to the KTP project director within a week in order to monitor the success of the treatment team in reaching coverage targets. He will review the percent coverage of the community, and if it is under target, the reasons why in the file. This will be done within three weeks of mass treatment ending so that if necessary, the treatment team can be redeployed to the community to improve coverage. To date, all communities have achieved coverage above 80%.

At baseline for this trial, we will have some communities that will have achieved their target goal of infection 1% or below and will not have further MDA. To determine these communities, we will use the survey data from the preceding closest survey. Those at 1% or less will be “graduated” from mass drug administration and will enter the trial with no plans for MDA. For communities in the intervention arm that have been stopped, the treatment supervisor will explain to the CHW that azithromycin will be solely for the newcomer/traveler treatment program. Training and quality control procedures will be carried out as described in the intervention section above. In control communities that have “graduated” from MDA, the government leadership will be informed that the community has reached target and no further treatment is needed. They will be told that we would like to continue surveys to monitor for re-emergence, and will consider reinstating MDA if infection rises to 6%. For communities that have stopped MDA, we will also offer treatment to all children who had infection during the surveys at the end of the study.

### 10.1.1 Mass Treatment Verification Procedure

Treatment verification is critical to ensuring that coverage levels have been achieved and that reported data is accurate. Treatment verification occurs after each community has completed mass treatment and is conducted by the community treatment assistant who initially treated the villages. This process consists of a random sample of five households among the households assigned to each CDD. A KTP staff treatment supervisor visits each of the five homes after mass treatment, and asks the head of the household to verify the treatment status of each member of the household. The staff member is unaware of the treatment status at the time of the visit, and this is reconciled with the Project Director. The protocol states that if there is more than 20% discordance, the CDD must account for the discrepancy or receive a reduction in remuneration.

### 10.1.2 Stopping Rule Implementation

The data from the survey on infection in the random sample of 100 children ages nine years and younger will be used to determine if the stopping rule has been met. The stopping rule is based on the prevalence of infection or TF within the sample of 100 children. We plan to stop MDA in those communities who have achieved our pre-specified goal of infection estimated at 1% or below (with an upper confidence interval of less than 6%) or TF estimated at less than 5%.

The laboratories will process the specimens from the surveys prior to trial enrollment. They will report the data, and the study statistician (Ms. Munoz) will review each community’s data for evidence of infection and TF. The results—stop treatment or do not stop treatment—will be transmitted to the Treatment Team supervisor, and s/he will acknowledge receipt and place the decision document with the results in the file for each community. When it is time for mass treatment of the community, the treatment supervisor will review the decision document and plan accordingly.

In the data entry programs for entering mass treatment coverage, there is an “opt out” button for each community in the entry programs. Only the treatment supervisor can enter into that button with a password. A code will be present on the decision document. The supervisor will enter in the program that mass treatment was not carried out, and the reason was the result of the stopping rule; it will then prompt for the decision code, which must be entered to be valid.

Once a community has been stopped, it will not receive mass treatment again until after the third survey, unless the 6 or 18 month survey show infection has climbed back to 6% in which case mass treatment will be reinstituted at the next round. In this way, we are able to determine if, or with what rapidity, infection re-emerges.

## 10.2 References

1. Biebesheimer JB, House J, Hong KC, et al. Complete local elimination of infectious trachoma from severely affected communities after six biannual mass azithromycin distributions. Ophthalmology 2009;116:2047-50.
2. West SK, Munoz B, Mkocha H, Gaydos C, Quinn T. Number of Years of Annual Mass Treatment with Azithromycin Needed to Control Trachoma in Hyper-endemic Communities in Tanzania. . J Infect Dis 2009;204:268-73.
3. Schachter J, West SK, Mabey D, et al. Azithromycin in control of trachoma. Lancet 1999;354:630-5.
4. West SK, West ES, Alemayehu W, et al. Single-dose azithromycin prevents trichiasis recurrence following surgery: randomized trial in Ethiopia. ArchOphthalmol 2006;124:309-14.
5. Gaynor BD, Holbrook KA, Whitcher JP, et al. Community treatment with azithromycin for trachoma is not associated with antibiotic resistance in Streptococcus pneumoniae at 1 year. BrJOphthalmol 2003;87:147-8.

Chapter

11

# **Chapter 11: Study Instruments**

## 11.1 Overview

The data to be collected for this study consists of household information, and clinical and laboratory information on study participants. In addition, data are collected on the mass treatment intervention, and details of the study process. In the next section, we describe the forms that are used to collect these data. These forms can be found in MOP Appendix 1: General Study Forms.

## 11.2 Census Forms

Description of how the census is to be conducted, and appropriate numbering of doorways is described in Section 7.2 Baseline Census. A household is defined as a unique doorway belonging to a family. The definition of resident in the household means a person who has slept in this household for at least three month in the past (or if age less than 3 months, was born into the household) or who intends to reside with the family for the next six months. In this section, details are provided on filling in the Baseline and Update Census forms. This section should be used with the forms in MOP Appendix 1: General Study Forms.

Note that 48 of the 52 villages have been followed for the last five years, and so have already had a complete census update one year prior to the enrollment. Thus, the forms below are forms for changing the census.

The census form is used to collect information on each household in the communities randomized to this trial. It is used both to create the census at the baseline visit, as well as to add new households at later follow-up visits. It is the central document that provides information for the following:

- Selection of households with eligible children in the community
- Survey list for ocular exam visit
- Resident information for mass treatment
- Environmental factors at the household level that may be important for trachoma
- Data to help physically locate the households

### 11.2.1 Add New Household to Census Form (Form 1)

The following information is recorded on the Add New Household to Census (Form 1):

- **Village ID (VID**):
  - Insert the four digit village id number.
- **Village Name:**
  - Write in the name of the village
- **MTAA# :**
  - Use this entry to identify the mtaa.
- **Balozi:**
  - Write in the name of the balozi leader.
- **Name of Head of Household:**
  - Write in the name of the head of the household. This may be provided by the neighborhood leader or another neighbor if the head or another adult in the family is not home.
- **Household Number**:
  - To ensure the household ID is unique, the KTP office provides a list of 3 digit unique numbers to each census taker. Each time a new house is identified the first available number in the list is assigned to the house and cross out from the list.
  - Use this three digit number to uniquely identify a household; this number is also written on the door of the household.
- **Census Date:**
  - Insert the date in which the household census was completed (or the date at which census gathering had to be stopped and no information was provided). Use DD/MM/YY format.
- **(1) Is there an adult home to answer questions**?
  - If yes, mark “yes.” If not, the census taker should determine when would be a better time to return to get the information. If the family is traveling and not returning during the time of the census, then the census taker must indicate the reason for being unable to census. If there is no information on the family, s/he MUST return at least three times at different times over the course of the village census to try and catch an adult at home. If the census taker is unsuccessful, then mark “no” unable to census and provide a reason why. This household will not be chosen as a source of sentinel children, as detailed information will not be available on the children, but census information may be provided at the time of mass treatment, if the family returns for that activity.
- **(2) What village did this household live in previously**?
  - Write in where the household came from. This is especially important for new households moving into the village during follow-up visits.
- **KTP Supervisor Initials:**
  - Insert the initials of the census taker for this household. Each census taker should have a unique set of initials, including a number if there is more than one person with the same initials.
- **Date:**
  - Insert the date in which the KTP personnel completed questions 3-6. During the baseline visit, this information is likely to be captured at the same time as the rest of the census information. However, at follow-up visits in the intervention villages, the KTP supervisor will come out at a later date. Use DD/MM/YY format.
- **(3) How many years of education have the head of household completed?**
  - Ask the question of the adult, and make sure that the answer is years completed, not including starting but not completed. Write in the number of years. If the answer is a grade, translate that into years completed. If the adult does not know, write letter “X” for unknown.
- **(4) How far away is the nearest water source during the dry season?**
  - We will measure the distance to water by asking how long it takes to walk one way directly to the nearest source of water. Time will be measured relative to the time it takes to do common tasks, such as boil water, cook ugali, or other tasks. Once the persons can provide an estimate, code as either less than 30 minutes, 30 minutes to one hour, or more than one hour.
- **(5) Does this household own a bicycle?**
  - This refers to any member of the household as listed on the census. It does not refer to other family members in another house who may own a bicycle. The census taker will record the response: ‘yes’ or ‘no.’
- **(6) OBSERVE: Does this household have a latrine:**
  - The census taker will look in the compound or area around the house for a latrine. If one is not visible, the census taker will ask the interviewee if the household has a latrine. If yes, the team member will ask to see it. If it is within 60 feet (20m) of the house, it will be counted as a latrine for that household.
- **Household List:**
  - Note that to the left of each name slot is a pre-printed number in sequential order. This two digit code is the unique person code for each person in the household. Use it when needed to identify the mothers of children as described below.
- **Name:**
  - List the names of all persons who reside in this household, as defined above. Start with the head of household. List the first name, last name, and an optional place for alias if needed.
- **Sex:**
  - Circle the sex of the person.
  - Since the forms are translated to Swahili, the responses include “ME” for male and “KE” for female
- **Date of Birth:**
  - If it is possible to obtain a date of birth, record the date of birth: use DD/MM/YY format. If only the year is known, fill in “x” for the unknown numbers. Use a vaccination card or MCH card if available to get the birth date of children, and a voting card or other piece of ID for the adults if available. If the entire date is unknown, then skip to the column labeled “age.”
- **Age:**
  - Use this column if no birth date, or at least year of birth, can be obtained. Use an events calendar specific for each region to estimate the age of the person. If necessary, have other family members assist in determining the age. Avoid having a consensus using round numbers or last digits with 5’s as it indicates digit preference; try to get the person and/or family members to be as precise as possible. For children age less than one, insert a zero for years and indicate years and months if possible.
- **Mother:**
  - For each child age less than ten years, insert the two digit person code for the primary caretaker of the child. This will help identify the child in subsequent surveys.

### 11.2.2 Census Update Book (Form 2)

At the 12 month and 24 month follow-up visits, a census update team will be sent to each village to update the database’s census so that it contains an accurate representation of the households in the village. The census team will be given the Census Update Book (Form 2), which has the residents we know about printed out, one household per page. The census team will go to each household, and mark whether the residents are absent or present. If absent, the residents will be marked as either deceased (and a date of death given if known), temporarily absent if the resident is only away at the time of the census and is expected to continue living in the household, or permanently absent if the resident has left the household for good. Residents that are new to the household since the last census will be added to the Census Update Book (Form 2). New households to the village that are found during the census update phase will have an Add New Household to Census (Form 1) completed. For new households that moved within the village, the household will retain their household ID number. In this instance, the census taker will confirm the information below, but cross out the balozi leader name and record the name of new balozi leader.

For current residents, the following information should be completed:

- **Date of Visit:**
  - Enter the date of the census update: use DD/MM/YYYY format.
- **Status:**
  - For each resident listed, indicate whether they are absent or present. If absent, check the checkbox either for deceased, permanently absent, or temporarily absent. If present, check the checkbox for present.
- **Date of Death:**
  - If the resident has died, indicate the date of death if known: use DD/MM/YYYY format.
- **New HID:**

If the resident has moved to another household within the same village, find the 3 digit household number of their new home. This data can be found by looking at the other pages of the Census Update Book (Form 2).

- Cell Phone Number
  - A cell phone number for the household will be part of the update

For new residents found in the household during the census update, complete Section II of the Census Update Book (Form 2).

- **Name:**
  - List the names of all persons who reside in this household, as defined above. Start with the head of household. List the first name, last name, and an optional place for alias if needed.
- **Sex:**
  - Circle the sex of the person.
- **Date of Birth:**
  - If it is possible to obtain a date of birth, record the date of birth: use DD/MM/YY format. If only the year is known, fill in “x” for the unknown numbers. Use a vaccination card or MCH card if available to get the birthdates of children, and a voting card or other piece of ID for the adults if available. If the entire date is unknown, then skip to the column labeled “age.”
- **Age:**
  - Use this column if no birth date is available, or at least year of birth, can be obtained. Use an events calendar specific for each region to estimate the age of the person. If necessary, have other family members assist in determining the age. Avoid having a consensus using round numbers or last digits with 5’s as it indicates digit preference; try to get the person and/or family members to be as precise as possible. For children age less than one, insert a zero for years and indicate years and months if possible.
- **Mother:**
  - For each child age less than ten years, insert the two digit person code for the primary caretaker of the child. This will help identify the child in subsequent surveys.
- **Old HID+PersonN:**
  - If the new resident moved to this household from another household in the same village, enter the 3 digit household number and the 2 digit person number that the new resident had in their old household. This data can be found by looking at the other pages of the Census Update Book (Form 2).
- **Present:**
  - Check the checkbox indicating that the new resident is present in the household.

If the entire household has departed the village, then note that the household has left permanently by checking the checkboxes for permanently absent for each person. There should be tangible evidence of permanent re-location and not just traveling. Temporary absences of households of less than one year should not be changed in the census unless the neighbors/village leaders know they have gone for good.

## **11.3** **Cooking Fire Questionnaire**

The purpose of this questionnaire is to collect information about the use of and exposure to cooking stoves/fires by children and the women who have been selected for the survey. There are two parts: one for the census team to administer, who are out at the house curing census. The other one, on exposure to cooking fires, will be administered to women at the time of the survey. .

11.3.1 Cooking Fire Questionnaire for Households

Explain to the household that the next set of questions is to better understand exposure of household members to cooking fires, and possible eye health effects. Proceed with the following:

- **Time started:**
- Enter the time the interviewer starts the questionnaire: use 00:00 format (24-hour clock).
- **Date:**
- Enter the date: use DD/MM/YYYY format.
- **Interviewer initials:**
- Enter the interviewer’s initials.
- **HID:**
- Enter the four-digit village number and three-digit household ID number.
- **Respondent CID:**
- Enter the CID of the respondent. Be certain that it matches the CID on the census form for the respondent.
- **Name:**
- Enter the name of the respondent and make sure it matches the name on the census form. .
- **Number of people living in the house:**
- Enter the number of people living in the house, as noted on the census form.
- **Number of rooms in the house:**
  - Please ask the respondent if you can see the interior of the house to count the number and types of rooms. A room is defined as having four walls and a doorway that distinguishes it from other rooms. A room can have multiple functions. If this is a multiple family dwelling, there should be a unique doorway for the family’s suite of rooms, even if there are other common rooms-only use the suite of rooms for that family. Enter the number of rooms in the house.
- **Room info:**
- For each room you have counted, ask the following as to all the things that it could be used for, check ALL of the choices that apply:
- Living (sitting room)
- Eating (where food is eaten)
- Cooking (only if a cooking fire is in this room-if food is just served or other ingredients are added, this is not a cooking room)
- Sleeping (at least one family member sleeps in the room)
- Other (specify)
- **OBSERVE: What is the roof made of?**
- Check the choice that applies:
- Tin (the entire roof is tin)
- Partially tin (there are other materials besides tin)
- Mud (mud and or wood only)
- Other (specify)
- **OBSERVE: What is the wall made of?**
- Check the choice that applies:
- Mud brick ( may have plaster coating)
- Wood and mud wattle
- Concrete/concrete brisk
- Other (specify)
- **OBSERVE: What type of stove does the family use?**
- Check the choice that applies:
- Ceramic
- Metal
- Stone-this is just a pile of stones or bricks that hold a pot
- Gas
- Other (specify)
- **OBSERVE: Where is the current cooking fire located?**
- Check the choice that applies:
- Outside in open air
- In a separate room not connected by door to main house with added ventilation (windows/holes in roof)
- In a separate room not connected by door to main house without added ventilation (only the door)
- Inside the house with added ventilation (windows/holes in roof)
- Inside the house without added ventilation (only the door)
- If the current cooking fire is inside the house, then ask for and enter the number of hours per day the family is in the room with the fire lit. This means at least one member of the family. If the room is used a lot, and especially for sleeping then you may need to ask, who spends most of the time in this room, including sleeping and ask how many hours a day. It might help to break it into night time hours an daylight hours for this person.
- **OBSERVE: Does the family sleep on a bed (raised off of the floor)?**
- Check the choice that applies:
- Yes, all of them
- Only some of them
- None of them
- **Cell phones:**
- Enter the number of cell phones owned by members of the house.
- **Bicycles:**
- Enter the number of bicycles owned by members of the house.
- **Location each child sleeps:**
- Review the number of children under age ten that are listed on the census form. For each child under 10 who lives in the house, enter the CID and ask in what room that child sleeps. Check the choice that applies:
- In a SEPARATE ROOM from the fire, and sleeping room WITH ventilation (window/roof hole)
- In a SEPARATE ROOM from the fire and sleeping room WITHOUT ventilation (window/roof hole)
- In the SAME ROOM as the fire WITH ventilation (window/roof hole)
- In the SAME ROOM as the fire WITHOUT ventilation (window/roof hole)
- This child does not sleep in this house
- **Number of children:**
- Enter the number of children under 10 living in the house.
- **Which children are in the fire room during cooking?**
- Check the choice that applies:
- None of the children
- All of the children
- Some of the children (list CIDs)
- Not applicable: there is no cooking fire room.
- **Do any of the children have any lung diseases/problems breathing?**
- Check the choice that applies:
- None of the children
- All of the children
- Some of the children (list CIDs)

**Who is the main cook in the household?**

Ask the respondent of the household members, who does most of the cooking, and enter the ID here. If the main cook is in another household, check the box of not in the household census

How often in a typical month do your eyes hurt while cooking.

Ask this question directly of the main cook. If the main cook is not there, check “unknown”

Other than never or every time she cooks, the other choices are more than once a week (4 times a month) or less than once per week.

**11.3.2 Sentinel Women Cooking Habits**

Explain that the purpose of this questionnaire is to collect information on frequency of cooking and exposure to cooking fires, and the relationship to eye irritation.

Place the label on the box for the CID. Write the name of the respondent in the appropriate line.

- **How many times a day do you typically cook?**
- By this we mean discreet times. She might be cooking and washing while something boils, then get back to cooking, but we mean for each main meal that requires heating a fire or a stove. Enter the number of times a day she cooks.
- **How many meals does your household have in a day?**
- Enter the number of meals the household has in a day.
- If the respondent cooks fewer times per day than the household eats, probe as to why this is so. check the main reason that applies:
- Lack of time
- Fuel cost
- Not needed: this could be because one main meal is made and it is used throughout the day although people eat multiple times.
- Not main cook
- Other (specify)

The next set of questions asks where she cooks during wet season and dry season. You can either ask them by season or by cooking indoors or outdoors, whatever is easier

- **How often do you cook outdoors (and not in a room) in the WET season?**
- Outdoors means not in any room even a cooking fire room that may be outside and attached to the house. Check the choice that applies:
- Never
- Once a week
- 3-4 times per week
- 5-6 times per week
- Every day
- **How often do you cook outdoors (and not in a room) in the DRY season?**
- This is the same question but in the DRY season. This may be more common because there is less chance that rain will drown the fire. Check the choice that applies:
- Never
- Once a week
- 3-4 times per week
- 5-6 times per week
- Every day
- **How often do you cook in a room in the WET season?**
- In a room means cooking in any room, even if it does not have a roof but just walls, or just a roof with no walls-any shelter from the outside. This answer might be more common for the wet season, as the room might protect against rain. Check the choice that applies:
- Never
- Once a week
- 3-4 times per week
- 5-6 times per week
- Every day
- **How often do you cook in a room in the DRY season?**
- Check the choice that applies:
- Never
- Once a week
- 3-4 times per week
- 5-6 times per week
- Every day
- **What is the fuel mainly used in your household for cooking?**
- Check the choice that applies:
- Wood
- Charcoal
- Charcoal and kerosene
- Other (specify)
- **Main cook:**
- **Ask at what age did you start to cook?**
- By this we mean at what age she started to cook over a fire. Manuy girls point ugali but do not cook. Ask specifically about cooking over a fire. If she does not know what age, attempt to at least pin point an age range, by reference to school grade or how little-had she stopped growing, was she married and at what age was she married, etc. Enter age.
- **Ask how many hours per day do you spend cooking meals over a cooking fire or stove total.**  Add that to the line. Then ask if this is for more than one meal, and if so, how many. Either enter 1 or higher number. Make sure the number does not exceed more than the answer to Question 1, or reconcile the answers

## 11.4 Ocular Exam Forms

The survey teams are given a list of the children selected, their corresponding ocular exams and labels to be used for the specimen collection and photograph inventory. The survey team is also given a list of the women who have been selected for the baseline assessment of scarring.

### 11.4.1 Survey List (Sentinel List)

This report lists the sentinel children for the exam visit. The children are grouped by their

balozi leaders to make it easier to locate their names. The first column of the report has two indicators: a * indicates that this child is not on the alternate list, and a “P” indicates that the child has been randomly selected to have photos taken of their eye. This report helps the exam team organize and coordinate the exams. The intake clerk uses the list to keep track of who has come for a visit and who has not, and if not, the reason why. For the baseline survey, the list will include the women who have been randomly selected for survey.

### 11.4.2 Ocular Exam Form (Form 3)

The ocular exam form is collected via the Samsung tablet. The directions for use of the tablet are in Appendix 3, and the section on the ocular form is reproduced here. The data are collected by a data recorder, based on the ocular examination and the collection of swab and photography information. The following are the instructions for the data recorder:

- Turn the tablet on, if it is not already on, enter your password, and get to the home screen. Follow the next set of instructions to get to the ocular form.
- Tap the “ODK Collect” icon from the home screen.
- You will see a menu of options that looks like the following (see image one below). Tap the “Fill Blank Form” button. If you tap the wrong button, press the back key to go back to the ODK Collect screen. If you press the home key, you will need to tap “ODK Collect” again, and it will take you to the screen that has the wrong form and you will still need to press the back key again.


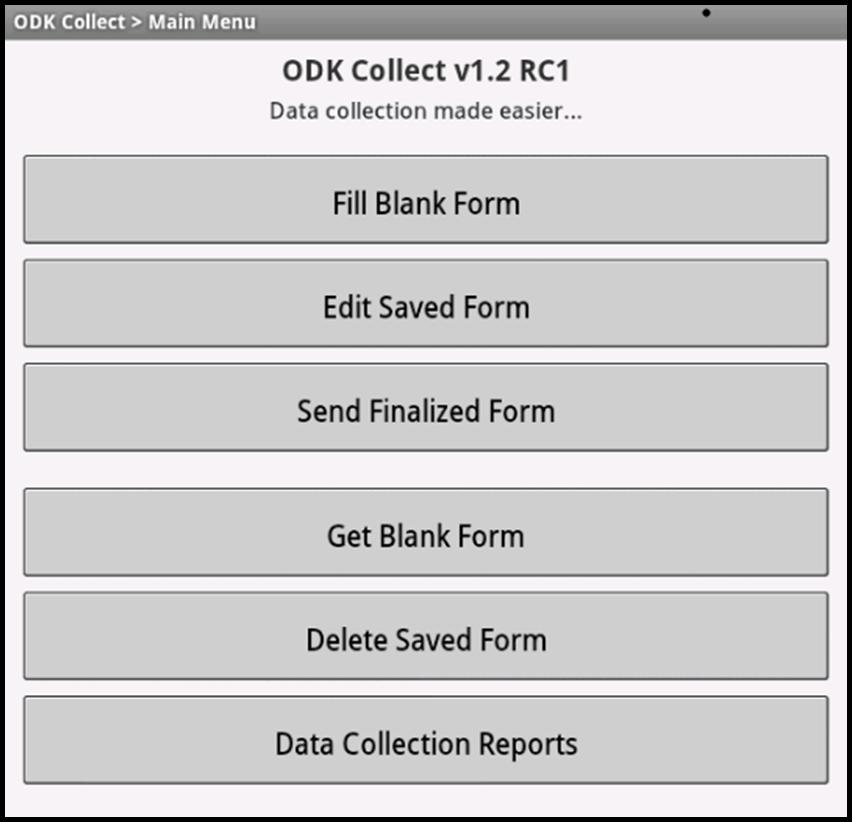


- After you have tapped the “Fill blank form” button, tap to select the appropriate ocular form. This means you need to know what community “number” you are in and what visit number if there is more than one option for a form. For this example, we will use *Community number 0101 and visit 1*. Once you tap the ocular form you want, you will see a window pop up that indicates the form is loading. Wait until it loads, which may take a few seconds.
- At the start of the form, you will see a message that says “You are at the start of Ocular Exam [Community number] [Visit]. Swipe the screen as shown to begin.” Make sure you are using the form for the correct community and correct visit number.
  - As our example, the screen should read “You are at the start of Ocular Exam Community 0101 visit 01” If not, press the back key, and a window will pop up and you can then tap “Ignore changes” in the window. This will tell the tablet not to save anything you have done in that wrong community, even if you have not entered any information yet.
- Once you have checked that you are using the correct form, swipe your finger across the screen to the left to move to the next prompt (note that if you want to go back to a previous prompt, swipe your finger across the screen to the right-it is just like moving a page in a book).
- You are now in the form itself. The first thing the form needs is an identification number for the child who is being examined. This number is on the cheti brought in by the family, or on the labels that are accompanying the child to the exam. The tablet already knows who needs to be examined, so you simply need to tap the correct identification number and name. Follow the instructions below:
  - Enter the CID by tapping on the grey answer bar and selecting the correct ID/name (see image 2 and 3 below). To scroll through the list of IDs/names, swipe your finger up or down on the screen until you get the right number. Then tap that number.


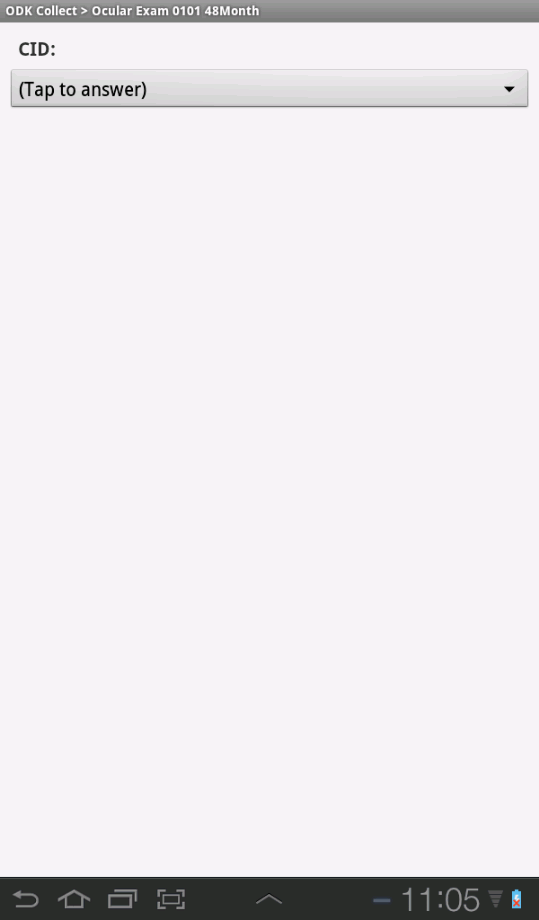


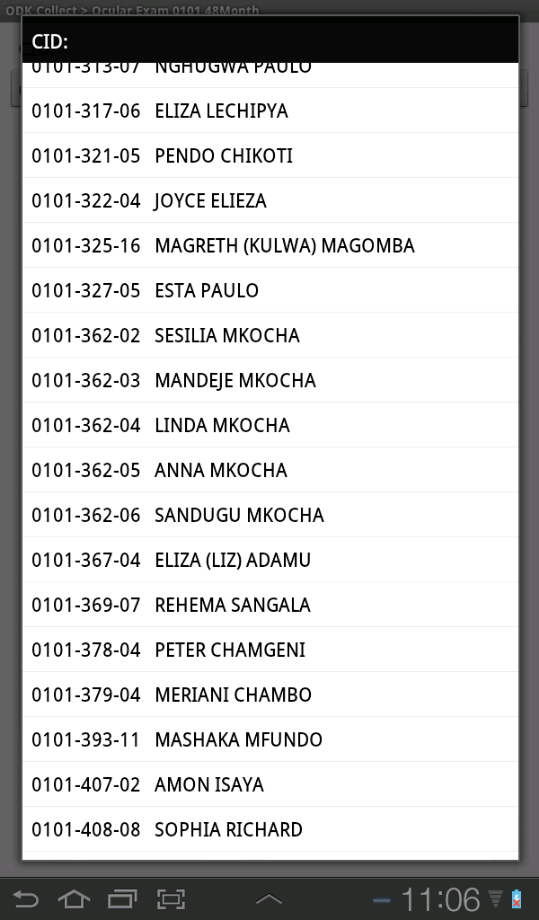


- - For this example, pretend we are examining child number 010136204, Linda Mkocha. You can see that there are five children in that family to choose from. Now tap in the the correct name and CID for Linda Mkocha.
  - After you have selected the correct CID, swipe your finger across the screen to the left (“turn the page) to go to the next prompt.
  - If you do not select a CID, you will get a message that says “An answer is required!” You will not be able to go to the next question until you select a CID. If the child has no labels or ID number, at the time of the survey, you will need to go back to the front registration area to get the correct CID.
  - Swipe the screen to the left to go to the next prompt.
- The next prompt asks you to reenter the last five digits of the CID. The community number is already entered, so you just need to enter the last 5 digits of the CID. This is to verify that you are entering the data for the right person. For this example, we are examining child 010136204-you only need to enter 36204.
  - There should be a (virtual) keyboard at the bottom of the screen. If the keyboard is not there, tap the box where you enter the CID, and the keyboard will show up.
  - To switch from the alphabet keys to number keys, tap the “123” key at the bottom left corner of the keyboard. (see image below). Also, make sure the button above it says “1/3.” If it says “2/3” or “3/3” just tap that button until the key says “1/3.”
  - If you make a mistake in entering the CID, you can delete your entry by touching the arrow key at the top right corner. Then enter the last 5 digits and swipe your finger across the screen to the left to go to the next prompt.
  - If you enter the CID that does not match what you previously entered, you will get a message that says “Sorry your response is invalid!” You will need to enter the correct CID before moving to the next prompt.
    - If the first entry, where you selected the child, was correct and the second entry was wrong, go back to the key board and enter it again.
    - If the first entry was wrong (perhaps the wrong child was identified), go all the way back to the first CID entry page by swiping the tablet back to that page. Tap on the grey bar of the wrong selection and that will unselect the ID that was wrong. Then tap on the correct ID. Swipe and now enter the 5 digits of the new CID in the verification page as shown above.
  - Exercise: Now tap in 36205, and then delete your entry using the arrow key in the right hand corner. Tap in 36205 again and swipe your finger to turn the page, just so you can see the error message. Correct the error and enter in the correct ID now. Do not proceed to the next set of questions until you have mastered selecting the CID from the list and then entering the last five digits, and when you feel comfortable using the keyboard.

The next set of questions asks about the face observations. You must enter the data that the receptionist has observed. You can see the questions as in the image below:


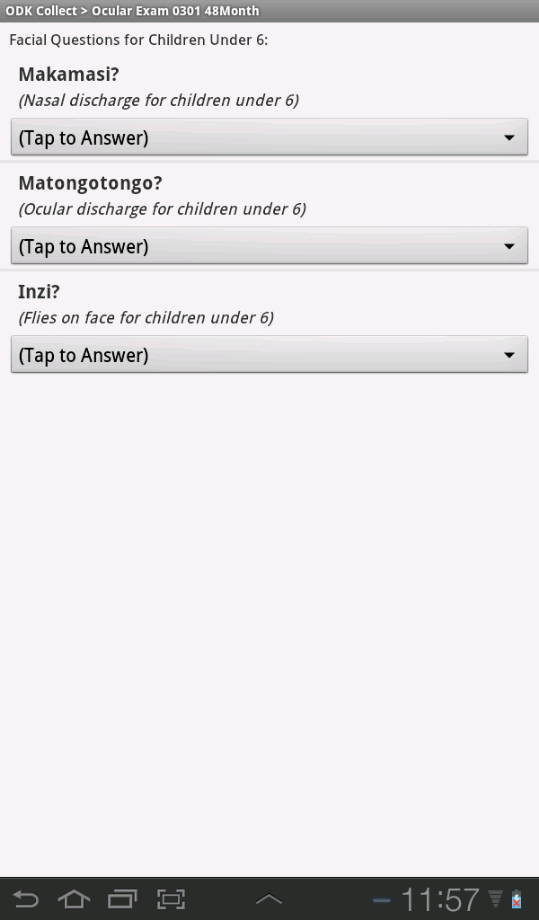


If you tap the question, the screen shows answers that you can choose from, either “yes” or “no” or “don’t know/can’t observe.” Answer the facial questions by tapping on the grey answer bar that is the answer you choose- see the image below for the possible answers to “Makamasi?” Tap the answer you want.


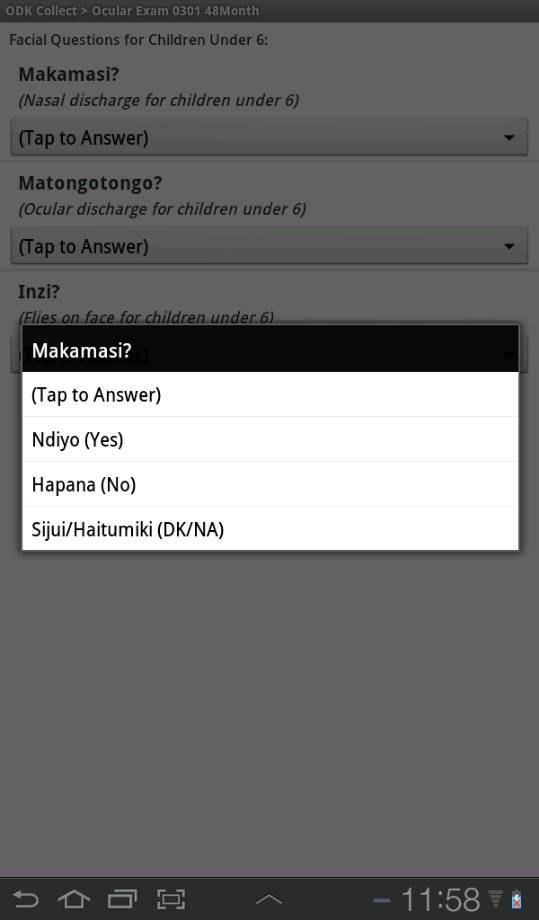


- - **Double check your answers**, then swipe your finger across the screen to move to the next prompt. If you forget to answer a question, you will get a message that says “An answer is required!” You will not be able to go to the next prompt until you answer all three questions.
  - Swipe to get to the next screen for trachoma grading.
- Trachoma assessment. The trachoma grader will call out the grades of the left eye then the right eye for TF, TI, TS, TT, CO. Each grade must have an answer of “yes” or “no” or “can’t grade.” Answer the trachoma grade questions for the left eye by tapping on the grey answer bars and selecting the correct answer. **Double check your answers**, then swipe your finger across the screen to move to the next prompt. If you forget to answer a question, you will get a message that says “An answer is required!” You will not be able to go to the next prompt until you put in a grade for all five signs. Complete the same steps as above for the trachoma grade for the right eye. It is **very important to double check your answers** before moving to the next screen.
- The next screen asks you if this child was chosen for field control. Answer the question by tapping on the grey answer bars and selecting the correct answer. If you answer “no/hapana,” you will move on to the photograph questions. If you answer “yes/ndiyo,” then a field control sample of this child will be required. For example purposes, this child, Linda Mkocha, will need a field control. This means the survey team will take an extra swab of the air around the child, and give it a number that is especially assigned. We need to enter the data on that number, which you will do by scanning the code on the label affixed to the tube. You will be prompted to scan the barcode of the field control label. To scan a bar code, do the following:
  - You will need the swab tube stand (white box with two swab sticks sticking out if it) for this. Take the field control tube which has the label and place it on the two sticks so that the label reads from left to right, facing you.
  - Press the “Get Barcode” button on the tablet and turn the tablet to its side and position the tablet so that you can see that the barcode is fully contained inside the tablet viewfinder on the screen and the red line runs through the barcode. Once you have the tablet positioned, hold still so the scanner can focus on the barcode (see image below). Once the scanner is focused, it will scan the barcode and a green line will appear in the viewfinder.


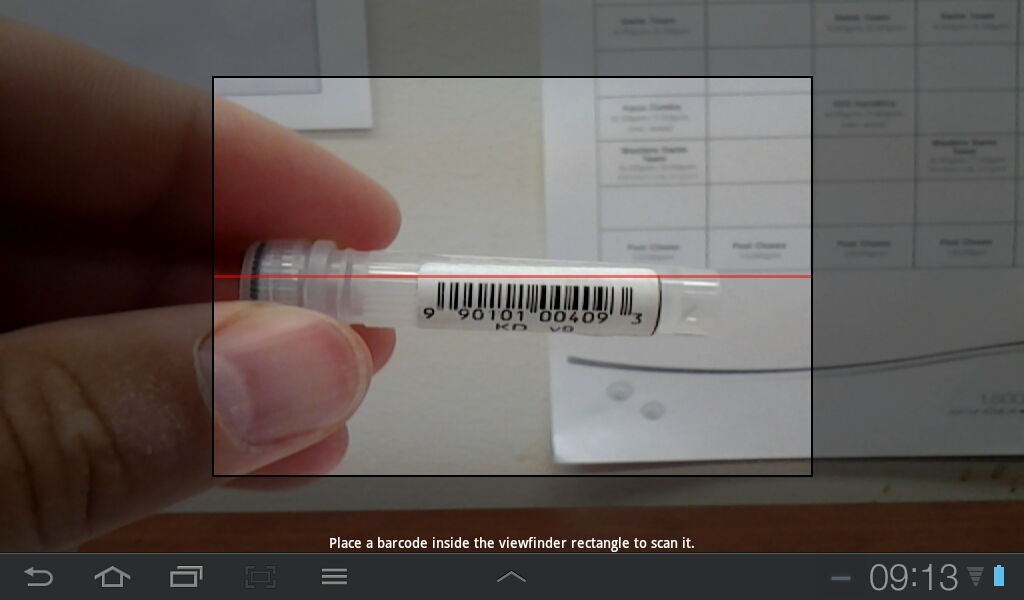


- - If you have trouble taking a scan, try moving the tablet forward or back, or try repositioning get better lighting.
  - Once the barcode scans, **make sure that the number that was scanned matches the number of the swab tube**. Sometimes a smudge on the label will read the bar code numbers wrong and this generates a mistake. If the numbers do not match up, press the “Replace Barcode” button and try again. Once you get the correct barcode, swipe your finger across the screen and move to the next question. If you absolutely cannot read a barcode after five attempts, skip this step by tapping the back key and swiping your finger across the screen to move to the next question. Make a note on the swab log that the bar code could not be imaged.
- The next screen shows all the available labels that were assigned for field controls. We are asking that you verify the bar code entry. Enter the field control label CID used for this specimen by tapping the grey answer bar and selecting the correct CID. To scroll through the list of CIDs, swipe your finger up or down on the screen. If you select the incorrect CID, tap on the grey bar again to “unselect” your choice, and then select the correct CID. After you have selected the correct field control label CID, swipe your finger across the screen to go to the next prompt. If you do not select a field control label CID, you will get a message that says “An answer is required!” You will not be able to go to the next question until you select a field control label CID.
- The next screen asks you if the child was chosen for photographs. Answer the question by tapping on the grey answer bar and selecting the correct answer. If you answer “no/hapana,” you will move on to the swab questions. If you answer “yes/ndiyo,” you will be asked if the photo was successfully taken. If you answer “no/hapana,” you will need to enter an explanation, from the photographer. If you answer “yes/ndiyo,” you will be prompted to enter the beginning and ending photo slots. Ask the photographer to look at his photolog and give you the entries for the photographs. Enter the first number in the first box and the last number in the second box. Then swipe to the next screen.
- The next screen asks you if a swab was taken. We attempt to take a swab on every person in the survey, so this is a critical set of information. Answer the question by tapping on the grey bar and selecting the correct answer. If you answer “no/hapana,” you will need to enter an explanation from the trachoma grader. Note that no swab is taken for the women to be examined. If you answer “yes/ndiyo,” you will be prompted to scan the barcode of the swab label. Follow the directions above for scanning the barcode from this sample. After you have scanned the barcode, move to the next screen.
- This is the end of the ocular form. You now have to save the form, which saves the information.
- Before you do, change the name of the file by adding the CID to the end of the file name. You can do this by tapping your finger at the end of the file name, so that the cursor shows up there. The keyboard will automatically pop up


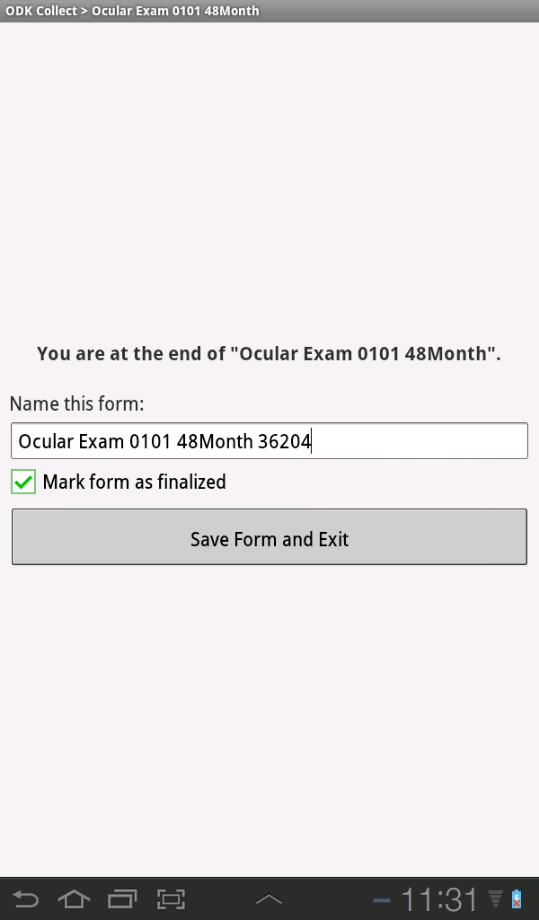


- Remember to change the keyboard to numeric by tapping the 123/ABC key and you can then enter the CID. After you have changed the file name, then tap the “Save Form and Exit” button (see above).
  - If you forget to add the CID to the file name before you save, it’s ok. You do not have to start over or reenter everything. It will just be a little harder to locate the file in case you need to make edits to the form.
- If you start a new form by mistake, press the back key and tap “Ignore changes” in the window that pops up.

Editing ocular form

- If you have saved the form, and must go back into the tablet to make a change, please follow the instructions below. THIS SHOULD NOT HAPPEN if you have been very careful throughout the data entry process. Sometimes, though, there may have been an error in the tube you were handed, or another issue. To make changes to an ocular form you have already saved, to the following:
  - Go to the home page and push the ODK Collect button.
  - Tap the “Edit Saved Form” button from the ODK Collect menu of options.
  - The saved forms are listed in order of the dates/times the files were last modified, with the oldest files at the top and the most recent files at the bottom. So, if you want to make changes to the form for the last child examined, the file will be at the bottom of the list. You may have to swipe your finger up the screen to scroll down to the bottom of the list.
  - Select the appropriate ocular form. You will see a window pop up that indicates the form is loading.
  - You will then see all of the entries for that ocular form. You can swipe your finger up and down the screen to scroll through the list of entries.
  - Select the entry that you want to edit. You can then make your changes.
  - After you are finished, tap the back key and select “Save Changes” in the window that pops up. If you made a mistake and do not want to save your changes, select “Ignore Changes.”
  - This takes you back to the ODK Collect menu. If you have more changes to make, tap the “Edit Saved Form” button from the menu and repeat the steps above.

Deleting ocular form

- If you have saved the form, and must go back into the tablet to delete it, please follow the instructions below. THIS SHOULD NOT HAPPEN if you have been very careful throughout the data entry process. Sometimes, though, there may have been an error in that the child who the team thought they were surveying was not the correct child, and you have to delete the information and start again. YOU MUST OBTAIN PERMISSION TO DELETE A FORM FROM THE HEAD OF THE SURVEY TEAM. The head of the survey team will make a note and inform Mr. Mkocha that records were deleted in the field. To delete an ocular form you have already saved, to the following:
  - Go to the home page and push the ODK collect button
  - To delete an ocular form you have already saved, tap the “Delete Saved Form” button from the ODK Collect menu of options.
  - There are two tabs at the top of the screen. You must select the “Saved Forms” tab. DO NOT SELECT THE “BLANK FORMS” tab.
  - The saved forms are listed in alphabetical order. Find the form you want to delete, and tap on the check box to the right of the form name. **Double check that you are selecting the correct form.** Once you are sure you have selected the correct form to delete, tap the grey “Delete Selected” button at the bottom of the screen. A window will pop up that says “Delete [#] form(s)?”Usually, you will want to delete only one form, so this should be a one. However, Double **check that the number of forms listed in this window matches the number of forms you want to delete.** If the number of forms is not correct or you do not want to delete any, tap “Do Not Delete.” If you are sure you want to delete, tap “Delete Forms.”
- When you are finished deleting, tap the back key. This will take you back to the ODK Collect menu.
- **If you delete something using the “Delete Saved Form” button, you will delete the entire form and you will not be able to get that information back. If you are unsure about what you are doing, do not delete anything! Make a note of whatever you are unsure of, and discuss with Mr. Mkocha back at the office.**

### 11.4.3 Photo Log Book (Form 4)

The purpose of the photo log book is to keep a record of the photograph images taken as backup in case there is an error in the order the images are taken, or the ID photo is missing. The photo log book consists of sequentially numbered columns. For each person to be photographed, the ID label with the form is placed in the next available slot, and the image is taken of that identification number. This link enables the clinical grade to be associated with the photograph grade. Then the photographer takes two images of the upper eye lid as described in Section 8.3.2.2 Photograph Procedure. S/he reviews the images, and if they are acceptable, crosses off the next two slots after the ID label in the photo log. If one or both is not acceptable, then one or more are deleted and new images taken until there are at least two that are acceptable for transmittal. There should be an “x” for as many images as are going to be transmitted.

At the end of the survey, the photographer burns the images onto a CD and sends the CD to Dr. West, with a copy of the photo log for that survey period. Note that at baseline, there will be images from the children and images from both eyes of the women.

- **CID Label**
  - This is the location on the form where the CID label is affixed.
- **Initials**
  - This is the location on the form for the initials of the photographer.
- **Date Photos Taken**
  - The date that the photographic images were taken: use DD/MM/YYYY format.
- **Burned to CD**
  - This box is checked to indicate that at least two images are acceptable for transmittal for grading. There should be an “x” for as many images as are going to be transmitted.

### 11.4.4 Swab Log Book (Form 5)

The purpose of the Swab Log Book is to keep a record of the ocular swabs taken as backup in case there is an error in the ordering of swabs and to collect details on the swab collection process. Like the Photo Log Book (Form 4), the Swab Log Book (Form 5) consists of sequentially numbered columns. A copy of the swab log is sent when the specimens in the log are sent to JHU. The data are not entered into the computer, but used in case reconciliation of the shipment is required. A file of lab specimens taken is already available from the ocular form tablet entry and can be used to construct the work sheet for the laboratory.

- **Box number**
  - The number of the box where the swab is stored is inserted here.
- **Lab Tech initials**
  - The initials of the lab tech analyzing the swab are inserted here.
- **Date 1**
  - Enter the date the swabs were taken here.
- **Date 2**
  - If swabs were taken on more than one date for a single swab box, enter the last date they were taken here.
- **CID label**
  - This is the location on the form where the CID label is affixed.
- **Notes**
  - Any problems should be noted here.

## **11.5 Treatment Forms**

### 11.5.1 Mass Treatment Book (Form 6)

This book is printed out from the database at the time of mass treatment, for use by the mass treatment team to determine coverage. Each page is a unique household in the community, organized by village and balozi leader.

- **CTA1/CTA2**
  - Enter the name of each of the CTAs (Community Treatment Assistants) who are treating this household.
- **Date of visit**
  - Enter the date that the treatment was administered to the family the first time. The CDD may have to return to the family multiple times to complete treatment, but the date that the first family member was treated can be entered here.
- **Section I**
  - This section lists the current residents. Data fields, including name, sex, age, and mother of the child, will be populated prior to printing the Mass Treatment Log Book. These data were collected at the previous census.
  - Absent/Present
    - If a person is absent, indicate with a tic in the appropriate box the reason for the absence: deceased, moved away permanently from the community, or temporarily away from the community.
    - For each person who is present, indicate with a tic in the appropriate box if they were treated or not.
  - Treatment
    - If the person received azithromycin treatment, indicate the dose that was provided in terms of number of pills or ml of liquid.
- In the communities randomized to the intervention, a flag is used to cover this section for any newcomers that have entered the community and been treated within the last month. If newcomers entered the community before one month prior to the MDA, they are still eligible for MDA. Since treatment is administered to newcomer upon their arrival into these communities, this flagging of the Mass Treatment Book ensures that very recent newcomers do not receive double treatment within one month. The CDDs receive training on screening for trichiasis among community residents ages 15 and older. On the MDA form, there is a column labeled for persons age 15 and older: TT screening. For each person of that age group, there are three choices:
  - TT screen negative
  - TT screen positive, and refused surgery
  - TT screen positive and referral
- **Section II**
  - This section permits adding new residents and indicating if they are temporary residents of the household for the purposes of mass treatment. Sometimes the village is inflated with persons who want treatment, and indicate they are part of a household in case that is a requirement, which in this study it is not for purposes of mass treatment. By adding them and indicating they may be just temporary, it helps account for the doses used but does not artificially inflate the census or coverage figures.
  - Name
    - Indicate each new person’s first and last name (and alias if applicable).
  - Sex
    - Note the sex of the person as “M” for male and “F” for female.
  - Age
    - Use this column if no birthdates, or at least year of birth, can be obtained. Use an events calendar specific for each region to estimate the age of the person. If necessary, have other family members assist in determining the age.
    - Avoid having a consensus using round numbers or last digits with 5’s as it indicates digit preference; try to get the person and/or family members to be as precise as possible.
    - For children age less than ten, indicate years and months if possible. For children less than age one year, insert a zero for years.
  - Mother:
    - For each child age less than ten years, insert the two digit person code for the primary caretaker of the child. This will help identify the child in subsequent surveys.
  - Absent/Present
    - Since this section of the form is used to record new arrivals into the household since the last census update, all persons recorded in this section will be marked ‘present’.
  - Treatment
    - If the person received azithromycin treatment, indicate the dose that was provided in terms of number of pills or ml of liquid.
  - The CDDs receive training on screening for trichiasis among community residents ages 15 and older. On the MDA form, there is a column labeled for persons age 15 and older: TT screening. This should be done even if the person is a temporary visitor. For each person of that age group, there are three choices:
    - TT screen negative
    - TT screen positive, and refused surgery
    - TT screen positive and referral

This book is entered into the database as a record of coverage for the community, and if new persons have come into the house as permanent residents, then the census is also updated.

### 11.5.2 Mass Treatment Verification Form (Form 7)

A random selection of households treated on the first two days is chosen to have their mass treatment information verified. The Mass Treatment Verification Form (Form 7) is printed out, one page per household, with the household identifying information pre-printed. Any sentinel children in the household to be verified have their names italicized so that the verification team can make an extra effort to verify that child’s treatment information.

- **Verification Team Members**
  - The names of the verification team members should be written here.
- **Team ID**
  - The ID given to the verification team should be given here.
- **Team Leader Initials**
  - The initials of the team leader should be given here.
- **Date:**
  - The date of the verification visit should be given here. Use DD/MM/YYYY format.
- **Treated with Azithromycin?**
  - Put a tic in the checkbox for each person on the list, either yes, no, or DK for don’t know

### 11.5.3 Community Monitor Household Checklist (Form 8)

Each CM will check the households within their balozi weekly to determine if newcomers or travelers have arrived and will determine if any families have left the community. This form will be used to track this surveillance and KTP supervisors will check these sheets to ensure the surveillance process is correct.

- **HID**
  - The household IDs that the CM is responsible for are pre-printed here.
- **Head of Household**
  - The name of the head of household is pre-printed here.
- **Week number**
  - If someone is present in the household at the time of the check, then the CM ticks ‘present’ under the associated week number.
  - If no one is present within the household at the time of the check, then the CM ticks ‘absent’ under the associated week number.
  - If a household is marked ‘absent’ for 8 weeks or more in a row, and then returns, this classifies that household as a ‘traveler’ and the CM must notify KTP and the CHW of this instance.
  - If a new household or newcomers or travelers arrive, the CM must notify KTP and the CHW of this instance.

### 11.5.4 Newcomers & Travelers Treatment Form (Form 9)

Identification of newcomers and travelers in the intervention communities is described in Section 7.3 Newcomer and Traveler Census. Our definition of newcomers and travelers are described in Section 1.1 Overview and focuses on newcomers that are at highest risk of infection, those with children under age 10 that have moved in from outside the district.

Whenever a new household moves into an intervention village, that household should be treated if they the household meets certain criteria. The CM will first call KTP to get a new household ID and then will fill out an Add New Household to Census Form (Form 1) to give each resident a unique ID. Both the CM and the CHW that is to treat the household will then visit the household together. The CHW will use the person IDs to fill out the Newcomers & Travelers Treatment Form (Form 9).

- **HID**
  - The 7 digit household ID is written here.
- **Balozi Leader name**
  - The name of the balozi leader is written here.
- **Head of Household**
  - The name of the head of household is written here.
- **CTA1/CTA2**
  - The CTA number(s) of the CHW(s) are given here.
- **Today’s Date**
  - The date of treatment is given here.
- **Next MDA Date**
  - The date of the next scheduled mass treatment for the village is given here. This date is known by the CHWs. If the date of the next MDA is within 1 month of the current treatment, then the household should be informed that they will receive treatment and do not need to attend the MDA.
- **Question 1: Is there a child under 10 years of age in the household?**
  - If the response is ‘no’ then no treatment is required.
- **Question 2: Outside of Kongwa?**
  - This question is used to determine if the household was in an area where they could have been exposed to infection. The wording of the question changes depending if the household is a newcomer or a traveler.
  - Households that have children under 10 and have traveled to, or have moved from, a village outside of Kongwa should be treated.
- **Person Number**
  - The 2 digit person number from the Add New Household to Census Form (Form 1) should be entered here.
- **First Name**
  - The first name of the person being treated should be entered here. Special care should be taken to make sure that the person numbers and names for the Add New Household to Census Form (Form 1) and Newcomers & Travelers Treatment Form (Form 9) match.
- **Treatment**
  - The dosage of the treatment should be given here as either tablets or ml.
  - If treatment is refused, then ‘0’ should be inserted in the tablets and ml columns.

### 11.5.5 KTP Supervisor Form (Form 10)

A KTP team member will travel to each village at least once a month to check on the accuracy of the CMs and CHWs in determining households that need to be treated between MDA in the intervention villages. The questions on this form allow the KTP supervisor to determine whether or not the household should have been treated.

- **HID**
  - The household identification number is written here.
- **Name of the Balozi Leader**
  - The name of the balozi leader is written here.
- **Head of Household**
  - The name of the head of household is written here.
- **KTP Supervisor**
  - The initials of the KTP supervisor are written here.
- **Date**
  - The date of the KTP supervisor visit is written here. Use DD/MM/YYYY format.
- **Newcomer Household**
  - This section of the form is completed if the household is a newcomer household.
  - (A1) Is there a child under 10 years of age in the household?
  - (A2) Where did this household live before moving here?
  - (A3) Was this household eligible for treatment?
    - If A1 is ‘yes’, and A2 is ‘Outside Kongwa’, then the household was eligible to be treated.
  - (A4) Did this household receive treatment?
    - This question should be asked whether or not the household should have been treated to see if the CHWs are treating households that they should not be.
- **Traveler Household**
  - This section of the form is completed if the household is a traveler household.
  - (B1) Is there a child under 10 years of age in the household?
  - (B2) Where is the farthest this household traveled to?
  - (B3) Was the household absent for 8 weeks or more?
  - (B4) Was this household eligible for treatment?
    - If B1 is ‘yes’, and B2 is ‘Outside Kongwa’, and B3 is ‘yes’, then the household was eligible to be treated.
  - (B5) Did this household receive treatment?
    - This question should be asked whether or not the household should have been treated to see if the CHWs are treating households that they should not be.
- **New Guardian/child in an existing Household**
  - This section of the form is completed if the household has newcomers that consist of at least one child under age ten years and a guardian.
  - (C1) Has a child under 10 years of age with their guardian moved into an existing household?
  - (c2) Where did these new people live before moving here?
  - (A3) Was this household eligible for treatment?
    - If A1 is ‘yes’, and A2 is ‘Outside Kongwa’, then the household was eligible to be treated.
  - (A4) Did this household receive treatment?

This question should be asked whether or not the household should have been treated to see if the CHWs are treating households that they should not be

Chapter

12

# **Chapter 12: Training and Certification of Staff and Clinics**

## 12.1 Introduction

The purpose of certification and training investigators and study staff for the ASANTE trial is to ensure that all study procedures are performed in a consistent and replicable manner in accordance with the study protocol. Additionally, certification and training is intended to ensure that all parties are in compliance with local and federal regulations regarding human research.

## 12.2 IRB Compliance

## 12.3 Field Training and Certification

We will hold a five-day workshop in late January 2013, prior to the start of the trial where the team from Tanzania will get together for extensive training, standardization, and certification in study protocols and procedures. This workshop is part of the quality assurance mechanisms for this trial, and assures that all members are performing in an identical fashion, and to protocol (described above for each facet of the trial). Two more training workshops will take place in the first year for the intervention supervisors and CM/CHW teams, as their communities get close to enrollment date. We describe the January workshop below:

## 12.3.1 Trachoma Grading

The standard for this trial will be the WHO simplified grading scheme, consisting of follicular trachoma (TF), trachoma intense (TI), and trachomatous scarring (TS). Dr. Sheila West will be the senior standard grader against whom all the trachoma grading will be compared for quality assurance purposes. Dr. West and a consultant will conduct a re-training session which includes the WHO training set, supplemented by slides to teach the characterization of trachoma and other signs as seen in the field. After verbal discussion and agreement with the slides, the two graders are taken to the field for field experience grading, and a standardization trial. To be certified for this trial, the graders must have a kappa of >0.6 for the sign of TF.

Field experience consists of graders observing eyelids together and agreeing on the grade, followed by one-on-one with the consultant and Dr. West, discussing eye lids clinical signs. When Dr. West feels the trachoma graders are sufficiently restandardized, then we will have a formal trial, where 50 children are all graded by each grader, masked to the grades of the others. The children walk, with a number attached to each shirt, to each grader who will assess the presence and absence of each sign. At the end, the score sheets are entered and kappa coefficient assessed for each grader compared to the consultant/Dr. West.

If the kappa coefficient is 0.6 or greater, then the grader is certified for the trial. If the kappa coefficient is worse, then the grader must do more work with Dr. West to achieve a better agreement. No grader can be part of the data collection for the trial unless certified.

If, during the course of the trial itself, the review of photographs indicates that a grader is drifting, and the kappa coefficient for his photogrades compared to those read by Dr. West is less than 0.6, then the grader receives suspension of certification subject to re-training. The re-training can be done in country by another grader who is still certified. Once that grader and the suspended grader have carried out a trial in masked fashion, and agree with kappa coefficient =0.6, then the suspended grader can be reinstated provisionally. At the next clinical grading session, he must be certified again by Dr. West’s review of the grades of the photographs compared to the clinical grades.

Scarring will be graded on the basis of photographs. Scarring will be assessed using the expanded scarring grading scheme developed at Wilmer by Wolle et al [1]. Two photograph graders will be trained on using the grading scheme with Dr. West, and must achieve a kappa of 0.6 or greater for grading severity of scarring to be certified to grade for this study. Thereafter, each photograph will be read independently by the two graders, with open adjudication of disagreements. Dr. West will cast the deciding vote during adjudication.

### 12.3.2 Photography

As noted above, the study staff will be retrained with a consultant on the proper use of the handheld Nikon D-series with macro lens to achieve gradable images of the upper lid. This training will consist of orientation to the camera, its parts, and care of the camera. Procedures will include shooting the identification number first, labeling the photo log with the patient identification number, and downloading and storing images on a CD for transmittal to Dr. West at JHU.

Participants will practice shooting images of each other’s eye lids until acceptable images are obtained, while shooting within a few seconds. Once the trainers feel the photographers are sufficiently ready, they will take their equipment to the field and practice at the same time as the trachoma standardization.

Certification will be granted once photographers pass an oral test on camera care, trouble shooting, and procedure, and can submit three consecutive acceptable photographs, with ID numbers, from the field, downloaded onto a CD. Photographers who do not pass this certification cannot be used in the trial.

### 12.3.3 Laboratory Specimen Handling

The standard for this trial are the protocols developed and used in the PRET trial, described in detail in Section 8.3.3 Laboratory Specimen Collection. We will invite Dr. Bruce Gaynor of UCSF, who led our previous standardization workshop in Ethiopia in 2007 on photography and laboratory procedures, to host a session reviewing the laboratory protocol and details. This will be followed by a field session where those study staff who will be collecting specimens will be observed for proper technique, including: opening packages, swabbing the eyelid and inserting the swab in the vial, and labeling. Open discussion of technique will take place for the first and possibly second attempts. The lab technician will need to perform properly on two eyelids to be certified for the trial. No lab technician can be part of the data collection for the trial unless certified.

Throughout the trial, air swabs are taken to check for possible field contamination. If any of the swabs are positive, then the lab technician receives provisional certification, subject to review by another, certified, lab technician in country. Once retraining is accomplished, the lab technician is recertified, subject to review again of his field control swabs.

### 12.3.4 Study Forms

The standardization workshop will include a session on the study forms, and the training and certification will be led by Ms. Munoz, lead statistician and data manager for this study. She will conduct a formal session reviewing, in detail, the forms for all participants at the workshop, so all are familiar with each form, each field and how to fill it out properly. The session will include practice session with other team members posing as heads of household for census, and practice with census updates, mass treatment, examination, and intervention forms.

In the field, those who are doing trachoma grading will practice filling in the tablet ocular forms and the mass treatment forms. Those supervising census will practice on households in the village with census forms and updates. Standardization exercises will be led by Dr. West on observations of a clean face, presence of a latrine, and the cook stove questionnaire. These will be pilot tested in the village during the time of trachoma assessment.

Ms. Lee and Dr. West will lead the workshop on training the supervisors of the intervention and the CMs, as described in detail below. Each supervisor and CM must undertake an oral test on filling in the forms properly and how to handle problems and each must demonstrate acceptable performance in filling in each of the forms, as observed by Ms. Lee. Ms. Lee will then certify the team member on the study forms. The certified team members are then required to teach the CMs, using the same methods, and issue certification. Only the initials of certified study team members will be allowed on forms sent for data entry. Only team members who have been trained and certified by the study team member from the workshop can use study forms for this study.

### 12.3.5 Data entry

Data entry managers will be included in the Workshop. They will be part of the session on study forms, to understand details on the content and completion requirements for the forms. They will then attend sessions on data entry programs for the study that will cover the following topics:

- How to maneuver through the database
- How to do data entry for each form
- Double data entry
- How to troubleshoot problem forms
- When to send forms back to the field for resolution
- Meaning of error messages and warnings
- Saving databases
- Copying and encrypting databases for back up
- Printing reports
- Signing off and proper storage of paper forms
- Tips on using MS Access
- How to send data to Johns Hopkins in a timely fashion.

The database will have a manual of operation for use by the data entry staff at KTP, statisticians and data managers.

The data managers must be certified by Mr. Dreger, lead study programmer at JHU, or Ms. Munoz on data entry of all forms. Certification will consist of successful entry of a set of practice forms, saving and copying the database, proper signoff on forms, proper storage of forms and proper transmittal of encrypted data to JHU Only data entry persons who have been trained and certified will be permitted to do data entry for this study, and only their initials will be recognized for data entry. Only the team leader for the data entry team, or Mr. Mkocha, is authorized to send data to JHU.

### 12.3.6 Intervention and Supervision of Intervention

The KTP staff members who will be supervising the CMs and CHWs will be included in the workshop with training by Dr. West, Ms. Lee (the study manager in Baltimore), and Mr. Mkocha (the Project Director at KTP). The supervisors at KTP will also be trained on the responsibilities of supervising their CMs. This workshop will review the following:

- Charting phone calls
- Procedures for confirming newcomer status
- Procedures for confirming travel status
- Overseeing treatment logs
- Corrective actions in the event of errors

Additional topics covered will include: the job responsibilities for the CM, the protocols to be followed, and the forms to be filled in by the CM and the CHW. The job responsibilities for the supervisor, and their forms, will also be reviewed in detail so the entire intervention process and documentation of that process is clear.

Practice sessions with each other, assuming roles as CMs, CHWs, and supervisors will be carried out until each supervisor is able to describe in detail the job and forms needed to document performance. Provisional certification will be issued after a simulated practice session with Mr. Mkocha posing as a CM during a phone call to report a new family and a monthly review. The study manager and Dr. West will observe key points and issue provisional certification.

Once the KTP staff supervisors are trained, there will be a follow-on training for the CMs and CHWs conducted by Ms. Lee, Mr. Mkocha, and the KTP staff supervisors. We plan to train them in 3 sessions of 8 communities each, one group in January, one group in April and one in July, as the communities are enrolled during the year. Detailed instructions on the protocols and forms for the CMs to follow will be conducted by Mr. Mkocha and Ms. Lee. Practice sessions will be held with each of the CMs and CHWs, led by their supervisors. An oral quiz will conclude the session. CHWs will also be trained on how to measure the height of children and appropriately estimate azithromycin dosage. Each CM and CHW must complete the training to be certified for the trial.

Each village team will start using their training immediately, even before the community has their baseline survey and MDA (if indicated), as practice sessions. Ms. Lee and Mr. Mkocha will supervise this practice activity after the training, together with the KTP staff supervisor, by making weekly visits to all the CMs to observe house visits, and can institute corrective action at that point. If the surveillance and the supervised practices proceed as per protocol, then a final certification will be given to both the CM and the KTP staff supervisor. If there are more than two protocol violations observed over the 2-3 week practice run that are attributed to the KTP staff supervisor, then Mr. Mkocha will continue to oversee the supervisor’s activities for the next month. If there are more than two protocol violations observed over the month run attributed to the CM then the KTP staff supervisor will continue the weekly visits until at least 4 weeks go by with no violation of protocol. At that point, final certification is granted to the CM and the supervisor can fall back to monthly visits.

## 12.4 References

1. [Wolle MA](http://www.ncbi.nlm.nih.gov/pubmed?term=Wolle%20MA%5BAuthor%5D&cauthor=true&cauthor_uid=18936137), [Muñoz B](http://www.ncbi.nlm.nih.gov/pubmed?term=Mu%C3%B1oz%20B%5BAuthor%5D&cauthor=true&cauthor_uid=18936137), [Mkocha H](http://www.ncbi.nlm.nih.gov/pubmed?term=Mkocha%20H%5BAuthor%5D&cauthor=true&cauthor_uid=18936137), [West SK](http://www.ncbi.nlm.nih.gov/pubmed?term=West%20SK%5BAuthor%5D&cauthor=true&cauthor_uid=18936137). Age, sex, and cohort effects in a longitudinal study of trachomatous scarring. [Invest Ophthalmol Vis Sci.](http://www.ncbi.nlm.nih.gov/pubmed/18936137) 2009 50(2):592-6. Epub 2008 Oct 20.

Chapter

13

# **Chapter 13: Quality Assurance Policies and Procedures**

## 13.1 Overview

Several policies and procedures are built into this project in order to provide assurances of quality in the data collection, processing, and management, as well as assuring proper treatment of all participants. General QA procedures include:

- Intensive training sessions: All individuals involved in the study will undergo training and re-certification for all study procedures for which they are responsible. Training and certification procedures are described in Chapter 12.
- Development of standardized forms and procedures for completion of forms. This includes procedures for recovery of missing data and changing responses, if necessary, as described below.
- Monthly data editing, report generation, and reconciliation, with feedback to the clinical sites and the laboratories by each Data Coordinating Center. These procedures are described extensively in each Chapter. The process of providing periodic feedback on missing data or incomplete or out of range data is critical to timely recovery, identification of problems, and remedial actions to ensure that problems are solved.
- Monitoring of quality of trachoma grading and laboratory procedures used in the field, and in the laboratory. The Data Coordinating Center will review the adherence to randomization procedures, treatment administered, timely follow-up of villages on schedule, agreement among graders for trachoma assessment in collaboration with Dr. West, and laboratory evidence of contamination of specimen collection or inadequate specimens. If the review uncovers a problem, the Data Coordinating Center will notify the Study Site Principal Investigator, as well as issue reports to the Executive Committee. The clinical sites will immediately institute retraining procedures, including suspending a staff member from performance of the task until adequate performance can be demonstrated.
- The project director has planned bi-weekly meetings of the census, survey, treatment, surveillance supervisors, and the Data Entry Teams, to review reports and study progress, and address problems identified by the Data Coordinating Center.
- Masking of survey team as much as possible, and certainly the laboratory personnel, to treatment randomization. This procedure avoids bias in detection of infection, and determination of outcome.
- Active oversight by the Project Director in the field at the commencement of each study visit. The project director will ensure the next phase of each part of the project proceeds smoothly, the data flow is steady, and the feedback for the new phase is in place.
- Site Monitoring with 6 monthly site visits by the Study Site Principal Investigator. These will focus on adherence to protocols, review of team meetings, and resolution of any issues.
- Creation of an electronic trail to any data modifications and limiting access to the main database. Extensive back up procedures that protect the quality of the database. These procedures help ensure the integrity of the database.
- The Executive Committee will meet every six months to monitor study progress, address any issues arising from data quality monitoring, review safety reports and review consent procedures.

## 13.2 General Guidelines for Form Completion

A significant component of quality assurance for data collection is the proper completion of forms. Detailed instructions for the completion of each form are provided in Chapter 11: Study Instruments, so this section is intended to provide guidelines for the completion of all forms. The following rules apply when completing the forms:

Complete forms in “real time.” Do not fill in the forms at a later time, or more convenient time. Later completion has a high potential for recall bias, missing data, or wrong data. The form must be filled in while the child/family member is present. The most difficult form to accomplish will be the newcomers census form, which will be completed by CMs. KTP supervisors will review these forms and provide feedback to CMs on their form completion performance. If data cannot be obtained, it is better to code the data as missing (see below).

• Use only black ink for initial forms completion

• Use only red ink for alterations/edits to original entries on forms, which must be initialed by the person making the change.

• Check to make sure that ID labels/data and study identifiers are on every page of every form. Forms that do not have this information are to be referred back to the study site project directors for clarification and review.

• Print all written responses.

• Do not change units or re-phrase questions.

• Enter data in the units and number of digits prescribed on form. Do not record fractions. Use a decimal only if it is written on the form. Be sure to include a value behind a decimal point, if applicable.

• Right justify all numbers, entering leading and following zeroes where applicable.

- Left justify all letter codes, leave remaining spaces blank.
- Write pertinent comments in the margins or on the last page of the form.
- Review all responses for completeness; skip patterns and accuracy before signing off on a form.

## 13.3 Missing Data on Study Forms

Missing forms or specimens are discussed in Section 14.3 Data Entry. In summary, procedures conclude with the person responsible signing off on missing items or forms or vials that all attempts to locate have been undertaken and the item is missing. This form is transmitted to the Data Coordinating Center in order to update the database.

If some data within forms are missing and cannot be obtained when the form is reviewed, then an appropriate code is inserted in the empty data field to indicate missing or not applicable.

## 13.4 Changing Responses on Study Forms

In general, responses should not be changed unless there was a transcription error or the discovery of incorrect data by one staff on a form prepared by another. In these cases, the following guidelines should be adhered to:

Preserve the audit trail, so changes can be traced from original response to the changed response. Often, this means nothing more than making corrections on the original form. In the case of responding to questions raised by the data entry clerks, be certain that the missing data/specimen forms are properly filled out and accounting made to site project directors.

Do not obliterate, erase or white-out incorrect response. To correct a response: draw 1 or 2 lines through incorrect response, write correct response next to or about it in red ink, put initials and date in the margin by the correction.

## 13.5 Oversight

The Project Directors will be responsible for overseeing the initiation of all phases of data collection. Thus, together with the principal investigator, the project director will be present at the start of each new phase of data collection, specifically the census, surveys, mass treatment, and new rounds of each. The Project Director will be responsible for ensuring that each type of visit runs smoothly, and will remain onsite until all individuals involved in data collection for that visit type are comfortable with the data collection procedures. They will also be certain that the Data Entry team follows the procedures for each type of data entry, is operating smoothly, and responsibly storing the forms.

Bi-weekly staff meetings of project staff during census, survey, and surveillance supervisors will be held to review progress in enrollment/follow-up, review outstanding problems with forms or data entry, and reconcile reports from the coordinating center. The staff can report field problems, issues in dealing with the villages, and other concerns that might arise. All staff can attend, including drivers.

## 13.6 Adherence to Procedures

The Data Coordinating Center will monitor adherence to the procedures for randomization, mass treatment administered, census and follow-up surveys, identification of new households and travelers, adherence to standards for trachoma grading and laboratory evidence of contamination of specimen collection or inadequate specimens. Mechanisms are in place to ensure that villages are randomized to each intervention arm in the absence of bias by study staff; and the intervention assignment is not known to the laboratory personnel.

## 13.7 Quality Assurance and Training for Field Trachoma Grading

Field experience consists of graders observing eyelids together and agreeing on the grade, followed by one on one with Dr. West, discussing eyelids clinical signs. When Dr. West feels the trachoma graders are sufficiently standardized, then we will have a formal trial, where 50 children are all graded by each grader, masked to the grades of the others. The children walk, with a number attached to each shirt, to each grader who will assess the presence and absence of each sign. At the end, the score sheets are entered and kappa coefficient assessed for each grader compared to Dr. West.

If the kappa coefficient for clinical signs of active trachoma is 0.6 or greater, then the grader is certified for the trial. If the kappa coefficient is worse, then the grader must do more work with Dr. West to achieve a better agreement. No grader can be part of the data collection for the trial unless certified.

During the course of the trial, photographic images taken in the field will be organized into folders for each examiner by visit. We plan to grade these sets of images at each survey round, to ensure absence of drift over time. The images are graded by the field trachoma grader, who is masked as to intervention status of the community. The images are also graded by the senior grader, SW, who is also masked to the community intervention status. In this way, any grading trends can be monitored.

The photographic images will be sent to Dr. West for review and grading, as she is the gold standard to which all the field graders have been trained. She will grade each set of 50 digital images for quality of the image, using 2.5 magnification on a Dell screen, and using this system we show kappa coefficient greater than 0.8 for TF and TI between a grader in the field and the same grader grading the photograph digital images on the terminal. We have already published on the high reliability between grading in the field and grading the same person on film based images, with a single grader. [1] This extends our findings to high reliability of grading trachoma in the field and using digital images.

The first observation is for grade-ability of the image. If more than 20% of the images are ungradeable for any one photographer, the clinical site will be notified, certification of photographer will be suspended and the photographer will have to be re-certified.

Dr. West will grade each gradable photograph with a clinical grade for trachoma, using the WHO simplified grading scheme. The grades will be transmitted to the data coordinating center, which will compare the grades with the grades assigned to the eyes in the field.

The quality assurance of grading in the field is a required report that will be presented at six months to the executive committee for review and reported to the DSMC during their annual review.

## 13.8 Quality Assurance for Specimen Handling

The standard for this trial are the protocols developed and used in our previous PRET trial. They are described in detail in Section 8.3.3 Laboratory Specimen Collection. We will invite Dr. Bruce Gaynor of UCSF, who lead our previous standardization workshop in Ethiopia on photography and laboratory procedures. We will host a session where the protocol is reviewed. This session will be followed by a field session where those who will be taking specimens will be observed for proper technique, from opening the packages, swabbing the eyelid and inserting the swab in the vial, and labeling. Open discussion of technique will take place for the first and possibly second attempts. The lab technician will need to perform properly on two eyelids to be certified for the trial. No lab technician can be part of the data collection for the trial unless certified.

Throughout the trial, air swabs are taken to check for possible field contamination. If any of the swabs are positive, then the lab technician receives provisionally certification, subject to review by another, certified, lab technician in country. Once re-training is accomplished, the lab technician is re-certified, subject to review again of his field control swabs. Data on field contamination results will be presented to the DSMC.

## 13.9 References

1. West SK, Taylor HR. Reliability of photographs for grading trachoma in field studies. Br J Ophthalmol 1990;74:12-3.

Chapter

14

# **Chapter 14: Data Management and Routine Reports**

## 14.1 Overview

Well-designed data management infrastructure is an essential part of any field study, given the large number of people and places involved in the data collection process. In Baltimore, Ms. Beatriz Munoz has overall responsibility for overseeing her team of data managers. On site in Kongwa, Mr. Mkocha will be responsible for the supervision of data collection, processing, and/or storage on site, and Ms. Hadija Hugo, the data entry team leader, will have day-to-day responsibility for the data management and transmission of data in a timely fashion to the Data Coordinating Center in Baltimore. Once the census, survey, MDA and intervention supervision teams have finished the field work in a community, she will oversee data entry and management at the central office. Mr. Mkocha will have ultimate responsibility for regularly checking with each study team member to evaluate data flow. Mr. Mkocha’s performance will in turn be monitored by Ms. Lee in Baltimore, who will review the regularity of specimen shipment and data flow to Baltimore, and carry out quality checks on the data as described below. Monthly feedback on performance will be discussed with Mr. Mkocha via teleconference on regularly scheduled calls.

All study forms were created in English and they will be translated to Swahili. The process of translating forms is a multi-stage process that requires a minimum of two individuals fluent in both English and the local language. One individual will translate all forms into the local language. Once the translation is complete, the forms will then be given to a second translator who will translate the forms back into English. Both translators will review the forms for cultural sensitivity and understanding, and will report any problems to the principal investigator. The newly translated English-language forms will then be compared to the original forms to determine any differences that arose during the translation process. If corrections need to be made, the principal investigator will meet with the first translator to discuss how the differences arose and what changes need to be made to correct these differences. The first translator will then make the changes to the local language forms and give them to the second translator, who will again translate the forms into English. This process will continue until all participants in the translation process are satisfied that the local language and English language forms are interpreted in the same manner.

Training manuals and final versions of forms will be generated prior to trial commencement. Follow-up visit forms and monthly intervention supervisory forms will be computer generated prior to the visit with the information on the house/subject locator, or balozi leader locator, already included on the forms. These forms will be produced based on information in the master files kept at the Kongwa central office. In addition to the forms, the computer program will print labels specific to each visit with the subject’s identification number and visit number. The labels will be used to link laboratory specimens and photographs to the participants, as well as to keep the inventory of the samples and of the photographs taken.

## 14.2 Daily Data Management Activities in Field

Each day when the different teams return from the field, they will bring two types of folders with them. Each folder type must receive special attention. The first folder will contain the completed visit forms for participants who were seen that day, or completed mass treatment forms for families, and these are reviewed for completeness and legibility and sent for data entry. The second folder will contain the forms for all participants whose visit is still pending, or whose family members have not been accounted for with treatment. This folder should be brought again to the field on the next field day, and receive priority attention for completion following the details described in Chapter 11: Study Instruments.

The Intervention team supervisors will also have forms from the CMs and CDDs, as well as their supervisor forms on their reviews of the CMs and CDDs. At each monthly visit, these are checked for completeness and legibility, and that each newcomer family and traveler family has met criteria for treatment and been treated, according to the guidelines as shown on the forms. If treatment has not occurred, a reason is provided.

Once all forms have been accounted for, each form should be reviewed for completeness following the steps outlined below.

1. Review each form for legibility and completeness of items entered. If any item has not been completed, place the form in the “For joint review” folder. Forms in the joint review folder should be reviewed at the end of the day with the person who completed the form.
2. Once all forms have been reviewed, take the stack of forms with questions to the person(s) who completed the form(s) and determine why the item(s) was/were not completed. If possible, complete the item with the assistance of the person originally completing the form. If the item is blank and the person who completed the form cannot return to collect any missing information to complete the item both the study coordinator and the person who originally completed the form should place their initials next to the blank item and place an “M” in the space where the data should have gone.
3. Return all forms to the appropriate folders for data entry.

## 14.3 Data Entry

A customized data entry system has been developed in Microsoft Access by programmers at the Dana Center at Johns Hopkins to facilitate data entry and management at the KTP offices. Double data entry of key fields is used to ensure quality, with any discrepancies resolved with Mr. Mkocha. (Our edit rates for the Gates trial have been extremely low, in the ocular form for example, we have reported to the DSMC a rate of 434 edits /179,760 fields, or a rate of 0.002). The Project Director will install the system on available computers in the Kongwa office and Ms. Munoz will fully train the data entry clerks, and the project manager on how to use the system. The office uses distributed data entry on five computers which access a master file over an isolated network system. All computers are attached to UPS batteries that permit orderly shutdown in the event of electricity failure and power restarts, and we also have two generators at the office to permit no disruption in work flow. Uses of the data system will include:

1. Electronic inventory of forms
2. Double data entry of key fields
3. Data editing
4. Archived history of editing changes
5. Generation of reports on performance and data quality
6. Production of data collection aids (follow-up schedules, reminders of upcoming and overdue follow-up contacts)
7. System backups
8. Generation of forms

The management of the data will occur at Kongwa and Baltimore. The staff at Kongwa will have responsibility for accounting for the completeness and cleanliness for all data entered and sent to Baltimore, with consistency checks built into the data entry system for study ID numbers, out-of-range entries, missing data, etc. Data entry will be performed using Microsoft Access. The data system is designed such that each type of form is stored as a separate table. Variable names within each table are unique, with the exception of the participant-specific identifier that is used to link tables together. The structure of the program allows the information that is constant across forms (i.e. basic demographics) to be retrieved from the master table to each form within that participant’s visit.

The program contains a series of data checks within each form. In particular, the system will check to make sure that entries are within the allowed range, specific to each item entered. The data entry system also contains a series of checks for internal consistency (i.e. skip patterns) within each table, and does not allow duplicate entries. Finally, reports of missing forms for a particular visit are issued at the end of each data entry day. Key fields in all forms will be entered twice, at the end of a data entry for the form, and the double entries are compared. If all fields are identical, the entry is recorded into the database. Otherwise, discrepancies have to be resolved before the record is saved. Detailed guidelines for quality assurance for data entry are described in Sections 13.2 General Guidelines for Form Completion and 14.3 Data Entry and include date, time, and initials of the data entry person who entered the data, and proper procedures for storage of completed forms and forms that need resolution. The Dana Center Data Coordinating Center under the direction of Ms. Munoz will have responsibility for periodic reports on forms/data that are missing and not accounted for, cross form checking for consistency, creation of the Master Data File, quality assurance reports for the field, and the performance of staff, particularly performance by the community intervention team.

We also ensure quality data with frequent backups. In Kongwa, at time intervals during the day, and at the end of the data entry session, the computer prompts the user to do a backup save. The database is copied as a text files to another subdirectory in the hard drive, and to an external drive. At the end of the day, the access database will be copied to an external drive. A different folder within the external drive is assigned for each day of the week, so that at all times we will have backup data to the end of the day for the last five days. Each week the data will be transferred to the Baltimore office by Ms. Hugo or Mr. Mkocha.

General guidelines for data entry:

1. Each clerk will be assigned one community at a time for data entry. The data entry clerk should only enter forms from that community on the computer assigned to him/her.
2. Always, enter census (census-update data) forms before any other forms (treatment books, log books, etc.). Upon completion of data entry of a specific village visit, the data entry clerk should file the village-visit folder in the “completed data entry” file cabinet.
3. Each form should be initialed and dated by the data entry clerk at the time of entry. If there are questions regarding a specific form, the form should be placed in a designated folder for consultation with the data manager team leader later that same day.
4. Enter all items on the forms exactly as they appear. Do not attempt to determine what the person completing the form meant; ask the project manager if you have questions.

## 14.4 Preparation of Data for Transfer to JHU Data Coordinating Center

The project director will supervise data entry. Each week, the team leader or Mr. Mkocha will email the data to the Dana Center where the study statistician will perform additional data checks. The actual databases are not sent because of their large size. Instead, the database contains automated procedures to export its data to a time-stamped folder containing one text file per database table.

To assure confidentiality, all data will be sent encrypted and pass-word protected. Before sending the files via email, they must first be encrypted. The AES 256 standard will be used to encrypt the time-stamped folder the data files reside in. A password will be sent to the Kongwa office to use in the encryption process. We have used this system for the past five years in the trial with no breakdown in communications or loss of data.

To zip and encrypt the time-stamped folder:

1. Select the folder that you want to send
2. Choose **Winzip**, and then **Add to Zip file**…
3. Your options may be slightly different, depending on the installation of the WinZip software.
4. Under Options, choose **Encrypt added files**
5. Push the **Add** button
6. Enter the password provided to you by Johns Hopkins
7. Reenter the password
8. Under Encryption Method, choose **256-Bit AES encryption**
9. Press the **OK** button
10. You may now exit the WinZip utility and email the resulting compressed file (ending in .zip)

## 14.5 Data Editing

At the Dana Center the study data manager/statistician will perform additional data checks as all files come in. Discrepancies will be sent to the project manager for reconciliation by reviewing the original data forms. Corrections will be noted on the printout and returned to the Dana Center. Mr. Mkocha will make edits to the master file and is the only person with permission to make edits to the master database in Kongwa. Records being edited, along with the date and time, will be automatically archived into an external database prior to implementing the changes. To create an audit trail, a report containing the operator, the type of transaction, the date, the old values, and new values will be generated. Ms. Munoz or her team will make edits to the master database in Baltimore, and they will be the only persons with permission to do so. As above, an audit trail is created.

### 14.5.1 Specimen Transfer Data Procedures

During field work at collection time, laboratory samples will be placed in collection boxes that have been numbered in advance. The box number as well as the label identifying the specimen will be recorded in both the ocular specimen shipping list and the corresponding ocular exam form. At the end of the field day the specimen boxes will be storage in a freezer that will be kept at -80°C. Once a visit has been completed, specimens for that visit will be packed according to the procedures described in Section 8.3.3.4 Shipping and Storage of Samples to JHU Laboratory and sent to the Baltimore laboratory for processing. In accordance with the GenProbe ASSAY™ protocol, swab samples taken in the field will be transported on ice in a closed, insulated container until arrival in Baltimore, where they will be stored at -80°C for later analysis. Samples will be imported in the USA as per the CDC Permit to Import/Transfer Etiological Agents or Vectors of Human Disease.

At the same time, a copy of the shipping log for each box will be sent. The laboratory will be responsible for logging in samples and report to the Dana Center any discrepancies between the shipping log and the actual shipment. The Dana Center data management team will work with the project manager in Kongwa to resolve all inconsistencies.

## 14.6 Generation of Reports

Monitoring reports are key to providing feedback on data quality and are an integral part of the quality control system. Reports will be issued for each study visit with summary information on key variables. In order to explore problems with standardization, the reports will be stratified by community, and by interviewer/examiner. If any irregularities are detected, the principal investigator will be informed and restandardization of pertinent procedures will take place.

Participant tracking reports will be generated for each visit. If there is no accounting for a sentinel child at a visit, a report will be given to the project director in Kongwa who will determine what happened and if there is a note entered as to the reason for no follow up. If the visit was carried out, but no data have yet been entered into the data system, he will make sure that the data for that visit are entered.

Intervention tracking reports will also be generated for each intervention community at the end of each monthly supervisory visit. All CMs must be accounted for, the report of their household visit accounted for, and all newcomers and travelers identified must have treatment accounted for. Lack of accounting will generate an error report that must be rectified immediately.

At each visit and for each community the data entry team leader in Kongwa will be responsible for supplying the field team with a census book to update the census with new household members, permanent moves out of the village and deaths that occurred in the previous six months. The census book will be generated by clicking the appropriate bottom under the reports menu of the access database.

Once the data entry for the census update has been completed, the computer will select at random the sentinel children to be examined, and the ocular forms and corresponding labels for the selected children will be printed. If the survey visit will be followed by annual mass treatment, treatment forms for each household will be also generated.

Reports will be issued for each survey visit with summary information on key variables: trachoma status by age, by examiner, and the correlation with infection. In order to explore problems with standardization, the reports will be stratified by community, and by interviewer/examiner. If any irregularities are detected, the study chair will be informed and restandardization of pertinent procedures will take place.

Village tracking reports will be generated for each visit to ensure surveys are done within the window. These reports will be given to the KTP project manager, who will verify that either the surveys were conducted or if not, schedule a follow-up visit. If the visit was conducted, but no data have yet been entered into the data system and it is past due date, he will make sure that the data for that visit are entered.

Periodic reports for quality assurance will also be prepared. These include the following:

- Trachoma grading: The Coordinating Center will receive the grades from Dr. West on the ocular photographs for each grader at each visit. These will be compared to the field grades given to the same photos. Agreement will be determined, using the kappa coefficient, for TF. A report is generated on the agreement to the Study site investigator and to the Executive Committee. If agreement is below kappa 0.6, then the Study Site investigator will responsible for carrying out re-standardization exercises as described in Chapter 12: Training and Certification of Staff and Clinics.
- Field Laboratory specimen reports: the Coordinating Center is responsible for receiving the data on the positivity of the air field specimens, and generating a report on the rate of positivity by visit and by lab technician. A report is generated to the Study Site Investigator and to the Executive Committee. If field contamination is greater than 1%, then remedial actions in the field are required to bring the field Procedures back to standard, as discussed in Section 13.8 Quality Assurance for Specimen.

- Intervention reports: the Coordinating Center is responsible for generating monthly reports on the status of the intervention, noting if there are more than 1% of new households that were missed by the CM and detected by the KTP supervisor, and the proportion that fell outside the two week window for treatment. The report will also describe the status of number of households where travelers were identified, and households where families are still away. Ms. Munoz will be in charge of discussing these and the need for remedial action with the KTP Project Director, Mr. Mkocha, because Dr. West is masked to the community treatment assignments.
- Reports on photograph grading of scarring. The Coordinating Center is responsible for generating monthly reports on the agreement of scarring grading and adjudication. If the initial kappa grades fall below 0.6 grading severity of scarring, then Dr. West will review the grades and determine, based on the adjudication, if one grader needs to have provisional certification and re-training.

## 14.7 Data Storage

At the office in Kongwa, data will be stored in locked file cabinets. Electronic databases in Kongwa will be backed up to an external drive on a daily basis and periodical backups will be stored off-site. At the Dana Center, for the analysis phase, the data will be transferred into a Sun station UNIX base system, where SAS permanent data files will be created. Three generations of backup files are kept at all times for this computer. One copy is housed off-site. In the central computer, tape backups are performed daily. Four tapes are used during the week (labeled Monday to Thursday). There are four Friday tapes (Friday1 to Friday 4) for each Friday of the month. At the end of every week (Friday), backups are done on the weekly tapes. There are twelve monthly tapes (January to December). At the end of each month, the backup is done on the monthly tape. This procedure ensures the possibility of retrieval of old versions of files up to one year old.

To maintain data and study documentation, codebooks with question-by-question specification of all questionnaire responses will be created and updated periodically. Furthermore, a complete description of all data files will be kept. This description will include the file creation date, the number of records, the record layout, the field names and description and a list of allowable codes for each field.

Chapter

15

# **Chapter 15: Adverse Events**

Azithromycin is being given to these communities under the auspices of the National Trachoma Control program in Tanzania. They have determined, for programmatic reasons, that widespread coverage with azithromycin is an acceptable community risk for the benefit of eliminating blinding trachoma. Approximately 100 million doses of oral azithromycin have now been distributed for trachoma, and reports of serious side effects are essentially non-existent. This may be due in part to minimal surveillance. In fact, where carefully monitored, there were actually fewer GI side effects after taking azithromycin compared to topical tetracycline, perhaps because azithromycin treats some cases of diarrhea. Azithromycin is generally well-tolerated. The most common side effects of azithromycin and erythromycin are diarrhea or loose stools, nausea, abdominal pain, and vomiting, each of which may occur in fewer than one in twenty persons who receive azithromycin. Rarer side effects include abnormal liver function tests, allergic reactions, and nervousness.

All community residents will be advised to alert the village health worker if they experience, within two weeks of mass treatment, a serious adverse event, defined as diarrhea, nausea and vomiting for more than two days, hospitalization for any cause, or death in a family member. All residents will be urged to report any adverse events to the local CHW, who will keep a log of these events as required by the donor. These events will be reviewed by KTP at the next six month survey, and entered into the adverse event database for reporting to the Data and Safety Monitoring Committee. If, for any reason, the village resident needs further care, they will be referred to the nearest health center for examination and treatment, and the most appropriate action will be taken to provide immediate care, in accordance with the policies in Tanzania.

Chapter

16

# **Chapter 16: Data Analyses Plan**

16.1. General Approach

All analyses will be conducted based on the intention-to-treat principle; therefore, communities will be analyzed according to their randomization assignment regardless of whether the newcomers/travelers had received treatment. Although the two arms will likely be balanced on baseline variables as a result of randomization, we will compare demographic characteristics and other factors that may be related to the prevalence of *C. trachomatis* infection/follicular trachoma prior to conducting further analyses. Any variables unevenly distributed between the two treatment groups will be included in all multivariate models to control for potential residual confounding.

For each community, the primary outcome will be an indicator variable of whether or not the prevalence of *C. trachomatis* infection in children 0 to 9 years of age at 24 months post-randomization is ≤1%. As a secondary outcome, an indicator variable for prevalence of follicular trachoma, using the WHO grading scheme, a threshold of <5% will be examined.

16.2 Primary/Secondary Outcomes

The primary outcome for the trial is the proportion of communities with infection prevalence at 1% or below in children ages 1 to 9 years at the 24-month survey, comparing the intervention arm to the usual practice arm. Of note, there are no criteria or agreed upon age group for stopping MDA based on infection.

The first secondary outcome is the proportion of communities with trachoma prevalence below 5% in children ages 1 to 9 years at the 24-month survey, comparing the intervention arm to the usual practice arm. We chose below 5% to conform to the WHO criteria for stopping antibiotic treatment based on clinical criteria. Another secondary outcome is to model the change in infection and trachoma in the communities in the usual practice arm with continued rounds of (and stopping) MDA over the two-year trial, including the data from the previous trial. Another secondary outcome is to model the two-year risk of re-emergent infection and trachoma in the communities which have been stopped at the outset and over the course of the trial.

The infection outcome will be based on laboratory evidence of Chlamydia taken from eye swabs of sentinel children at each survey round. The outcome is masked to intervention status as the laboratory performs the analyses of infection masked. The trachoma outcome will be based on clinical grading in the field. Every attempt will be made to have the survey team masked to the intervention status of the community, and careful quality assurances are in place to monitor drift in reading over time.

16.2.1 Primary Aim/Secondary Aim 1

We have population characteristics measured at the child and household level. These will be summarized at cluster (community)-level and then summarized by treatment assignment. The mean of cluster means (SD) for each arm will be presented, along with the median (range) of cluster medians for each characteristic. The Wilcoxon rank sum test will be used to compare the two groups.

*C. trachomatis* infection/follicular trachoma prevalence will be presented for each community and each time period stratified by randomization arm, and the proportion of communities at or below the threshold for infection/ follicular trachoma will be estimated. Differences between arms in the proportion at or below the threshold at 24 months will be tested using a Fisher’s exact test as specified in sample size determination.

Multivariable models will be constructed, and the primary analysis will model the probability of a community being at or below threshold for infection/follicular trachoma at the 24-month survey. Logistic regression models adjusting for potential confounders will be examined. The structure of the models will be as follows:

$$Log\left(\frac{\Pr(Y_{i}=1)}{1-\Pr(Y_{i}=1)}\right)=\beta_{0}+\beta_{1}x_{i}+\gamma z_{i}$$

Where *Yi* is the indicator of whether the prevalence of infection/ follicular trachoma is at or below the pre-specified threshold level for community *i*; *x_i_* is the indicator for randomization arm for community *i,* and *z_i_* is a vector of covariates to adjust for initial infection/trachoma status at community level, average coverage with MDA, and other potential confounding factors including those that, despite randomization, were dissimilar at baseline. A key variable that must be included in the models is a measure of treatment coverage for community i, so that the intervention effect for communities assigned to the treatment of newcomers is adjusted by the level of treatment coverage.

16.2.2 Secondary Aim 2

The data to characterize the expected decline in the prevalence of infection/follicular trachoma over time with annual high-coverage MDA are comprised of biannual surveys for up to 15 time points, for the control communities, and biannual surveys for up to 10 time points for the intervention communities (who would be censored at baseline in this trial, as they have an additional intervention beyond annual MDA). We will model the prevalence *p_it_* for the *i^th^* at time *t* as a mixed effects linear model of the form:

$$p_{it}=(\alpha_{0}+a_{i})+(\beta+b_{i})t+e_{it}$$

Where (*a_i_, b_i_*) follow a bivariate normal distribution with zero means representing the random effects and *e_it_* are the departures of the observed prevalences from the line of the *i^th^* community with intercept (*α+a_i_*) and slope (*β+b_i_*). It is expected that β will be negative, as repeated mass treatment is very likely to induce an overall decline. The primary interest is to determine the *t* such that *α+βt=0*; that is *t=(α)/(-β)* . From the covariance matrix G, and the estimates of α and β we will use the delta method to find the standard error of the log(t)= log(α)-log(-β). Specifically, the variance of the logarithm of t will be given by:

$$Var\left( \log\left( t \right) \right)=\frac{Var\left( \hat{\alpha} \right)}{\hat{\alpha}^{2}}+\frac{Var(\hat{\beta})}{\hat{\beta}^{2}}-2\frac{Cov(\hat{\alpha},\hat{\beta})}{\hat{\alpha}\hat{\beta}}$$

A 95% confidence interval for t will be computed as:

$t exp(\pm1.96\sqrt{Var(log(t)}$)

In the event that a high proportion of the communities have zero or very low a levels of infection, we will consider the use of logit and/or squared root transformations of the prevalence as the outcome. These transformations may be more compatible with the underlying normality assumptions for the model described above.

16.2.3 Secondary Aim 3

Communities with prevalence of infection estimated at or below 1% at prior to baseline, at 6 months or at 18 months will be eligible to have MDA at baseline, one year, and two years stopped as they are considered infection free. We also received an addendum from the DSMC to allow us to stop MDA at one and two years in communities if the prevalence of TF was <5% at 6 months or 18 months. These communities will be monitored for re-emergent infection for at least an additional 12 months. If during the follow-up period the infection level increases to 6% or greater, the community will be classified as having re-emergent infection. The reason for choosing 6% is, with 100 children sampled, the upper 95% confidence for 1% is less than 6%, so we can be sure that this truly represents re-emergence and not random error. The length of the follow-up period for the infection-free communities will be variable ranging from 12 months (for those communities reaching the “infection≤1%/TF<5%” status at the 18-month visit with follow up to the final survey at 30 months) to 2.5 years (for those communities classified as infection<1% at baseline).

FACTORS ASSOCIATED WITH RE-EMERGENT INFECTION

We will examine the effect of treating newcomers and travelers on the risk of re-emergence. The primary outcome will be time to re-emergence measured from the time at which the community is first at prevalence of infection 1%/TF<5% to the time at which the infection rises to 6% (regardless of TF prevalence). Since the outcome is only measured at 6-month intervals, we will use methods for discrete time to event. Specifically, for each six-month interval we will construct a 2 by 2 table with the two rows representing intervention assignment and the two columns representing event status. If the prevalence of infection in a community reaches 6%, that community has reached the endpoint and will not be considered at future time intervals. The tables will be combined using the standard Mantel-Haenszel approach to produce an overall hazard ratio. A similar strategy will be used to examine the effect of community size, percentage of households with latrines, percentage of children with clean faces, and percentage of households more than one hour away from a water source. We are aware of the limitation of few data points and uncertain numbers of re-emergent communities in trying to pre-specify any modeling approach to re-emergent infection and chose to be very basic in the analysis of this secondary aim.

### 16.2.4 Secondary Aim 4

For this aim, the presence of active trachoma signs (TF or TI) is the outcome. We will explore the relationship of active trachoma with putative personal and environmental risk factors such as age of the child, quality of the house, distance to the water source, presence of a latrine, family ownership of bicycles and cell phones. But in particular we are interested in the association between the presence of a cooking fire in the room where the child sleeps and active trachoma while adjusting for the above mentioned factors. First, we will conduct simple unadjusted analysis. Using contingency tables we will compare the prevalence of trachoma in children living in houses where the cooking fire is outside to prevalence of trachoma in children living in houses where the cooking fire is in a room inside the house. For the adjusted analyses we will construct multivariate logistic models that in addition to location of the cooking fire will include the above mentioned factors. Random effects models will be used to account for the within community correlation of trachoma.

In addition to the simple dichotomous outcome, we plan to create a more detailed exposure index to cooking fire. In this case the lowest exposure will be in children who sleep in a room where the cooking fire is outside, and the highest exposure will be in children who sleep in a room with the cooking fire, where the room has no windows. Intermediate exposures will reflect proximity of the cooking fire and windows and doors in the sleeping area. The association of active trachoma and the exposure index will be examined using an approach similar to the one described above.

### 16.2.5 Secondary Aim 5

For this aim, the presence and severity of trachomatous scarring in women is the outcome. In addition to personal risk factors such as age, and markers of socio-economic status we are interested in the relationship between scarring and lifetime exposure to cooking fires. To this end, we plan to create an index reflecting the cumulative exposure to cooking fires. The computation of the index will include as parameters: the year the participant started to cook, the number of days per week she usually cooks, and the number of meals per day that she prepares at home.

The approach to the analysis will be similar to the one described for specific aim 4. However in this case, the outcome is ordinal (degree of severity of scarring), in consequence we will build ordinal logistic models in the multivariate phase.

Chapter

17

# **Chapter 17: Study Organization**

## 17.1 Study Leadership

This trial is a single-site community randomized trial. The primary groups responsible for the oversight of this trial include the Chairman’s Office (Dr. West), the Coordinating Center (Ms. Munoz), the Trachoma Grading Center (Dr. West), and the Laboratory Center (Dr. Gaydos). These groups are part of Johns Hopkins University and will work collaboratively. The study chair’s office is at the Dana Center in Johns Hopkins University where the principal investigator, Dr. West, has overall responsibility for the design, execution, and quality of the trial. This includes the quality of the intervention; the recruitment of communities; the training of study team members; the oversight and coordination with Kongwa Trachoma Project (the collaborating in-country center); managing the study budget; creating and managing written and oral public communications; monitoring human subjects, adverse events, and resolving human subject issues. The study chair’s office will be responsible for the Manual of Procedures and all changes must be approved by the study principal investigator. Ms. Munoz has overall responsibility for the Data Coordinating Center, ensuring that monthly reports are generated for the Executive Committee and any standardization concerns are brought to the principal investigator. Ms. Munoz and her team are responsible for data management and data analysis of the ASANTE trial, preparing all reports for the DSMC and assisting in the preparation and review of reports and manuscripts. Dr. Gaydos has overall responsibility for ensuring the International Chlamydia laboratory is functioning according to CLIA standards, for processing specimens in an orderly and timely fashion, and adhering to agreed upon reporting procedures for the trial.

## 17.2 Executive Committee

The ASANTE Executive Committee is responsible for the following:

- Overall project review; review of progress at yearly meetings, review of data coordination and management at 6 monthly conference calls
- Receiving recommendations from the DSMC and implementation of recommendations
- Making recommendations for resource allocation. Johns Hopkins University will serve as the primary accounting body.
- Review of quality assurance reports from the Data Coordinating Center, and the reports on laboratory and trachoma quality assurance, and make recommendations to the study site principal investigators.
- Serve as a writing committee to oversee the draft of manuscripts using data from more than one site

### 17.2.1 Executive Committee Members & Meetings

The study will be governed by the Executive Committee that consists of the study chair (the principal investigator) who will chair this committee, Ms. Munoz as director of the Data Coordinating Center, Dr. Gaydos as director of the International Chlamydia Laboratory, the NEI representative, and Dr. Quinn as medical advisor. The Executive Committee will meet monthly by conference call to make decisions on day-to-day operational issues, discuss and recommend changes to study procedures and the MOP when necessary, monitor community and patient recruitment, review quality assurance reports and recommend corrective action when necessary. After the first year, the frequency of Executive Committee meetings could be reduced to twice a year.

The main evaluation component of the ASANTE trail will be the following: (1) The determination of a mass antibiotic treatment strategy to achieve sustained elimination of ocular *C. trachomatis* infection and disease reduction, and (2) the development of guidelines for communities to maintain a “graduated” status in an effort to maximize program resources.

## 17.3 In-Country Center

Kongwa Trachoma Project (KTP), on-site community partner, is responsible for implementing the day to day activities of the trial in the communities of Kongwa district, Tanzania. KTP is a local non-governmental organization well qualified to be our partner in Tanzania. We have been partners since 1986, the first trachoma survey in Tanzania[5], and they were the implementing partners for the randomized clinical trial of face-washing and the effect on trachoma[2], the randomized trial of azithromycin in three countries (Tanzania arm)[6], the randomized study of the effect of fly control on trachoma[1], and the recent large NEI funded cross-sectional study on the effects of multiple years of mass treatment on trachoma and infection in Tanzania.[7] KTP is the current partner for Tanzania of the multi-center Gates funded trial (PRET)[4], and have many years of experience carrying out trials on trachoma and infection with *C. trachomatis*. KTP also has experience with instituting and monitoring a variety of community-based interventions, like the face washing trial, the trial of intensive fly spray (which was implemented at the balozi level), and have carried out the community-based mass drug administration program in Kongwa for the last four years. KTP has staff with considerable experience with management and finance, data entry, and all aspects of survey and intervention work.

For the ASANTE trial, KTP will be responsible for recruitment of communities, carrying out the census, performing all aspects of the survey work, storing and shipping specimens to JHU, training (together with assistance from study manager) of CMs, CDDs, and their supervisors, implementing and monitoring the intervention program with verification, implementing MDA where indicated with treatment verification, data entry and data transmittal, and complying with all NIH requirements for foreign institutional partners. Mr. Mkocha, the project director for KTP, will come to Baltimore once per year to attend a planning session with the Executive Committee and report to the DSMC.

Specific responsibilities include the following:

- Ensure the execution of the study per protocol
- Coordinate with the Study Chairman’s office in writing progress reports and budget accounting
- Maintain all Tanzania ethical clearances for the study and, in collaboration with JHU data coordination team, prepare all forms and documents necessary for fieldwork
- Participate in standardization workshop; ensuring relevant team members are present, trained, and certified. Each site must also train team members for each data collection and mass treatment.
- Arrange logistics and itineraries for traveling team members in country
- Purchase, maintain, and organize transport of all necessary study supplies to and within country
- Conduct data entry of data collected, ensure appropriate back up, and send data to data management team for each clinical site.
- Coordinate and oversee shipping of collected study samples per protocol and IATA regulations
- Prepare reports as required for quality assurance and monitoring purposes.

## 17.4 Study Chairman’s Office

The study chair’s office is at the Dana Center in Johns Hopkins University where the principal investigator, Dr. West, has overall responsibility for the design, execution, and quality of the trial as noted above. Her team has fiscal responsibilities for managing the budget, administrative responsibilities for the trial and ensuring that all ethical clearance is obtained and maintained, and oversight of the collaborating partner, Kongwa Trachoma Project. .

## 17.5 Data Coordinating Center

The Data Coordinating Center is at Johns Hopkins, under the direction of Ms. Beatriz Munoz. Ms. Munoz has been the director of the Dana Center Data Coordinating Center for numerous previous trials and epidemiological studies we have conducted, including the NIAID funded ACT trial[6], the Wellcome Trust funded Tanzania study on fly control and trachoma[1], the NEI funded STAR trial[3], and currently the Gates funded PRET antibiotic trial for trachoma control.[4] She and her team will be responsible for randomly selecting the communities for the treatment arms, transmitting the information to KTP and monitoring adherence. She and her team will be responsible for using the data from the previous community surveys done prior to the trial and determining if infection was 1% or below (indicating that MDA does not need to be implemented for this trial), and communicating the MDA status for each community in the trial to KTP. In general, Ms. Munoz and her team will be monitoring adherence to study protocols, monitoring adverse events and other human subject issues, recommending when reports need to be seen by the DSMC to the Executive Committee, data management, and all data analyses. She is responsible for issuing all summary reports for the Data and Safety Monitoring Committee, and all analyses for reports and publications. Further responsibilities are listed below.

- Creation of ASANTE Study Forms
- Creation and maintenance of data entry programs for all sites
- Creation and maintenance of manuals for data entry programs
- Training program on use of forms and data entry programs at standardization Workshop
- Create randomization scheme for villages, and for children within villages, monitor adherence to randomization
- Determine if villages meet stopping rules and issue stopping rule report after 6- or 18-month surveys.
- Monitor adherence to stopping rules
- Create and maintain database for the study
- Monitor receipt of data from the field office after each census, examination survey, and mass treatment
- Analyze and provide data when requested by co-investigators, and for DSMC meetings
- Appropriately back-up all data
- Develop and run data cleaning checks and quality assurance steps as part of data management; communicate with the field site to resolve data problems
- Monitor receipt of specimens at Chlamydia Laboratory, work with the field office to account for any missing specimens, receive data on specimen results from the laboratory, and integrate into the database
- Create reports on study data as required for progress reports on data quality assurance
- Participate in quality assurance activities for trachoma grading and laboratory specimen processing as required

## 17.6 Trachoma Grading Center

Dr. Sheila West at JHU has primary responsibilities for reviewing the consistency of clinical grading of trachoma over time. The following steps will be undertaken to ensure that the grading of trachoma is standard and does not drift over time:

1. Training and standardization of trachoma graders at the outset, in the standardization workshop. Each trachoma grader will go through a training that consists of slide sessions and field experience, until agreement (measured by a kappa coefficient) for grading TF and TI is at least 0.6, measured against Dr. West. Each trachoma grader must be certified for this trial.
2. At each six month survey round, the graders will submit 50 photographs of children they have graded to Dr. West, who will return her photographic grade to the Data Coordinating Center. The Data Coordinating Center will then compare Dr. West’s grades with the clinical grades, and issue a report to her on the agreement. The following steps will then take place
   1. If the kappa coefficient is 0.6 or greater, then certification is maintained.
   2. If the kappa coefficient is <0.6 for trachoma, then the Data Coordinating Center will issue a report to that site for that grader, the certification is set to provisional, and clinical site project director is responsible for assigning a certified grader to work with the provisional grader to achieve agreement (measured by a kappa coefficient) of at least 0.6 in the field.
      1. At the next survey round, the provisional grader again sends 50 photographs of grading. If the agreement is now 0.6 or greater, certification is restored.
      2. If agreement is <0.6 with Dr. West’s, then the grader is notified by the Data Coordinating Center that grading must stop, and certification is rescinded. The clinical-site project director is responsible for making sure the grader goes through a whole re-training process, submitting a set of 50 photographs until agreement is again acceptable.
3. The Executive committee will review, every six months, the certification status of all graders, as provided by the Data Coordinating Center. Data on continued certification will also be presented to the DSMC as part of quality assurance monitoring.

## 17.7 Laboratory Center

Dr. Charlotte Gaydos and Dr. Thomas Quinn at JHU have primary responsibility for reviewing the consistency of each of the laboratories in this study for determining evidence of infection using the GenProbe APTIMA ACT assay. Dr. Gaydos will have responsibility for maintaining the quality control for the laboratory, and investigating any evidence of laboratory contamination. . The quality control procedures are as follows:

- The standard quality assurance procedures are followed that permit the JHU laboratory to maintain their CLIA certification, and allow the CDC to grant permission to import specimens from overseas.
- Each test run for determining positivity contains a *C. trachomatis* (+) control and a *C. trachomatis* (-) control included in each test run of the GenProbe APTIMA ACT Assay™. To test the effect of sample processing, a known positive sample is processed and tested in each test run. (This control is helpful when testing large numbers of negative samples.). An internal control intended to identify specimens that contain polymerase inhibitor is run routinely on each sample. The internal control helps identify false negative results.

The Executive Committee will review yearly the quality assurance certificates for the JHU laboratory. Data on continued quality assurance will be presented to the DSMC.

There is also the possibility of field contamination, which is checked by using the “blue air” specimens. Each laboratory will process these controls masked as to their status as controls. The Data Coordinating Center is responsible for reporting the positivity of the negative field controls to the study site investigators. If the rate is above 1%, then the investigators must take remedial action to correct field procedures to remove contamination. To do so, the lab technician receives provisional certification, subject to review by another, certified, lab technician. Once re-training is accomplished, the lab technician is re-certified, subject to review again of his field control swabs. Data on filed contamination results will be presented to the DSMC by the Data Coordinating Center.

## 17.8 References

1. West SK, Emerson PM, Mkocha H, et al. Intensive insecticide spraying for fly control after mass antibiotic treatment for trachoma in a hyperendemic setting: a randomised trial. Lancet 2006;368:596-600.
2. West S, Munoz B, Lynch M, et al. Impact of face-washing on trachoma in Kongwa, Tanzania. Lancet 1995;345:155-8.West SK, West ES, Alemayehu W, et al. Single-dose azithromycin prevents trichiasis recurrence following surgery: randomized trial in Ethiopia. ArchOphthalmol 2006;124:309-14.
3. West SK, West ES, Alemayehu W, et al. Single-dose azithromycin prevents trichiasis recurrence following surgery: randomized trial in Ethiopia. ArchOphthalmol 2006;124:309-14.
4. Stare D, HardingEsch, E., Munoz, B., Bailey, R., Mabey, D., Holland, M., Gaydos, C., West, S.K. Design and Baseline Data of a Randomized Trial to Evaluate Coverage and Frequency of Mass Treatment with Azithromycin: Partnership for Rapid Elimination of Trachoma. . Ophthalmic Epidemiol 2011;14:5-11.
5. Taylor HR, West SK, Mmbaga BB, et al. Hygiene factors and increased risk of trachoma in central Tanzania. ArchOphthalmol 1989;107:1821-5.
6. Schachter J, West SK, Mabey D, et al. Azithromycin in control of trachoma. Lancet 1999;354:630-5.
7. West SK, Munoz B, Mkocha H, Gaydos C, Quinn T. Number of Years of Annual Mass Treatment with Azithromycin Needed to Control Trachoma in Hyper-endemic Communities in Tanzania. . J Infect Dis 2009;204:268-73.

Chapter

18

# **Chapter 18: Data and Safety Monitoring Committee**

## 18.1 Charter of Data and Safety Monitoring Committee

This charter is for the Data and Safety Monitoring Committee (DSMC) of the Surveillance and Treatment of Community Newcomers and Travelers for Trachoma Control (ASANTE) trials for trachoma. The following table provides an overview of the trials:

| Feature | Details |
| --- | --- |
| ASANTE | |
| Objective | To determine the impact on trachoma and ocular *C. trachomatis* in communities randomized to a newcomer and traveler surveillance and treatment program, compared to control communities with no program. |
| Randomization unit | Communities in Tanzania will be randomized in a 1:1 design. Sentinel children in each community will represent prevalence of trachoma and infection |
| Interventions | A program of surveillance and azithromycin of families new to the community, and families that travel outside the village for more than eight weeks. |
| Treatment Arms | Control arm: Mass treatment with azithromycin if infection is >1% or TF is 5% or greater. Intervention arm: Mass treatment with azithromycin if infection is >1% or TF is 5% or greater plus a newcomer and traveler surveillance and treatment program. |
| Masking | Laboratory for infection masked to intervention status  Photograph grader masked to intervention status  Villages not masked to intervention |
| Sample Size | 48 villages in Tanzania |
| Follow Up | Every 6 months for two years |
| Outcome | Prevalence of trachoma and ocular C. trachomatis infection at 24 months. |

The Charter will define the Terms of Reference for the DSMC, its membership, its relationship with other trial components, and the purpose and timing of its meetings. The Charter will also provide the procedures for ensuring confidentiality and proper communication, the monitoring guidelines to be implemented by the DSMC, and outline of the content of the Reports that will be provided to the DSMC.

## 18.2 Terms of Reference

1. The Data and Safety Monitoring Committee for the ASANTE trial is charged with oversight of the quality of the data collected, and the safety and efficacy of the approaches proposed in the trial. The committee is guided by the principles set out in the National Eye Institute Guidelines for Data and Safety Monitoring of Clinical Trials.

2. As part of its responsibilities for oversight of safety issues, the Committee shall review reports on adverse events at each meeting. If any member expresses to the chair of the DSMC committee concerns for safety issues in the interim, the chair may request a meeting or telephone conference call of a quorum of the members. If the DSMC believes that there is evidence that warrants a change in the procedures of the clinical trial, the Committee will be responsible for drafting a report of their recommendations to the National Eye Institute and to the IRBs as relevant of Johns Hopkins University. If there is no concern, the Committee will also issue a report stating they have no concerns.

3. The DSMC shall meet together at least once per year, and more frequently by telephone if deemed necessary by the members or chair of the committee. The meeting shall be for the purposes of reviewing the accumulated data on the quality of the information collected, the safety of the trial procedures, and the efficacy of the treatment arms. The committee shall issue a summary report, which will be forwarded to the National Eye Institute and each Institutional Review Board.

4. The locations of the trials are in rural Africa. Social, cultural, or political exigencies may arise that may affect the trial or call into question some of the protocols of the trial. The DSMC has accepted the responsibility to serve as a review body for such matters, if requested by members of the DSMC or members of the study team. The members of the DSMC may request a review by the full committee of any events arising from the study which, in the opinion of the committee member, is a serious issue or one in which a response to a government may be necessary. The Study Chair will arrange a conference call as expeditiously as possible with a quorum of members. For this purpose, a quorum is considered three of the five members, with the member from Africa required and the DSMC chair considered a member. The call must include the Study Principal Investigator. The committee will recommend a course of action to the Study Principal Investigator regarding any such events. If the study leadership does not concur with the DSMC's recommendation, it will be the responsibility of the study leadership and DSMC Chair to reach a mutually acceptable decision. A minority report will be issued from this group.

## 18.3 DSMC Key Responsibilities

The DSMC will be responsible for carrying out the following responsibilities:

- Reviewing and approving the trial protocol and plans for data and safety monitoring
- Assessing data quality, including completeness
- Monitoring recruitment and losses to follow-up
- Monitoring compliance with the treatment protocol by villages and investigators
- Monitoring evidence for intervention differences in the main outcomes
- Monitoring the safety of interventions (e.g., adverse events)
- Deciding whether to recommend that the trial continues as planned or whether intervention should be terminated
- Reviewing proposed major modifications to the study prior to their implementation (e.g., increasing target sample size)
- Suggesting additional data analyses
- Reviewing primary manuscripts prior to submission

## 18.4 DSMC Membership

Members of the DSMC are appointed by Executive Committee of ASANTE after consultation with NEI. The DSMC is an independent multidisciplinary group consisting of the following five members:

Dr. Douglas Jabs: Chair. Ophthalmologist

Dr. Antoinette Darville: Pediatric Infectious disease

Dr. Wondu Alemayehu: Ophthalmologist

Dr. Maureen Maguire: Biostatistician and clinical trialist.

Dr. Paul Emerson: Epidemiologist

The DSMC voting membership includes all five members mentioned above. Non voting members include the members of the ASANTE Executive committee (Dr. Thomas Quinn, and Dr. Sheila West), and members of the ASANTE team as required (Mr. Don Everett, Ms. Beatriz Munoz).

### 18.4.1 DSMC Conflicts of Interest

The DSMC membership is restricted to individuals free of apparent significant conflicts of interest. The source of these conflicts may be financial, scientific or regulatory in nature. Thus, DSMC members may not be involved in ASANTE, have a vested interest in its outcome, have close personal or professional ties to an ASANTE investigator, or have financial investments in Pfizer. If at any time a DSMC member perceives that he/she or another member of the Committee has a potential conflict of interest, he/she is obligated to bring the issue to the attention of the full DSMC for open discussion and resolution. Any DSMC members who develop significant conflicts of interest during the course of trial should resign from the DSMC. The NEI will promptly appoint a replacement.

DSMC members will complete a conflict of interest disclosure form in advance of the first meeting. Competing interests should be disclosed by all DSMC members. The completed forms will be reviewed by the DSMC Chair. If a significant conflict of interest is noted, the DSMC Chair will determine if that a member has a conflict of interest that would compromise their ability to serve on the DSMC. If he recommends resignation, the member will be replaced.

The DSMC membership is to be constituted for the duration of the ASANTE trial. Before the publication of ASANTE trial primary results, the DSMC members should not discuss issues from their involvement in the trial.

### 18.4.2 Relationships

The DSMC is advisory to the NEI and to the ASANTE team. The DSMC will not make decisions about the trials but rather advise the NEI and ASANTE as to the data quality and safety of the intervention throughout the trial.

### 18.4.3 DSMC Meetings

The DSMC will meet once per year, at the start of the project, and once per year thereafter when there is data to review. Additional DSMC teleconferences will be scheduled as the Chair recommends. The agenda will be prepared by the DSMC chair in consultation with the study investigators.

At least five days before the DSMC the Study Principal Investigator will send to the DSMC members a binder of study materials via Federal Express to allow the members time to review the material before the meeting. In keeping with confidentiality, the DSMC members will return the binder of materials to the Principal Investigator at the close of the meeting. The Study Principal Investigator and her team will be responsible for the preparation of meeting materials and all DSMC meeting arrangements.

Open sessions of the DSMC meeting may be attended by DSMC voting members, and the non-voting members, and key study team members from each of the clinical sites. Other members of the ASANTE study team may also be asked by the DSMC chair to attend.

Closed sessions of the DSMC will be attended only by the voting DSMC members, the Executive Committee and the NEI representative. The closed session may be called when the DSMC reviews the safety and impact data unmasked as to random assignment. At the discretion of the DSMC chair, the committee can meet in Executive session with only the five voting members present to discuss issues and take formal votes as needed.

Minutes will be prepared by the Study Principal Investigator. Draft minutes will be combined and distributed to the DSMC chair for review and changes before distribution to the entire committee. The minutes shall summarize all recommendations.

Members must make every effort to attend DSMC meetings. If a member fails to make a meeting, it should be ensured that the member makes the next meeting. If a member fails to make the second meeting, the member will be replaced.

### 18.4.4 Protocol Changes and Ancillary Studies

The DSMC will meet in advance of the start of the trials. The DSMC is charged with reviewing and approving the protocols. The DSMC may review patient oral consent forms, but the institutional and country IRBS have primary responsibility for approval of these forms.

The DSMC will be informed in advance of major study protocol changes. DSMC concurrence will be sought on all substantive recommendations for changes prior to their implementation. Ancillary studies will be brought forward to the DSMC for their information. The DSMC will review them for possible impact on the main trials. The DSMC can make recommendations regarding whether the studies may or may not proceed.

### 18.4.5 DSMC Recommendations

DSMC recommendations will be provided to the study leadership and to the NEI. If the recommendations involve participant safety, the study principal investigator, in collaboration with the site investigators, will act to implement the change as expeditiously as possible. However, if the study leadership does not concur with the DSMC recommendations, it will be the responsibility of the study leadership and DSMC Chair to reach a mutually acceptable decision for implementation, and a report issued to other DSMC members.

### 18.4.6 Contact Information

| Douglas Jabs, M.D., M.B.A.  Chairman, Department of Ophthalmology  Mt Sinai School of Medicine  One Gustave L. Levy Place, Box 1183  New York, NY 10029-6574  Phone: 212-241-6752  Fax: 212-241-5764  e-mail: douglas.jabs@mssm.edu | Maureen Maguire, Ph.D.  University of Pennsylvania  3535 Market Street, Suite 700  Philadelphia, PA 19104-3309  Phone: 215-615-1501  Fax: 215-615-1531  e-mail: maguirem@mail.med.upenn.edu |
| --- | --- |
| Antoinette Darville, M.D.  University of Pittsburgh Physicians Pediatric Infectious Diseases  Keystone Building  3520 Fifth Avenue  Pittsburgh, PA 15213  Phone: 412-692-7885  email: Toni.Darville@chp.edu | Dr. Wondu Alemayehu  Ministry of Health, National Eye Care/  Onchocerciasis Control Program  Samora Avenue, Main Building  P.O. Box 9083  Dar Es Salaam, Tanzania  Phone and fax: 255-222-130009  Email: gracejengo@yahoo.co.uk |
| Dr. Paul Emerson  Director, Trachoma Control Programs  The Carter Center One Copenhill 453 Freedom Parkway Atlanta, GA 30307  Phone: 404-420-5100  Email: paul.emerson@emory.edu |  |

Chapter

19

**Chapter 19: Study Policies**

## 19.1 Introduction

The following are policies established in an effort to maintain the integrity of the ASANTE trial research. The Executive Committee is primarily responsible for setting all policies concerning this trial. Any new policies or changes to current policies will be communicated to all investigators and study staff by the Chairs Office or the Coordinating Center.

## 19.2 Ancillary studies

All ancillary studies will adhere to IRB and HIPPA regulations. To ensure that ancillary studies do not detract from the primary objectives of the ASANTE trial, proposals for ancillary studies are subject to review by the ASANTE Executive Committee and DSMC.

The principal investigator of the ancillary study is expected to report to the Executive Committee progress or termination of the study in yearly intervals. This progress will be reported during each Executive Committee meeting. The Executive Committee retains the right to withdraw the approval of ancillary studies for which no report has been received for one year or for which no progress is reported in two years.

## 19.3 Editorial Policy

The Executive Committee will review all written reports prepared for publication and oral presentations utilizing data from the ASANTE trial. This committee will establish a policy regarding authorship prior to the start of the trial. Each publication and presentation will acknowledge NEI funding by citing cooperative agreements awarded from the NEI of the National Institutes of Health and Human Services.

## 19.4 Access to Study Information

Interested parties must submit a request for access to study data to the ASANTE trial Executive Committee and DSMC. Access to ASANTE trial data for individual study participants is prohibited to individuals without prior HIPPA and IRB approval. The identity of study participants may not be revealed in any public report or presentation. ASANTE trial Manual of Procedures and Study Forms may be accessed by any interested party after prior approval from the Executive Committee.

The following documents may not be released to any group or any outside individual except as needed for National Eye Institute ongoing review:

- Data and safety monitoring reports
- Progress and performance reports
- Minutes of ASANTE trial meetings

Chapter

20

# **Chapter 20: Study Timeline**

The period from September 1, 2012, to January 15, 2013 will be used for planning activities, which include final preparation of the forms and data entry packages, registration of the trial, finalization of the MOP, first meeting of the Executive committee in October, first meeting of the DSMC by end December, planning and organizing the workshop in January, and completion of all necessary IRB submissions to JHU and to Tanzania, and receipt of approvals. KTP would be contacting and recruiting all communities for the trial, and submitting approvals so that communities can be randomized and their intervention status (MDA, newcomer and traveler surveillance and treatment program) can then be discussed with community leadership.

In late January, we plan to hold the training and standardization workshop. From February 1, 2013, to December 2013, we would undertake all the baseline census, surveys and intervention in the 48 communities (as randomized) and half of the 6-month follow-up surveys. From January 15, 2014, to December 2014, we would complete the last half of the 6-month surveys, all the 12-month surveys, and the first half of the 18-month surveys.

From January 15, 2015, to December 2015, we would complete the last half of the 18-month surveys and all the 24-month surveys. From January 15, 2016, to June 2016, we would finalize the last of the specimens, do an orderly shutdown of staff and activities at KTP for this trial, finalize all data cleaning and prepare for submission of all manuscripts from the trial (see Figure 20.1).

As noted earlier, we have two days in each community to schedule examinations. Depending on the month of starting, we have flexibility in the schedule to work around December holiday and rainy spells in January to April. If the rains are heavy, we will alter the schedule of surveys, and allow a window of +/- two months for the community examinations as described above. The six-month examinations must be conducted as they are utilized to determine whether mass treatment is indicated in subsequent years. Note that the schedule will increase in complexity with the addition of the follow-up surveys.

**Figure 20.1. ASANTE Trial Timeline for major activities and outcomes**

Appendix

1

# **MOP Appendix 1: General Study Forms**

Appendix

2

# **MOP Appendix 2: Consent Forms**

Appendix

3

# **MOP Appendix 3: Use of Tablet for data collection**
